# Major depressive disorders in children aged 5–14 years: a Global Burden of Disease analysis from the perspective of exercise psychology

**DOI:** 10.3389/fpubh.2025.1671222

**Published:** 2025-10-29

**Authors:** Wei Wei, Yongsheng Sun, Shuting Yin, Wei Lyu, Rui Liang

**Affiliations:** ^1^Zhengzhou College of Finance and Economics, Zhengzhou, China; ^2^Capital University of Physical Education and Sports, Beijing, China; ^3^Zhengdong New District Education, Culture and Sports Bureau of Zhengzhou City, Zhengzhou, China; ^4^Faculty of Medicine and Health, Al-Farabi Kazakh National University, Almaty, Kazakhstan; ^5^Henan Institute of Science and Technology, Xinxiang, China

**Keywords:** major depressive disorders (MDD), children, GBD (Global Burden of Disease), physical activity, prevalence, incidence, DALYs—disability-adjusted life years

## Abstract

**Background:**

Major depressive disorder (MDD) is an increasingly recognized contributor to morbidity and disability among children globally. While adolescent mental health has received growing attention, the burden and drivers of MDD in children aged 5–14 years remain inadequately characterized at the global level.

**Methods:**

We conducted a comprehensive analysis using Global Burden of Disease (GBD) 2021 data to quantify trends in the prevalence, incidence, and disability-adjusted life years (DALYs) of MDD in children aged 5–14 years across 204 countries and territories from 1990 to 2021. Estimates were stratified by age, sex, region, and Socio-demographic Index (SDI). Key behavioral and psychosocial risk factors were evaluated, and projections to 2035 were generated. Policy and intervention recommendations were developed based on evidence from the literature and global health frameworks.

**Findings:**

Between 1990 and 2021, the global burden of childhood MDD increased substantially, with sharp rises in prevalence and DALY rates, especially among girls and children aged 10–14 years. High-SDI regions exhibited the highest age-standardized rates, while low- and middle-SDI regions showed rapid relative increases. Bullying victimization, physical inactivity, and other modifiable behavioral factors emerged as leading risk factors for childhood MDD. The COVID-19 pandemic acted as a significant accelerant but was not the sole driver of burden growth. Profound disparities in access to mental health services persist, particularly in low-resource settings.

**Interpretation:**

Childhood MDD poses a significant global public health challenge, with profound consequences for lifelong well-being and social functioning. Effective prevention requires school-based mental health initiatives, physical activity interventions, anti-bullying measures, and enhanced community care systems. Mental health policies must ensure equitable resource distribution, robust data infrastructure, and cross-sectoral coordination following WHO and UNICEF guidelines. Improving early identification, mitigating behavioral risks, and guaranteeing universal access to youth mental health services remain crucial for reversing current trajectories and fostering healthy child development.

## Introduction

Mental disorders are among the leading contributors to the global health burden, and onset often occurs in childhood or adolescence (1). According to WHO estimates, approximately 14% of adolescents aged 10–19 experience mental health conditions, contributing to nearly 15% of the total disease burden in this population (2). Among pediatric populations, mental disorders—particularly anxiety, conduct disorders, and depression—have emerged as one of the leading causes of disability. Furthermore, major depressive disorder (MDD) has consistently ranked as the third or fourth most significant mental health contributor to disability-adjusted life years (DALYs) in children, varying by region (3). Childhood-onset depression can profoundly impair cognition, learning, and social functioning, and it greatly increases the risk of recurrent or persistent psychopathology in adulthood (4).

Emerging evidence indicates that the burden of childhood depression is rising rapidly. The COVID-19 pandemic, in particular, has disrupted many social and educational supports for youth (through school closures, isolation, and other stressors) (5). Surveys and meta-analyses report sharp increases in depression and anxiety among young people during the pandemic: by late 2020, roughly 25% of children and adolescents worldwide were experiencing clinically elevated depressive symptoms (and about 20% had elevated anxiety), roughly double prepandemic levels (6). Global Burden of Disease (GBD) data mirror this trend: between 2019 and 2021, the age-standardized prevalence and DALY rates for depressive disorders in under-25s increased by over 10% annually, and depression rose from the fifth- to fourth-ranking cause of DALYs in this age group (7). These increases have been especially pronounced among older children and adolescent girls (8). In short, both epidemiological studies and GBD estimates suggest that the pandemic-era rise in childhood depression is substantial.

Against this backdrop, robust, age-specific global estimates are urgently needed to guide early detection, prevention, and resource allocation. The present study addresses this gap by leveraging the GBD 2021 dataset to quantify the global, regional, and national burden of MDD in children aged 5–14 years from 1990 to 2021. We report trends in prevalence, incidence, and DALYs; examine variation by sex, age subgroup, geography, and sociodemographic development; evaluate risk factor contributions; and generate projections to 2035. Focusing on the 5–14 age group, a critical window before adolescent depression rates escalate sharply, this study offers empirical support for developing child-focused mental health policies and global health strategies.

## Methods

### Overview and methodological details

The GBD data source is recognized as one of the most comprehensive and systematic global epidemiological initiatives. Led by the Institute for Health Metrics and Evaluation (IHME) at the University of Washington, this study aims to quantify health losses attributable to various diseases, injuries, and risk factors (9). The framework enables comparative assessments of morbidity and mortality across different countries, regions, and globally.

Three key metrics are employed in GBD analyses to quantify disease burden: mortality, incidence, and DALYs. DALYs represent the sum of years of life lost (YLL) due to premature mortality and years lived with disability (YLD). The specific calculation formulas are as follows (10):


YLL=Number of deaths×Standard life expectancyatageof death

YLD=Disease prevalence×Disability weight

The 2021 GBD study conducted a comprehensive evaluation of adverse health outcomes associated with 371 diseases, injuries, and disabilities, as well as 88 risk factors (11). The study encompassed 204 countries and territories, utilizing the most recent epidemiological data and refined standardized methodologies. The data used in this study were obtained from the 2021 GBD study.^1^

### Socio-demographic Index

This study employed the SDI to evaluate sociodemographic development by incorporating factors such as income, educational attainment, and fertility patterns in specific regions or countries (12). Based on the calculated SDI scores, regions and countries were stratified into five distinct quintiles: high SDI, high-middle SDI, middle SDI, low-middle SDI, and low SDI. This classification facilitated the investigation of the impact of socioeconomic indices and geographic disparities on the burden of MDD in children.

### Prediction

The ARIMA (autoregressive integrated moving average) model is composed of an autoregressive (AR) model and a moving average (MA) model, with their combination forming the ARIMA framework (13). Its fundamental assumption posits that the data series represents stochastic time-varying variables whose autocorrelation can be characterized by the ARIMA model, thereby enabling the prediction of future values based on historical observations. The model equation is expressed as follows:


$$\begin{array}{c} Y_{t}=\phi_{1}Y_{\left\{t-1\right\}}+\phi_{2}Y_{\left\{t-2\right\}}+\cdots +\phi_{p}Y_{\left\{t-p\right\}}+\varepsilon_{t}-\theta_{1}\varepsilon_{\left\{t-1\right\}}\\ -\theta_{2}\varepsilon_{\left\{t-2\right\}}-\cdots -\theta_{q}\varepsilon_{\left\{t-q\right\}} \end{array}$$

In this equation:

- The component ( $Y_{t}=\phi_{1}Y_{\left\{t-1\right\}}+\phi_{2}Y_{\left\{t-2\right\}}+\ldots +\phi_{p}Y_{\left\{t-p\right\}}+\varepsilon_{t}$ ) constitutes the autoregressive portion.
- The term ( $\varepsilon_{t}-\theta_{1}\varepsilon_{\left\{t-1\right\}}-\theta_{2}\varepsilon_{\left\{t-2\right\}}-\cdots -\theta_{q}\varepsilon_{\left\{t-q\right\}}$ ) represents the moving average component.
- (*Y*_*t*–*p*_) denotes the observed value during the time period (*t*–*p*).
- (*p*) and (*q*) indicate the model orders for the autoregressive and moving average components, respectively.
- (*ε_t_*) signifies the unpredictable deviation occurring within time interval *t* (13).

The ARIMA model requires the time series to be a stochastic sequence with zero mean and exhibit stationarity.

### Meta-analysis

In addition to the GBD analyses, we conducted a complementary meta-analysis of randomized and quasi-experimental trials to quantify the effect of physical activity (PA) interventions on depressive symptoms in youth, thereby linking our epidemiological findings to an exercise-psychology framework.

### Objective

We conducted a complementary meta-analysis to quantify the effect of PA interventions on depressive symptoms in children/adolescents.

### Search strategy

Only English-language studies were eligible for full-text review and were included in the analyses. The search combined MeSH headings with free-text terms covering the population (“child,” “adolescent”), condition (“depression”), and intervention (“exercise,” “training,” “physical activity”).

### Data extraction and outcome

Two reviewers independently extracted study characteristics and post-intervention means/SDs (or change scores); discrepancies were resolved by consensus. The primary outcome was the standardized mean difference in depressive symptoms (negative values favor PA). The specific details are provided in Appendix 1.

### Results overview

The pooled effect indicated that PA reduces depressive symptoms in youth (SMD − 0.37, 95% CI −0.59 to −0.15; *I*^2^ = 76%; *p* < 0.001).

### Statistical analysis

In this study, data on the prevalence, incidence, and DALYs of MDD among individuals aged 5–14 years were collected and analyzed at the global, regional, and national levels. All estimates are presented with their corresponding 95% uncertainty intervals (UIs). Additionally, prevalence, incidence, and DALYs were reported per 100,000 population annually. The methodology employed in the GBD study 2021 has been described in detail elsewhere (9, 14). The average annual percentage change (AAPC) and annual percentage change (APC) were used to assess trends in variation (15). A log-transformed linear regression model was employed to calculate the estimated average annual percentage change (EAPC) and its confidence interval (CI) for analyzing temporal trends in the prevalence, incidence rate, and DALYs of MDD in children from 1990 to 2021 (16). The EAPC is particularly valuable for examining long-term trends, as it untangles whether the occurrence rate generally increases or decreases over time, irrespective of short-term fluctuations. An EAPC value and the lower bound of its 95% CI greater than 0 indicate an upward trend in the corresponding metric. Conversely, an EAPC value and the upper bound of its 95% CI less than 0 suggest a downward trend. All statistical analyses and graphical representations were processed using R version 4.4.2. The following R packages facilitated time series analysis and modeling: the “forecast” package performed automated ARIMA modeling, forecasting, and diagnostic testing; the “tseries” package supplied tools for time series analysis, including the augmented Dickey–Fuller (ADF) test; and “ggplot2” enabled visualization of time series data and forecasting outputs. Meta-analyses and forest plots were generated using the “metafor” and “meta” packages, while “dplyr,” “readxl,” and “tidyr” supported data manipulation and visualization.

## Results

### Globa trends

#### Prevalence

**Figure 1:**
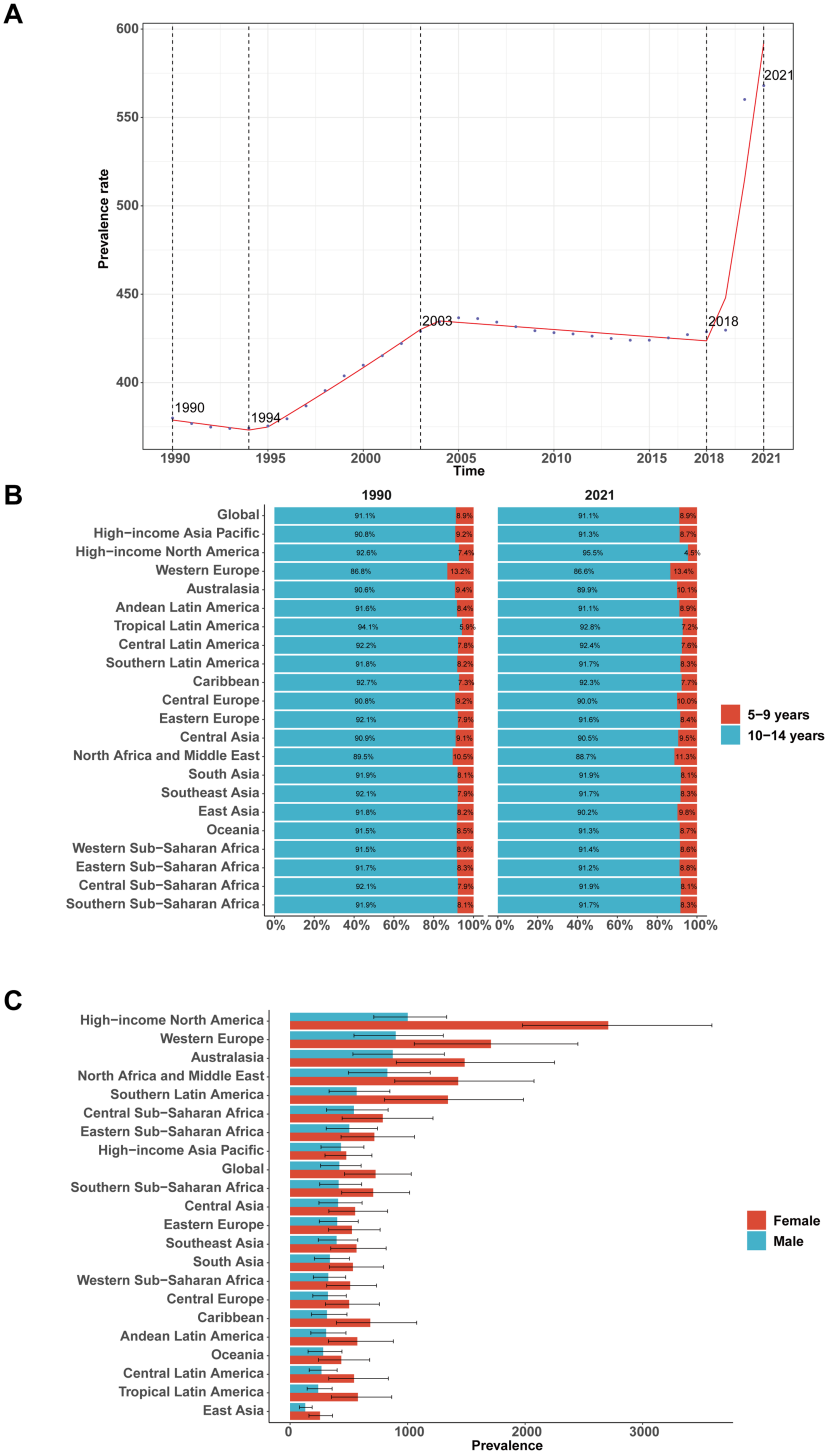
Global and regional prevalence of major depressive disorder among children aged 5–14 years, 1990–2021. **(A)** Global prevalence rate over time. **(B)** Composition of prevalence by age group (5–9 vs. 10–14 years) in 1990 and 2021 across GBD regions. **(C)** Sex-specific prevalence by region in 2021.

Analysis of the GBD 2021 dataset reveals pronounced dynamic changes in the global prevalence of childhood MDD. From 2018 to 2021, prevalence increased sharply, with an APC of 14.97% (95% CI, 11.60–18.45%; Figure 1A). A peak was observed in 2021, when prevalence reached 568.00 per 100,000 (95% UI, 358.61–810.57). The global number of MDD cases among children aged 5–14 years increased markedly from 4.25 million (95% UI, 2.69–6.10 million) in 1990 to 7.69 million (95% UI, 4.85–10.97 million) in 2021—an 81% increase (95% UI, 76.17–85.92%). The corresponding prevalence rate rose by 49% [from 379.96 (95% UI, 240.57–544.91) to 568.00 (95% UI, 358.61–810.57) per 100,000], with an EAPC of 0.79% (95% CI, 0.55–1.03%; Table 1). Notably, prevalence was substantially higher among children aged 10–14 years (1047.80 per 100,000) compared to those aged 5–9 years (102.46 per 100,000) in 2021 (Figure 1B). Across all regions, girls had a consistently higher prevalence than boys (727.28 vs. 418.46 per 100,000, respectively; Figure 1C).

**Table 1:** Prevalence of major depressive disorders in children between 1990 and 2021 at the global and regional level.

	1990		2021		1990–2021		
Location	Prevalent cases	Prevalence rate	Prevalent cases	Prevalence rate	Cases change^b^	Rate change^b^	EAPC^a^
Global	503850.35 (322147.07, 720374.25)	235.35 (150.48, 336.49)	350906.82 (218830.99, 503176.55)	187.37 (116.85, 268.68)	−30.35 (−35.59, −24.23)	−20.39 (−26.37, −13.39)	−0.34 (−0.78, 0.11)
High SDI	98882.25 (61963.62, 142201.37)	288.96 (181.08, 415.55)	116924.25 (72493.25, 167704.88)	461.69 (286.25, 662.21)	18.25 (12.15, 26.13)	59.78 (51.53, 70.44)	−0.16 (−0.64, 0.33)
High-middle SDI	250516.51 (155038.82, 364793.57)	459.80 (284.56, 669.54)	697591.02 (420453.92, 1016626.66)	608.53 (366.78, 886.84)	178.46 (158.34, 199.72)	32.35 (22.78, 42.45)	−0.01 (−0.28, 0.25)
Middle SDI	80198.96 (50562.17, 115808.48)	321.01 (202.38, 463.54)	72617.71 (44539.57, 107208.19)	454.62 (278.84, 671.17)	−9.45 (−17.51, −0.23)	41.62 (29.02, 56.05)	0.77 (0.44, 1.11)
Low-middle SDI	320997.72 (211305.74, 441839.70)	802.58 (528.32, 1104.71)	827896.16 (601026.57, 1094614.75)	1834.86 (1332.05, 2425.99)	157.91 (130.05, 210.23)	128.62 (103.93, 174.99)	2.38 (1.90, 2.87)
Low SDI	712693.08 (435146.74, 1031466.28)	798.48 (487.53, 1155.63)	1367657.64 (835263.65, 1971043.83)	1119.33 (683.61, 1613.16)	91.90 (77.56, 109.34)	40.18 (29.71, 52.92)	0.56 (0.33, 0.80)
Regions	5328.89 (3147.63, 8044.92)	318.01 (187.84, 480.10)	11152.99 (6217.44, 17349.73)	354.47 (197.60, 551.41)	109.29 (70.85, 149.69)	11.46 (−9.01, 32.98)	−0.01 (−0.12, 0.10)
Andean Latin America	673311.87 (419483.27, 984340.10)	243.66 (151.80, 356.21)	1505984.19 (933668.79, 2241119.92)	432.22 (267.97, 643.21)	123.67 (110.44, 136.54)	77.39 (66.90, 87.60)	1.39 (1.06, 1.73)
Australasia	358727.92 (221819.83, 521234.71)	318.99 (197.25, 463.49)	555777.23 (341336.67, 805222.99)	477.60 (293.32, 691.96)	54.93 (46.74, 63.82)	49.72 (41.81, 58.31)	0.59 (0.33, 0.84)
Caribbean	61778.29 (37671.55, 90968.70)	631.71 (385.21, 930.20)	96725.03 (57542.19, 144184.10)	946.70 (563.20, 1411.21)	56.57 (30.46, 85.66)	49.86 (24.87, 77.71)	0.46 (0.11, 0.82)
Central Asia	45900.93 (28599.65, 67410.26)	347.30 (216.39, 510.05)	89631.94 (55466.77, 129512.29)	558.92 (345.87, 807.60)	95.27 (81.21, 111.06)	60.93 (49.34, 73.94)	0.46 (0.08, 0.84)
Central Europe	114854.61 (71507.77, 172337.29)	314.37 (195.73, 471.71)	133297.40 (81219.03, 200752.88)	404.11 (246.23, 608.61)	16.06 (7.37, 25.72)	28.55 (18.92, 39.24)	−0.98 (−2.03, 0.10)
Central Latin America	460913.25 (314700.21, 624877.84)	959.01 (654.79, 1300.16)	606349.28 (370181.32, 864017.99)	1293.16 (789.48, 1842.68)	31.55 (14.45, 46.82)	34.84 (17.31, 50.49)	0.31 (−0.04, 0.66)
Central Sub-Saharan Africa	193242.99 (119152.36, 278734.47)	370.66 (228.54, 534.64)	562104.10 (344337.45, 807272.10)	416.97 (255.43, 598.84)	190.88 (176.60, 205.59)	12.50 (6.97, 18.19)	0.16 (−0.01, 0.33)
East Asia	503850.35 (322147.07, 720374.25)	235.35 (150.48, 336.49)	350906.82 (218830.99, 503176.55)	187.37 (116.85, 268.68)	−30.35 (−35.59, −24.23)	−20.39 (−26.37, −13.39)	−0.34 (−0.78, 0.11)
Eastern Europe	98882.25 (61963.62, 142201.37)	288.96 (181.08, 415.55)	116924.25 (72493.25, 167704.88)	461.69 (286.25, 662.21)	18.25 (12.15, 26.13)	59.78 (51.53, 70.44)	−0.16 (−0.64, 0.33)
Eastern Sub-Saharan Africa	250516.51 (155038.82, 364793.57)	459.80 (284.56, 669.54)	697591.02 (420453.92, 1016626.66)	608.53 (366.78, 886.84)	178.46 (158.34, 199.72)	32.35 (22.78, 42.45)	−0.01 (−0.28, 0.25)
High-income Asia Pacific	80198.96 (50562.17, 115808.48)	321.01 (202.38, 463.54)	72617.71 (44539.57, 107208.19)	454.62 (278.84, 671.17)	−9.45 (−17.51, −0.23)	41.62 (29.02, 56.05)	0.77 (0.44, 1.11)
High-income North America	320997.72 (211305.74, 441839.70)	802.58 (528.32, 1104.71)	827896.16 (601026.57, 1094614.75)	1834.86 (1332.05, 2425.99)	157.91 (130.05, 210.23)	128.62 (103.93, 174.99)	2.38 (1.90, 2.87)
North Africa and Middle East	712693.08 (435146.74, 1031466.28)	798.48 (487.53, 1155.63)	1367657.64 (835263.65, 1971043.83)	1119.33 (683.61, 1613.16)	91.90 (77.56, 109.34)	40.18 (29.71, 52.92)	0.56 (0.33, 0.80)
Oceania	5328.89 (3147.63, 8044.92)	318.01 (187.84, 480.10)	11152.99 (6217.44, 17349.73)	354.47 (197.60, 551.41)	109.29 (70.85, 149.69)	11.46 (−9.01, 32.98)	−0.01 (−0.12, 0.10)
South Asia	673311.87 (419483.27, 984340.10)	243.66 (151.80, 356.21)	1505984.19 (933668.79, 2241119.92)	432.22 (267.97, 643.21)	123.67 (110.44, 136.54)	77.39 (66.90, 87.60)	1.39 (1.06, 1.73)
Southeast Asia	358727.92 (221819.83, 521234.71)	318.99 (197.25, 463.49)	555777.23 (341336.67, 805222.99)	477.60 (293.32, 691.96)	54.93 (46.74, 63.82)	49.72 (41.81, 58.31)	0.59 (0.33, 0.84)
Southern Latin America	61778.29 (37671.55, 90968.70)	631.71 (385.21, 930.20)	96725.03 (57542.19, 144184.10)	946.70 (563.20, 1411.21)	56.57 (30.46, 85.66)	49.86 (24.87, 77.71)	0.46 (0.11, 0.82)
Southern Sub-Saharan Africa	45900.93 (28599.65, 67410.26)	347.30 (216.39, 510.05)	89631.94 (55466.77, 129512.29)	558.92 (345.87, 807.60)	95.27 (81.21, 111.06)	60.93 (49.34, 73.94)	0.46 (0.08, 0.84)
Tropical Latin America	114854.61 (71507.77, 172337.29)	314.37 (195.73, 471.71)	133297.40 (81219.03, 200752.88)	404.11 (246.23, 608.61)	16.06 (7.37, 25.72)	28.55 (18.92, 39.24)	−0.98 (−2.03, 0.10)
Western Europe	460913.25 (314700.21, 624877.84)	959.01 (654.79, 1300.16)	606349.28 (370181.32, 864017.99)	1293.16 (789.48, 1842.68)	31.55 (14.45, 46.82)	34.84 (17.31, 50.49)	0.31 (−0.04, 0.66)

EAPC, estimated annual percentage change; SDI, Socio-demographic Index; UI, uncertainty interval.

a EAPC is expressed as 95% confidence interval.

b Change shows the percentage change.

#### Incidence

**Figure 2:**
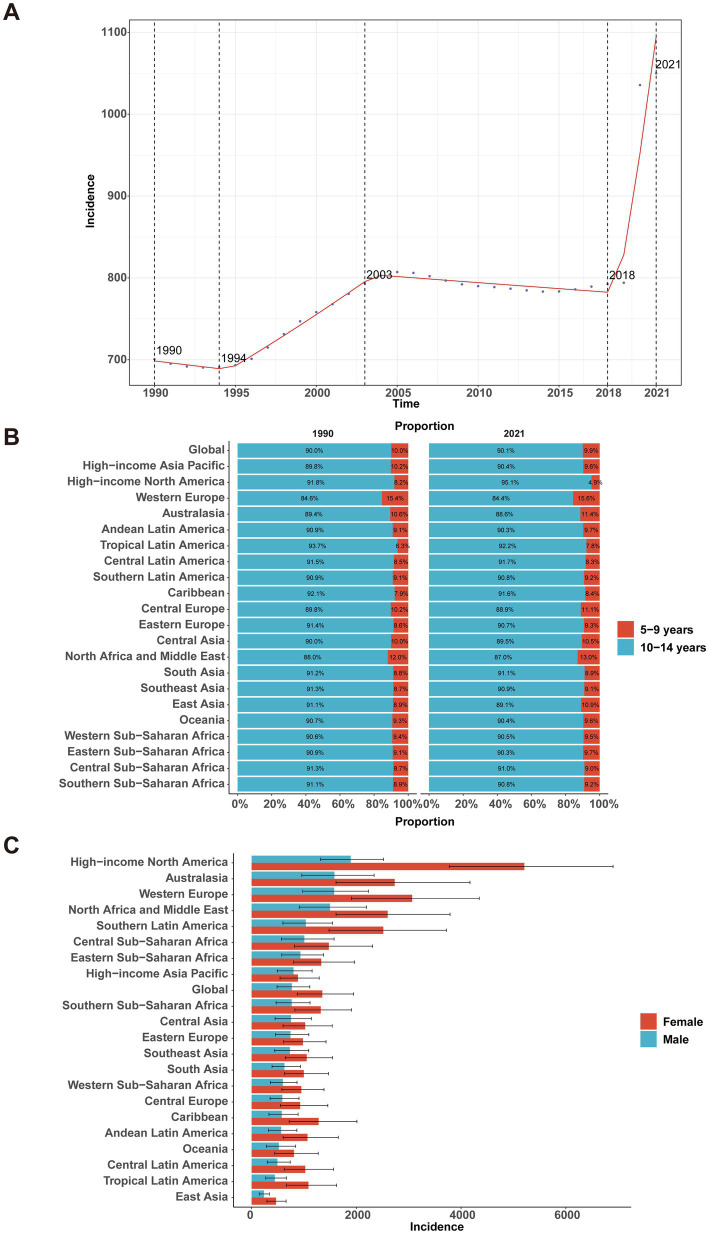
Global and regional incidence of major depressive disorder among children aged 5–14 years, 1990–2021. **(A)** Global incidence rate over time. **(B)** Composition of incidence by age group (5–9 vs. 10–14 years) in 1990 and 2021 across GBD regions. **(C)** Sex-specific incidence by region in 2021.

The incidence of childhood MDD paralleled prevalence trends, with a sharp rise from 2018 to 2021 (APC = 14.99, 95% CI, 11.59–18.49; Figure 2A). Incidence peaked in 2021 at 1049.92 per 100,000 (95% UI, 675.33–1514.67). Globally, incident cases rose from 7.84 million (95% UI, 5.02–11.28 million) in 1990 to 14.21 million (95% UI, 9.14–20.50 million) in 2021—an increase of 81% (95% UI, 76.67–86.64%). The incidence rate increased by 50% [from 700.56 (95% UI, 448.95–1007.84) to 1049.92 (95% UI, 675.33–1514.67) per 100,000], with an EAPC of 0.79% (95% CI, 0.55–1.03%; Table 2). Incidence among children aged 10–14 years was over nine times higher than that among those aged 5–9 years in 2021 [1915.15 (95% UI, 1188.21–2854.79) vs. 210.41 per 100,000; Figure 2B]. Girls exhibited a higher incidence rate than boys (1349.71 vs. 768.46 per 100,000), with the sex gap consistent across regions (Figure 2C).

**Table 2:** Incidence of major depressive disorders in children between 1990 and 2021 at the global and regional level.

	1990		2021		1990–2021		
Location	Incident cases	Incidence rate	Incident cases	Incidence rate	Cases change^b^	Rate change^b^	EAPC^a^
Global	7840812.08 (5024745.69, 11279861.95)	700.56 (448.95, 1007.84)	14212713.19 (9141833.95, 20503955.61)	1049.92 (675.33, 1514.67)	81.27 (76.67, 86.64)	49.87 (46.07, 54.31)	0.79 (0.55, 1.03)
High SDI	1433972.67 (931498.55, 2012697.36)	1155.54 (750.63, 1621.89)	2698025.84 (1847670.41, 3697796.22)	2273.16 (1556.72, 3115.50)	88.15 (74.39, 111.30)	96.72 (82.34, 120.93)	1.72 (1.38, 2.07)
High-middle SDI	1405001.45 (925020.94, 1977030.62)	777.44 (511.85, 1093.97)	1724999.06 (1077275.70, 2486590.84)	1072.46 (669.76, 1545.95)	22.78 (12.13, 35.79)	37.95 (25.98, 52.57)	0.98 (0.78, 1.18)
Middle SDI	2169756.01 (1369887.32, 3143444.07)	576.02 (363.68, 834.52)	3137382.86 (1954302.33, 4541378.37)	803.95 (500.79, 1163.73)	44.60 (39.90, 49.51)	39.57 (35.03, 44.31)	0.43 (0.16, 0.69)
Low-middle SDI	1806853.04 (1126937.15, 2635088.71)	605.05 (377.37, 882.39)	3723005.35 (2304548.44, 5463607.20)	958.89 (593.55, 1407.19)	106.05 (95.63, 117.10)	58.48 (50.46, 66.98)	0.86 (0.58, 1.14)
Low SDI	1018674.30 (627515.71, 1492913.93)	737.54 (454.33, 1080.90)	2918790.08 (1795851.56, 4335142.61)	990.59 (609.49, 1471.28)	186.53 (173.51, 202.27)	34.31 (28.21, 41.69)	0.36 (0.15, 0.58)
Regions							
Andean Latin America	43006.07 (24525.75, 65960.71)	449.36 (256.26, 689.21)	96279.62 (54907.91, 147901.75)	806.43 (459.90, 1238.81)	123.87 (92.40, 161.56)	79.46 (54.23, 109.67)	0.74 (0.19, 1.28)
Australasia	54174.92 (33943.24, 77136.53)	1779.91 (1115.20, 2534.32)	83778.65 (49715.91, 126347.61)	2139.89 (1269.85, 3227.19)	54.64 (23.77, 92.81)	20.22 (−3.78, 49.89)	0.57 (0.43, 0.71)
Caribbean	49265.80 (28744.43, 74177.83)	676.63 (394.79, 1018.79)	70454.15 (40008.12, 110145.22)	922.55 (523.88, 1442.27)	43.01 (27.68, 59.71)	36.34 (21.73, 52.27)	0.18 (−0.11, 0.47)
Central Asia	99791.21 (60792.39, 147788.66)	645.20 (393.05, 955.53)	155855.32 (92384.17, 237893.76)	881.60 (522.57, 1345.65)	56.18 (38.66, 74.15)	36.64 (21.31, 52.36)	0.23 (−0.07, 0.53)
Central Europe	96711.38 (57820.67, 144335.05)	475.19 (284.10, 709.19)	90931.74 (54292.69, 141318.00)	750.54 (448.12, 1166.42)	−5.98 (−12.98, 1.91)	57.94 (46.19, 71.19)	0.10 (−0.35, 0.56)
Central Latin America	192202.13 (119170.09, 284522.68)	464.67 (288.11, 687.86)	326760.74 (200126.03, 497982.66)	753.00 (461.18, 1147.57)	70.01 (59.86, 80.14)	62.05 (52.38, 71.71)	0.64 (0.28, 1.00)
Central Sub-Saharan Africa	154025.59 (90163.68, 231972.28)	1032.74 (604.55, 1555.38)	465571.14 (263077.36, 728077.55)	1237.72 (699.39, 1935.60)	202.27 (149.91, 258.60)	19.85 (−0.91, 42.18)	0.19 (−0.04, 0.42)
East Asia	935583.40 (596568.68, 1347981.15)	437.01 (278.66, 629.65)	643751.61 (400069.78, 914043.32)	343.74 (213.62, 488.07)	−31.19 (−36.22, −24.98)	−21.34 (−27.09, −14.24)	−0.37 (−0.81, 0.07)
Eastern Europe	184033.50 (115476.30, 264740.61)	537.80 (337.45, 773.65)	217180.97 (135459.06, 314801.53)	857.57 (534.88, 1243.04)	18.01 (11.88, 25.90)	59.46 (51.17, 70.12)	−0.16 (−0.64, 0.33)
Eastern Sub-Saharan Africa	465224.25 (288895.35, 682871.25)	853.87 (530.24, 1253.34)	1292077.84 (786513.87, 1905660.02)	1127.12 (686.10, 1662.37)	177.73 (157.20, 198.65)	32.00 (22.24, 41.95)	−0.03 (−0.30, 0.24)
High-income Asia Pacific	147710.89 (92863.84, 212934.62)	591.23 (371.70, 852.30)	134356.64 (82089.07, 196118.84)	841.13 (513.91, 1227.78)	−9.04 (−17.36, 0.07)	42.27 (29.25, 56.52)	0.80 (0.46, 1.13)
High-income North America	597846.47 (395944.33, 825818.63)	1494.77 (989.96, 2064.76)	1584560.81 (1140397.32, 2101491.35)	3511.85 (2527.46, 4657.52)	165.04 (134.83, 221.89)	134.94 (108.16, 185.33)	2.47 (1.97, 2.98)
North Africa and Middle East	1299019.73 (797768.47, 1871233.70)	1455.39 (893.80, 2096.48)	2479392.66 (1526511.57, 3623746.74)	2029.21 (1249.35, 2965.79)	90.87 (76.83, 107.69)	39.43 (29.17, 51.72)	0.53 (0.30, 0.77)
Oceania	9886.02 (5853.57, 14991.65)	589.97 (349.33, 894.66)	20668.35 (11405.83, 32586.55)	656.89 (362.50, 1035.67)	109.07 (71.13, 149.64)	11.34 (−8.86, 32.95)	−0.02 (−0.12, 0.09)
South Asia	1254208.41 (789764.30, 1842509.59)	453.87 (285.80, 666.76)	2801724.52 (1767566.67, 4144234.05)	804.10 (507.30, 1189.41)	123.39 (110.44, 136.78)	77.17 (66.90, 87.79)	1.39 (1.06, 1.73)
Southeast Asia	667065.19 (415741.01, 956279.82)	593.17 (369.69, 850.34)	1032417.82 (623334.75, 1527874.19)	887.19 (535.65, 1312.96)	54.77 (46.34, 63.12)	49.57 (41.42, 57.64)	0.59 (0.34, 0.85)
Southern Latin America	114737.74 (70836.80, 168470.99)	1173.25 (724.34, 1722.70)	179774.01 (106379.27, 264702.56)	1759.55 (1041.19, 2590.79)	56.68 (30.34, 87.20)	49.97 (24.76, 79.19)	0.46 (0.11, 0.81)
Southern Sub-Saharan Africa	85343.57 (53373.43, 125151.41)	645.74 (403.84, 946.94)	166776.16 (103088.96, 241817.33)	1039.96 (642.83, 1507.90)	95.42 (80.87, 110.35)	61.05 (49.06, 73.36)	0.47 (0.09, 0.85)
Tropical Latin America	217116.58 (135570.19, 320197.74)	594.27 (371.07, 876.42)	249796.21 (152661.15, 373987.65)	757.29 (462.81, 1133.80)	15.05 (6.31, 24.29)	27.43 (17.75, 37.67)	−1.01 (−2.05, 0.05)
Western Europe	815313.83 (553898.80, 1102959.46)	1696.39 (1152.48, 2294.89)	1078883.52 (672006.50, 1528797.09)	2300.93 (1433.18, 3260.45)	32.33 (14.44, 48.18)	35.64 (17.31, 51.88)	0.29 (−0.06, 0.65)
Western Sub-Saharan Africa	358545.40 (219830.53, 524597.01)	687.72 (421.65, 1006.22)	1041720.71 (635139.87, 1511202.81)	772.76 (471.15, 1121.02)	190.54 (176.03, 204.62)	12.37 (6.76, 17.81)	0.16 (−0.01, 0.33)

EAPC, estimated annual percentage change; SDI, Socio-demographic Index; UI, uncertainty interval.

a EAPC is expressed as 95% confidence interval.

b Change shows the percentage change.

#### DALYs

**Figure 3:**
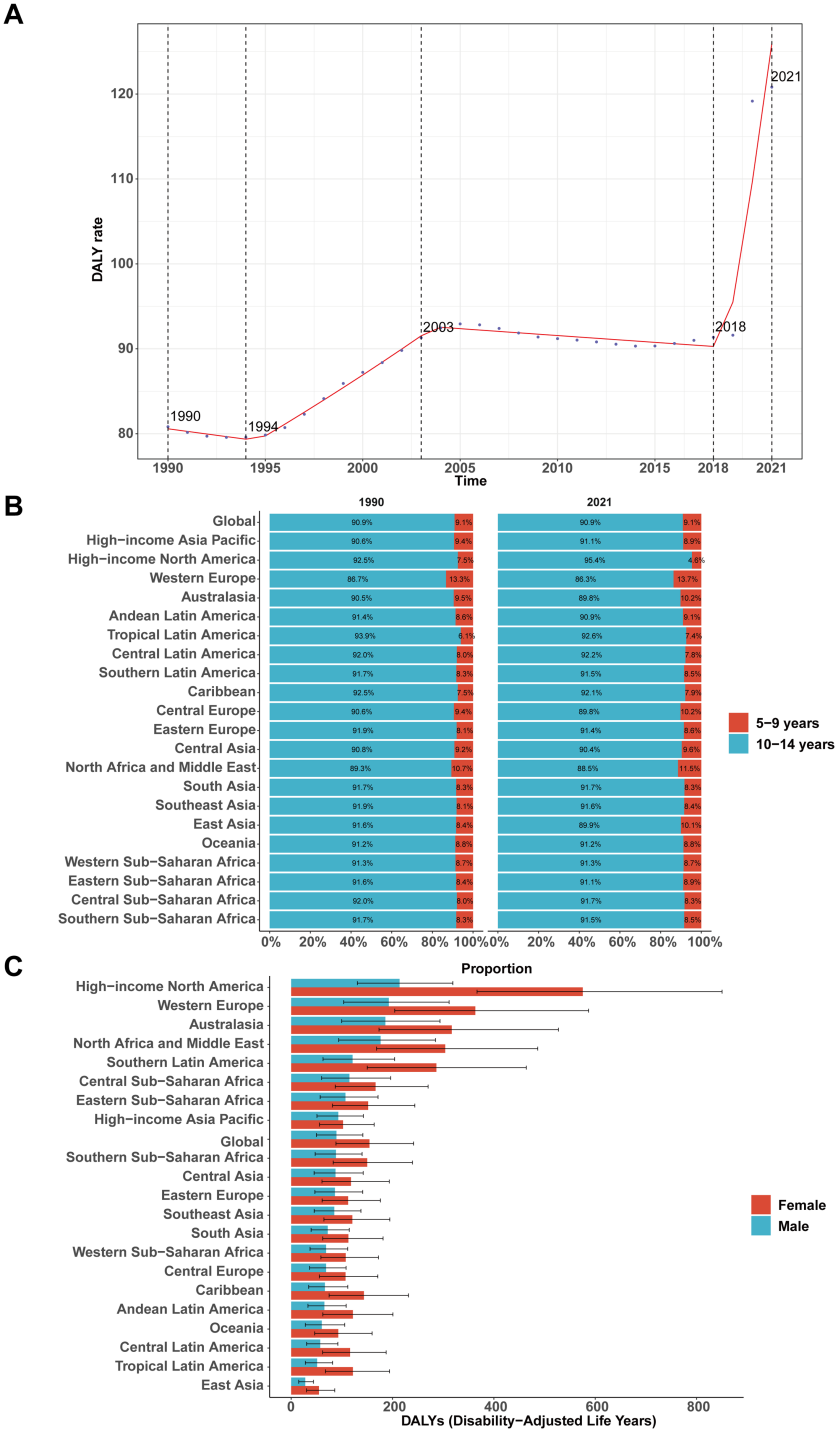
Global and regional disability-adjusted life years (DALYs) of major depressive disorder among children aged 5–14 years, 1990–2021. **(A)** Global DALY rate over time. **(B)** Composition of DALYs by age group (5–9 vs. 10–14 years) in 1990 and 2021 across GBD regions. **(C)** Sex-specific DALYs by region in 2021.

Patterns in DALYs mirrored those of prevalence and incidence, with a notable increase between 2018 and 2021 (APC = 14.84, 95% CI, 11.50–18.29; Figure 3A). The global DALY rate peaked at 120.82 per 100,000 (95% UI, 68.69–189.71) in 2021. Total DALYs rose from 904,594 (95% UI, 506,817–1,417,069) in 1990 to 1,635,466 (95% UI, 929,884–2,568,019) in 2021, an increase of 81% (95% UI, 75.84–86.81%). The DALY rate increased by 49% [from 80.82 (95% UI, 45.28–126.61) to 120.82 (95% UI, 68.69–189.71) per 100,000], with an EAPC of 0.80% (95% CI, 0.56–1.04%; Table 3). Children aged 10–14 years experienced a much higher DALY rate than those aged 5–9 years (222.44 vs. 22.21 per 100,000; Figure 3B). Globally, girls had a higher DALY rate than boys (154.41 vs. 89.27 per 100,000), with the sex difference most pronounced in high-income North America (575.33 vs. 213.80; Figure 3C).

**Table 3:** DALYs of major depressive disorders in children between 1990 and 2021 at the global and regional level.

	1990		2021		1990–2021		
Location	DALY cases	DALY rate	DALY cases	DALY rate	Cases change^b^	Rate change^b^	EAPC^a^
Global	904594.05 (506816.72, 1417068.83)	80.82 (45.28, 126.61)	1635466.05 (929883.99, 2568019.10)	120.82 (68.69, 189.71)	80.80 (75.84, 86.81)	49.48 (45.39, 54.45)	0.80 (0.56, 1.04)
High SDI	167099.66 (96420.01, 260936.76)	134.65 (77.70, 210.27)	307814.59 (184649.22, 456446.85)	259.34 (155.57, 384.57)	84.21 (70.98, 104.33)	92.60 (78.77, 113.64)	1.64 (1.31, 1.98)
High-middle SDI	165289.70 (95527.83, 254668.06)	91.46 (52.86, 140.92)	203479.25 (113758.39, 320058.36)	126.51 (70.73, 198.98)	23.10 (11.29, 36.50)	38.32 (25.04, 53.37)	1.03 (0.82, 1.24)
Middle SDI	249257.72 (139487.75, 393118.49)	66.17 (37.03, 104.36)	361798.11 (203521.85, 574035.25)	92.71 (52.15, 147.10)	45.15 (38.90, 52.17)	40.10 (34.07, 46.88)	0.45 (0.18, 0.71)
Low-middle SDI	206594.58 (112331.74, 331191.11)	69.18 (37.62, 110.90)	427282.09 (231261.49, 683720.65)	110.05 (59.56, 176.10)	106.82 (93.93, 122.24)	59.07 (49.16, 70.93)	0.89 (0.61, 1.16)
Low SDI	115594.48 (61479.51, 186856.75)	83.69 (44.51, 135.29)	333876.05 (180904.80, 530874.57)	113.31 (61.40, 180.17)	188.83 (171.64, 207.67)	35.39 (27.33, 44.22)	0.40 (0.19, 0.61)
Regions							
Andean Latin America	4922.55 (2537.62, 8225.00)	51.43 (26.51, 85.94)	11065.90 (5657.34, 18049.42)	92.69 (47.39, 151.18)	124.80 (84.64, 173.82)	80.20 (48.01, 119.50)	0.76 (0.22, 1.30)
Australasia	6294.72 (3371.78, 9712.93)	206.81 (110.78, 319.12)	9764.40 (5365.51, 15845.55)	249.40 (137.05, 404.73)	55.12 (22.28, 93.68)	20.59 (−4.94, 50.57)	0.56 (0.43, 0.70)
Caribbean	5596.46 (2989.51, 9291.25)	76.86 (41.06, 127.61)	7967.53 (4197.49, 12985.95)	104.33 (54.96, 170.04)	42.37 (23.28, 64.26)	35.73 (17.53, 56.61)	0.19 (−0.09, 0.48)
Central Asia	11528.47 (6280.95, 18490.80)	74.54 (40.61, 119.55)	18045.07 (9265.54, 29287.71)	102.07 (52.41, 165.67)	56.53 (36.55, 79.34)	36.94 (19.46, 56.90)	0.25 (−0.05, 0.55)
Central Europe	11288.61 (6070.29, 18102.37)	55.47 (29.83, 88.95)	10585.86 (5524.69, 16772.33)	87.37 (45.60, 138.44)	−6.23 (−15.09, 4.20)	57.53 (42.63, 75.03)	0.11 (−0.34, 0.57)
Central Latin America	21998.54 (12053.70, 35125.15)	53.18 (29.14, 84.92)	37380.88 (20372.05, 60247.26)	86.14 (46.95, 138.84)	69.92 (55.02, 84.86)	61.97 (47.77, 76.21)	0.65 (0.29, 1.00)
Central Sub-Saharan Africa	17250.21 (8939.47, 28524.92)	115.66 (59.94, 191.26)	52790.47 (27382.92, 87273.04)	140.34 (72.80, 232.02)	206.03 (141.70, 278.38)	21.34 (−4.17, 50.02)	0.22 (−0.01, 0.44)
East Asia	108264.24 (61656.00, 167962.47)	50.57 (28.80, 78.46)	75494.43 (41495.33, 118099.23)	40.31 (22.16, 63.06)	−30.27 (−37.77, −22.24)	−20.29 (−28.86, −11.11)	−0.32 (−0.77, 0.12)
Eastern Europe	21184.83 (11611.54, 33790.76)	61.91 (33.93, 98.75)	25056.56 (13807.37, 40232.00)	98.94 (54.52, 158.86)	18.28 (9.00, 29.03)	59.82 (47.29, 74.34)	−0.15 (−0.63, 0.34)
Eastern Sub-Saharan Africa	52787.53 (28392.74, 84268.86)	96.89 (52.11, 154.67)	147927.39 (80597.37, 237450.17)	129.04 (70.31, 207.14)	180.23 (158.41, 206.50)	33.19 (22.82, 45.67)	0.02 (−0.25, 0.28)
High-income Asia Pacific	17153.45 (9364.19, 26624.24)	68.66 (37.48, 106.57)	15558.61 (8494.79, 23914.90)	97.40 (53.18, 149.72)	−9.30 (−20.35, 2.63)	41.87 (24.58, 60.53)	0.78 (0.45, 1.12)
High-income North America	68566.22 (40602.09, 102994.90)	171.43 (101.52, 257.51)	176207.18 (110509.18, 259697.76)	390.53 (244.92, 575.57)	156.99 (128.67, 208.05)	127.80 (102.70, 173.06)	2.37 (1.89, 2.86)
North Africa and Middle East	151660.62 (82090.36, 243655.27)	169.92 (91.97, 272.99)	290960.45 (157590.27, 465431.29)	238.13 (128.98, 380.92)	91.85 (74.50, 110.89)	40.15 (27.47, 54.05)	0.57 (0.34, 0.80)
Oceania	1130.18 (586.62, 1833.42)	67.45 (35.01, 109.41)	2384.36 (1196.97, 4039.19)	75.78 (38.04, 128.37)	110.97 (63.10, 167.83)	12.36 (−13.14, 42.64)	0.02 (−0.09, 0.12)
South Asia	142177.24 (77656.08, 227627.34)	51.45 (28.10, 82.37)	319185.16 (176118.53, 507470.29)	91.61 (50.55, 145.65)	124.50 (108.39, 146.31)	78.05 (65.28, 95.34)	1.41 (1.08, 1.74)
Southeast Asia	76554.73 (41935.64, 123327.34)	68.07 (37.29, 109.67)	118951.42 (63827.46, 192884.44)	102.22 (54.85, 165.75)	55.38 (45.53, 68.12)	50.16 (40.64, 62.47)	0.60 (0.35, 0.86)
Southern Latin America	13210.41 (7065.52, 20913.02)	135.08 (72.25, 213.85)	20650.69 (10977.16, 33252.93)	202.12 (107.44, 325.46)	56.32 (27.48, 91.53)	49.63 (22.02, 83.33)	0.47 (0.12, 0.82)
Southern Sub-Saharan Africa	9745.35 (5414.58, 15418.55)	73.74 (40.97, 116.66)	19065.51 (10610.82, 30122.53)	118.89 (66.17, 187.83)	95.64 (75.74, 114.13)	61.23 (44.84, 76.47)	0.47 (0.09, 0.85)
Tropical Latin America	24269.74 (13333.16, 37663.83)	66.43 (36.49, 103.09)	28258.09 (15480.26, 44982.13)	85.67 (46.93, 136.37)	16.43 (5.20, 28.68)	28.96 (16.52, 42.53)	−0.96 (−2.02, 0.11)
Western Europe	98298.82 (57462.31, 153203.80)	204.53 (119.56, 318.77)	129273.69 (71297.93, 208027.97)	275.70 (152.06, 443.66)	31.51 (13.76, 47.32)	34.80 (16.60, 51.00)	0.31 (−0.04, 0.66)
Western Sub-Saharan Africa	40711.13 (22035.67, 64796.56)	78.09 (42.27, 124.29)	118892.41 (64301.66, 191777.27)	88.20 (47.70, 142.26)	192.04 (173.31, 212.77)	12.94 (5.70, 20.96)	0.18 (0.01, 0.35)

EAPC, estimated annual percentage change; DALYs, disability adjusted life years; SDI, Socio-demographic Index; UI, uncertainty interval.

a EAPC is expressed as 95% confidence interval.

b Change shows the percentage change.

### SDI regional trends

In 2021, the largest number of prevalent cases occurred in low-middle SDI regions (2,013,364, 95% UI, 1,226,768–2,963,052), but prevalence rate was highest in high SDI regions (1217.42 per 100,000, 95% UI, 840.64–1670.12; EAPC = 1.65, 95% CI, 1.31–1.99%; Table 1). Similarly, the highest number of incident cases was observed in low-middle SDI regions (3,723,005, 95% UI, 2,304,548–5,463,607), while the incidence rate and EAPC were greatest in high SDI regions (2273.16 per 100,000, EAPC = 1.72, 95% CI, 1.38–2.07%; Table 2). DALYs mirrored these patterns: low-middle SDI regions had the greatest number of DALYs (427,282, 95% UI, 231,261–683,721), but high SDI regions had the highest DALY rate (259.34 per 100,000, 95% UI, 155.57–384.57; EAPC = 1.64, 95% CI, 1.31–1.98%; Table 3).

### Geographic regional trends

**Figure 4:**
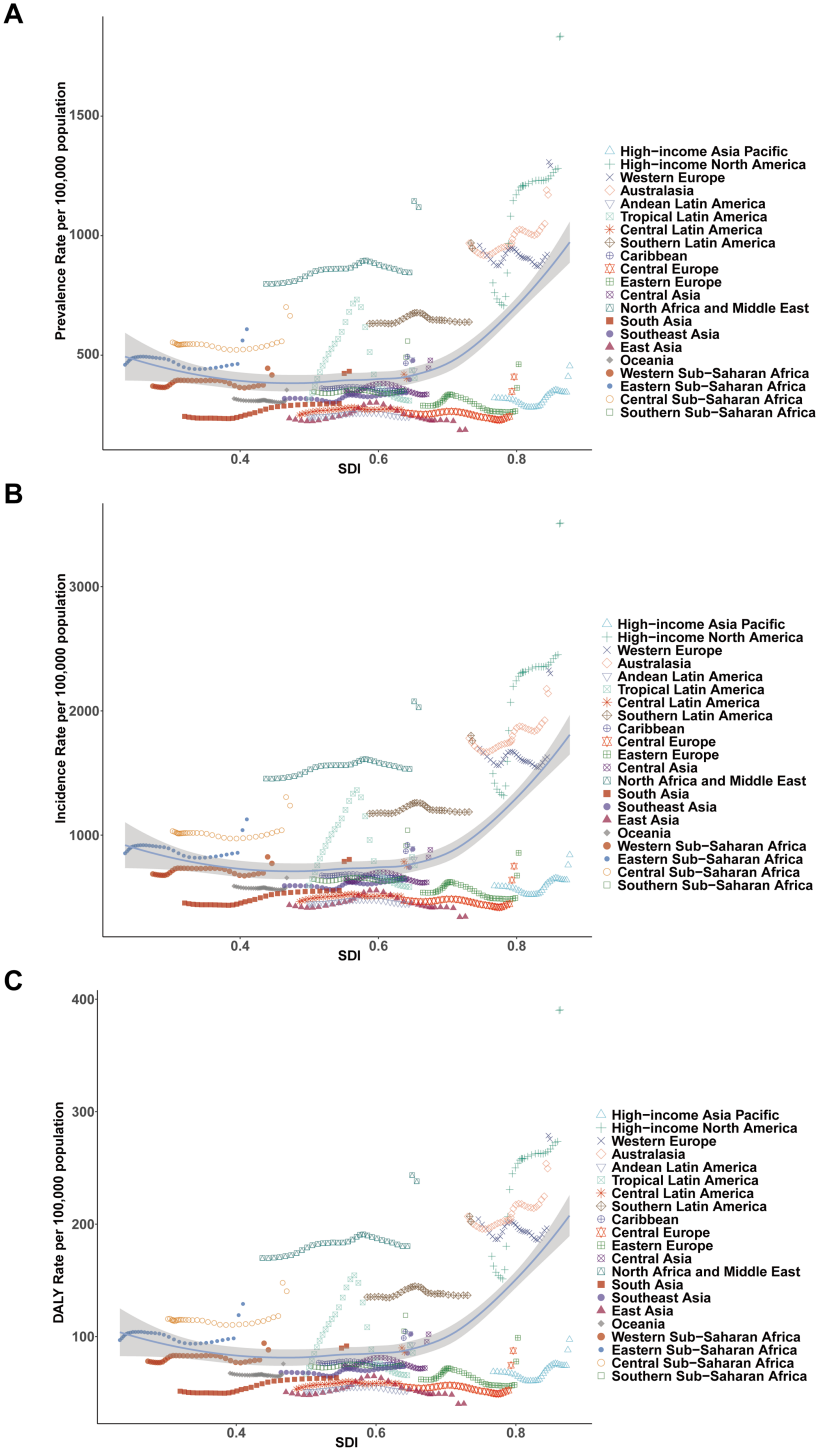
Association between prevalence, incidence, and disability-adjusted life years (DALYs) rates of childhood major depressive disorders and regional Socio-demographic Index (SDI), 1990–2021. **(A)** Prevalence rate. **(B)** Incidence rate. **(C)** DALY rate.

In 2021, the prevalence of childhood MDD was highest in high-income North America (1834.86 per 100,000, 95% UI, 1332.05–2425.99), followed by Western Europe (1293.16 per 100,000, 95% UI, 789.48–1842.68) and North Africa & the Middle East (1119.33 per 100,000, 95% UI, 683.61–1613.16). The lowest prevalence was recorded in East Asia (187.37 per 100,000, 95% UI, 116.85–268.68). A similar pattern was seen for incidence and DALYs (Tables 1–3 and Figures 4A–C).

### National trends

**Figure 5:**
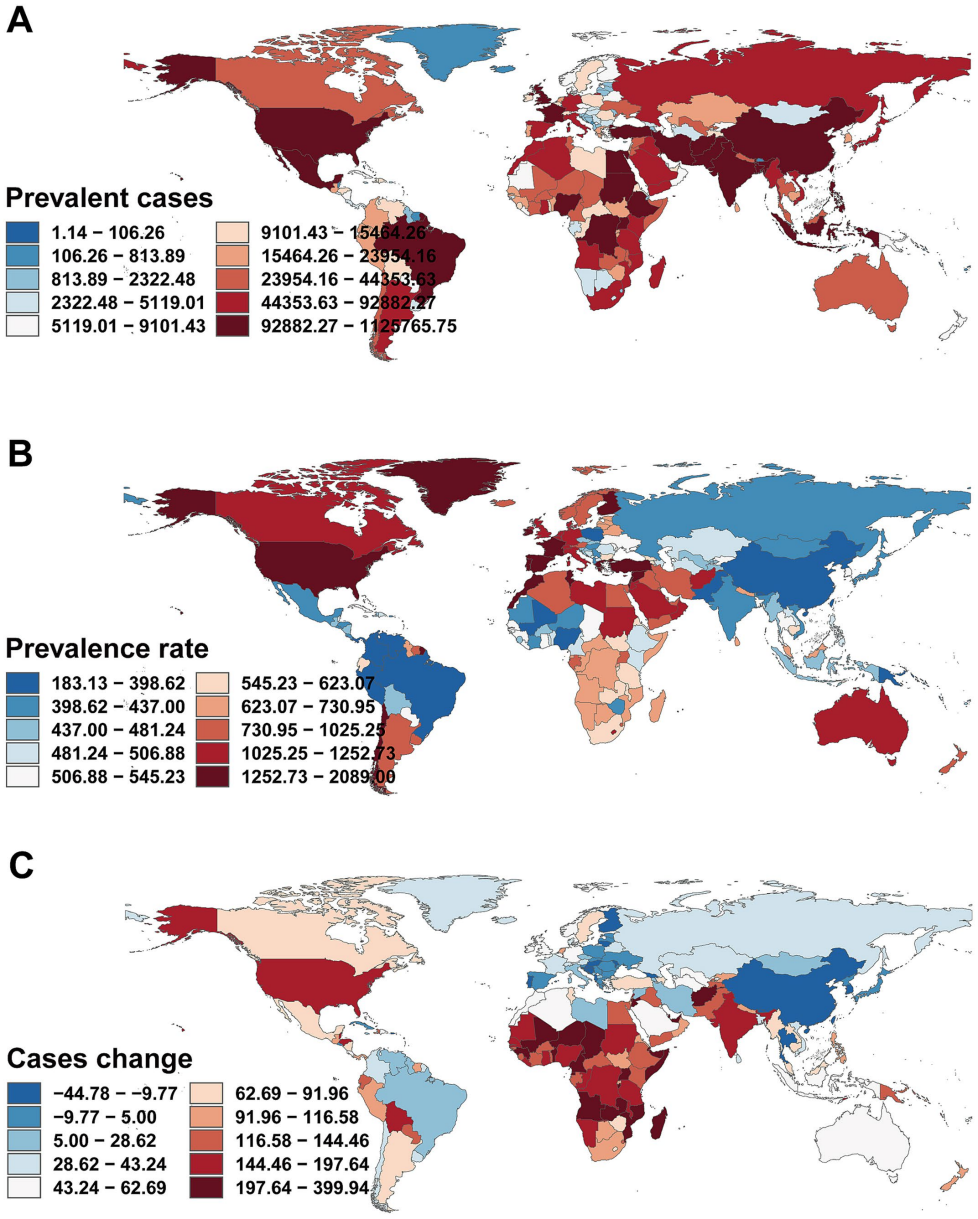
Prevalence of childhood major depressive disorders across 204 countries and territories. **(A)** Number of prevalent cases. **(B)** Prevalence rate. **(C)** Change in prevalent cases.

**Figure 6:**
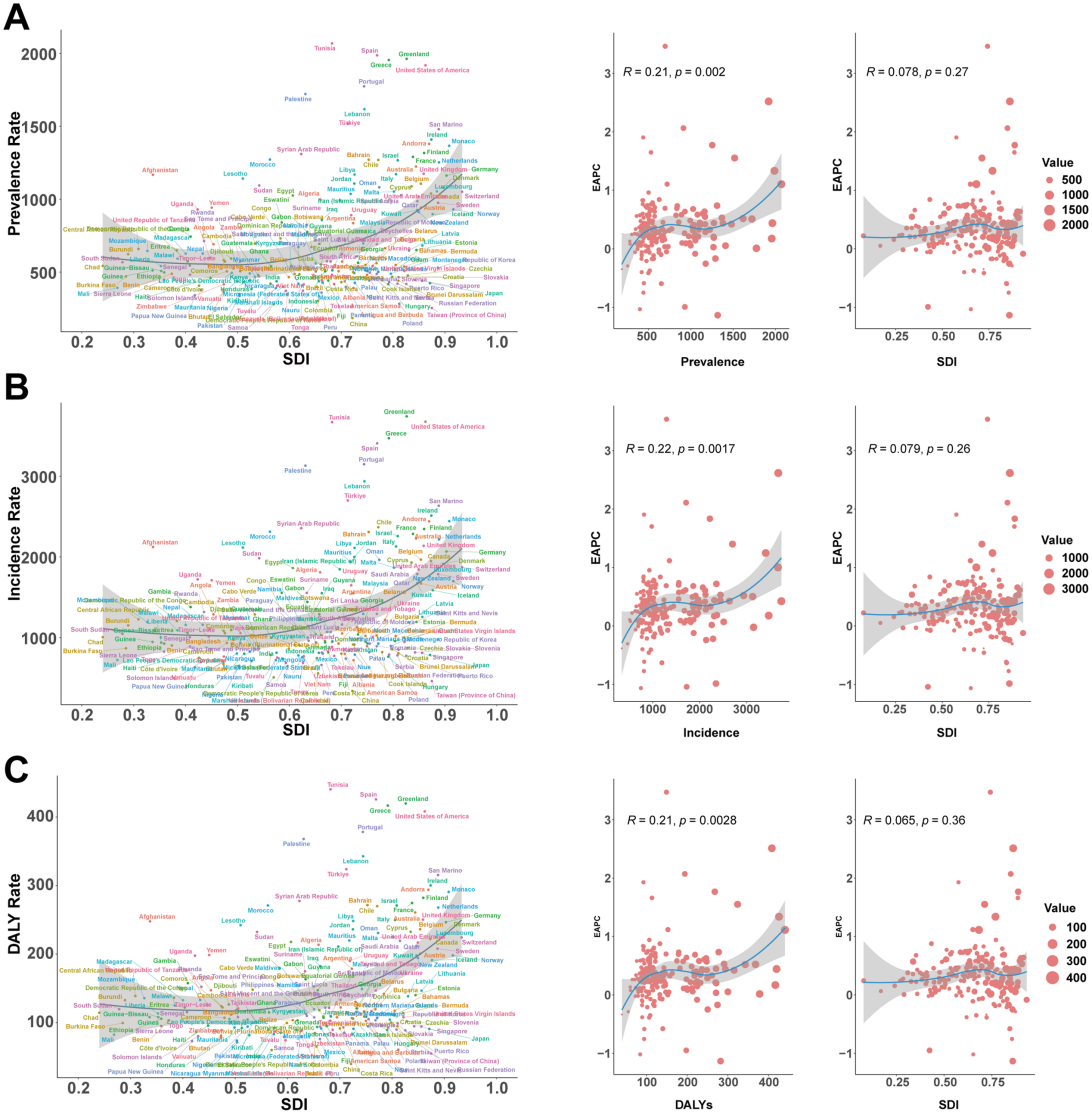
Prevalence, incidence and DALYs rates of major depressive disorders in children in 204 countries by SDI in 2021. **(A)** Prevalence rate. **(B)** Incidence rate. **(C)** DALY rate. DALYs, disability-adjusted life-years; SDI, Socio-demographic Index.

From 1990 to 2021, most countries experienced an increase in the number of MDD cases among children aged 5–14 years. Notably, Afghanistan [23,537 (95% UI, 14,033–34,593) to 101,793 (95% UI 58,612–156,193)], India [451,419 (95% UI 282,639–663,731) to 1,114,620 (95% UI 698,356–1,655,573)], and the United States [295,912 (95% UI 195,585–404,541) to 783,378 (95% UI 568,929–1,035,110)] all exhibited substantial rises. The prevalence rate in the United States nearly doubled [818.35 (95% UI 540.89–1118.76) to 1918.15 (95% UI 1393.06–2534.54) per 100,000], while China saw a modest decrease [234.92 (95% UI 150.09–334.78) to 184.98 (95% UI 115.54–264.55) per 100,000]. Qatar demonstrated the greatest proportional rise in prevalence (396, 95% UI, 280.06–519.77%; Supplementary Table S1 and Figures 5A–C). In 2021, 93 countries had prevalence rates above the global average (568.00 per 100,000), while 112 were below (Figure 6A). Similar trends were observed for incidence and DALYs (Figures 6B,C and Supplementary Tables S2, S3).

### Risk factor

**Figure 7:**
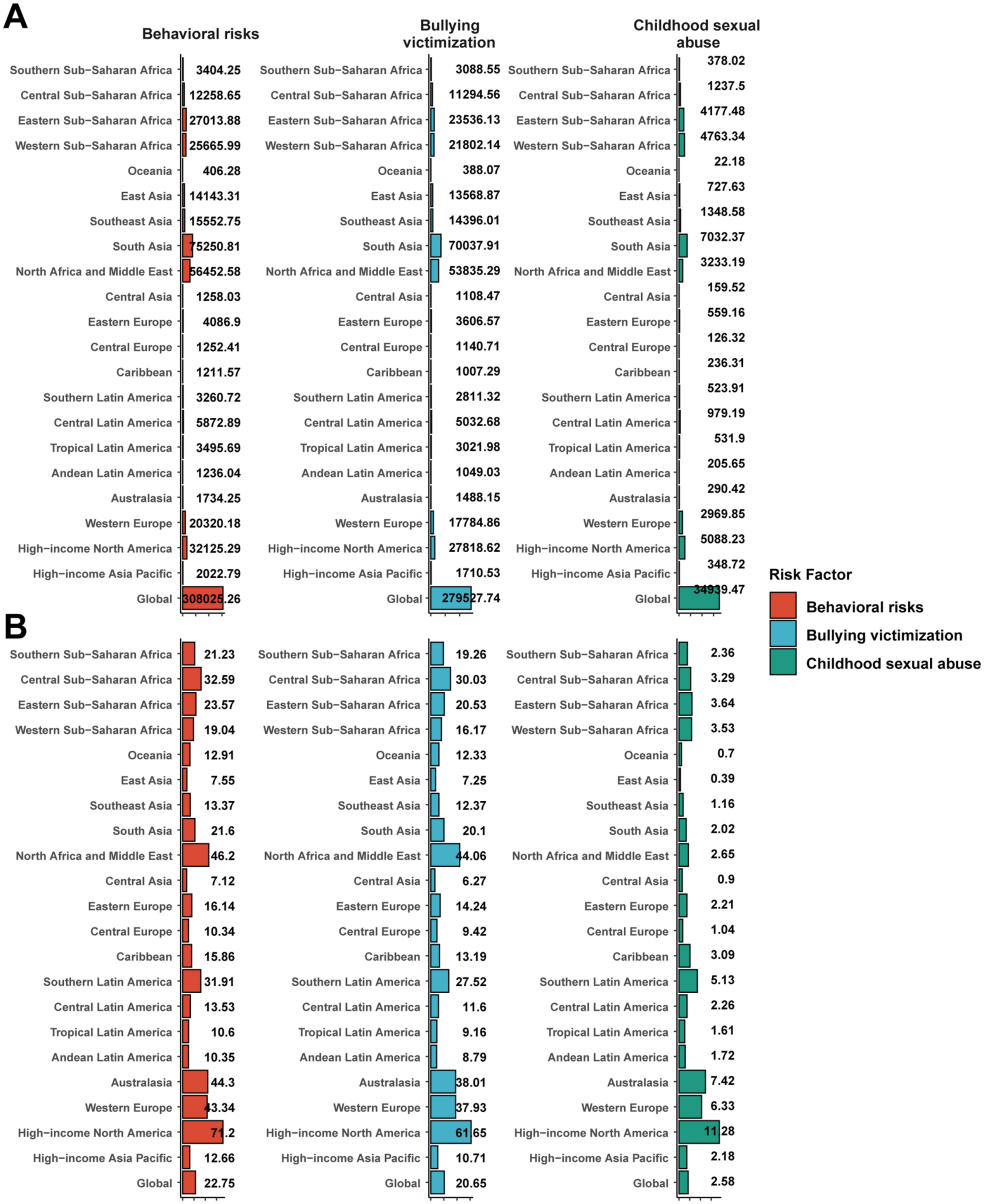
Proportion of childhood major depressive disorders DALYs attributable to risk factors. **(A)** Number of DALYs. **(B)** DALY rate. DALYs, disability-adjusted life-years.

Globally, bullying victimization emerged as the leading risk factor for DALYs due to childhood and adolescent MDD (total: 279,528), followed by behavioral risks (308,025) and childhood sexual abuse (34,940). South Asia reported the highest DALY numbers attributable to both bullying victimization and behavioral risks. On a per-population basis, behavioral risks [22.75 (95% UI, 9.94–41.09) per 100,000] and bullying victimization [20.65 (95% UI, 8.64–37.94) per 100,000] accounted for the largest shares of DALYs, while childhood sexual abuse contributed less [2.58 (95% UI, 1.07–4.80) per 100,000]. The highest behavioral risk-attributable DALY rate was observed in high-income North America [71.20 (95% UI 35.15–123.91) per 100,000; Figures 7A,B]. In the GBD framework, “behavioral risks” represent a broad aggregated category, whereas “bullying victimization” is treated as a specific, independent risk factor. Thus, although the total DALYs attributable to behavioral risks (308,025) are numerically higher, bullying victimization (279,528) was identified by the GBD data as the leading individual risk factor for childhood MDD.

### Projections to 2035

Using the ARIMA model, projections indicate a continued upward trajectory in the burden of childhood MDD through 2035. Between 2021 and 2035, the number of affected boys is expected to rise from 623,304 to 750,587 (mean annual increase 1.3%), while the number of affected girls is projected to increase from 1,012,163 to 1,214,951 (mean annual increase 1.4%). The persistent sex disparity is anticipated to remain, with female case counts consistently 1.6–1.8 times higher than those of males, although the growth patterns between the sexes are parallel. The prediction intervals broaden over time, reflecting greater uncertainty in long-range estimates, particularly for females (2035 95% UI: 906,157–1,523,745 for females vs. 566,187–934,987 for males).

**Figure 8:**
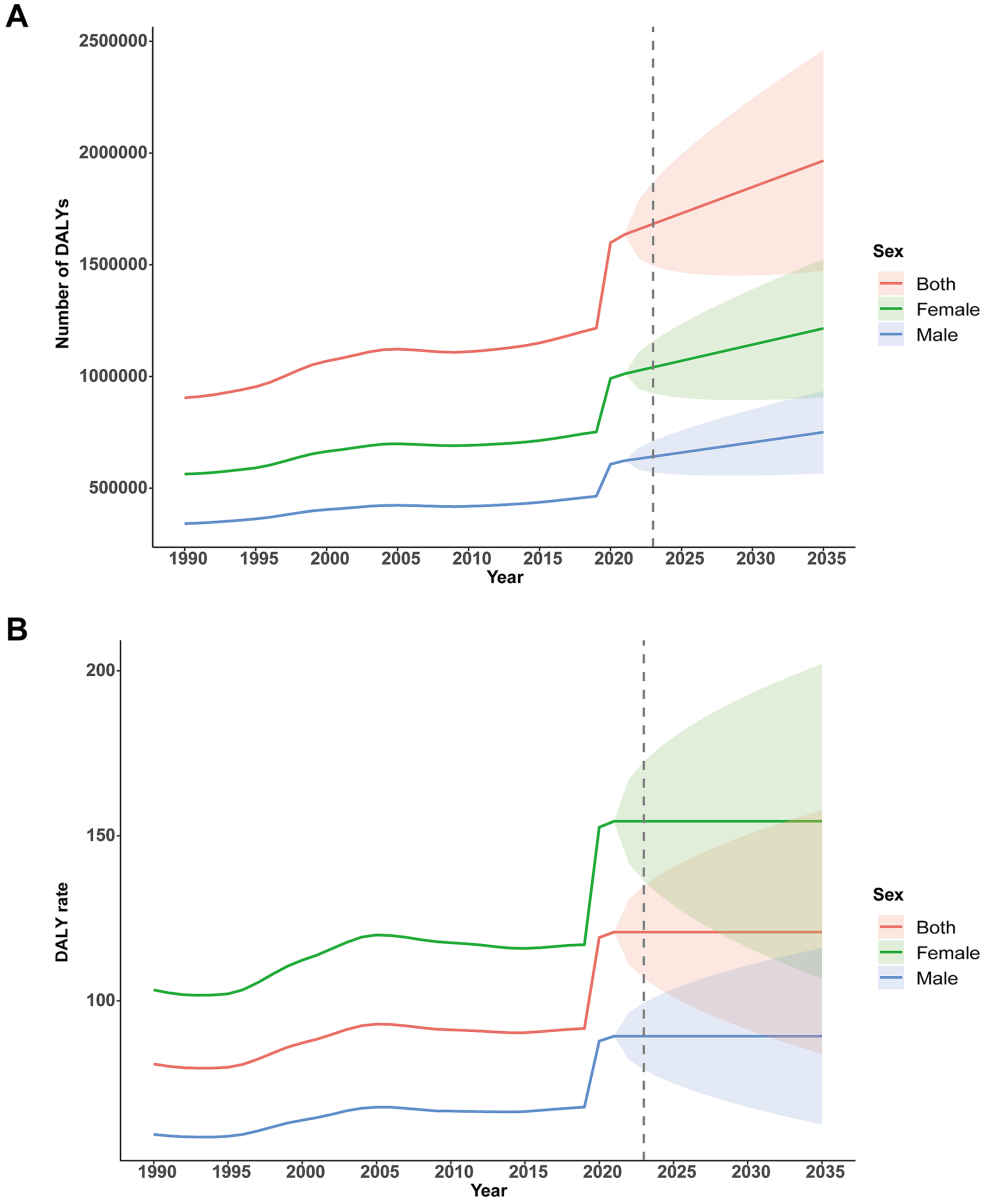
Forecasting the burden of DALYs up to 2035 using the ARIMA model. **(A)** Number of DALYs. **(B)** DALY rate. DALYs, disability-adjusted life-years.

ARIMA-based projections for DALY rates from 1990 to 2035 reveal a distinctly nonlinear temporal pattern. Among boys, DALY rates declined steadily until reaching a nadir in 1994, followed by a 16-year period of annual increases (1995–2010, mean annual growth: 0.55 units), stabilization from 2010 to 2020 (66.56 ± 0.42), and a notable spike in 2020 (+20 units). Post-2021, DALY rates for boys stabilize at 89.27 (95% CI, 67.84–111.87). In girls, overall DALY rates remained substantially higher throughout the period (*p* < 0.001), with greater fluctuations (from 103.28 to 152.59 between 1990 and 2020), stabilizing at 154.41 (95% CI, 116.19–192.63) after 2021. For both sexes combined, the uncertainty surrounding ARIMA projections narrows progressively over time, with the width of the 95% confidence interval in 2035 being 42.3% smaller than that in 2022, indicating enhanced confidence in long-term forecasts (Figure 8).

### The effect of PA on depressive symptoms

**Figure 9:**
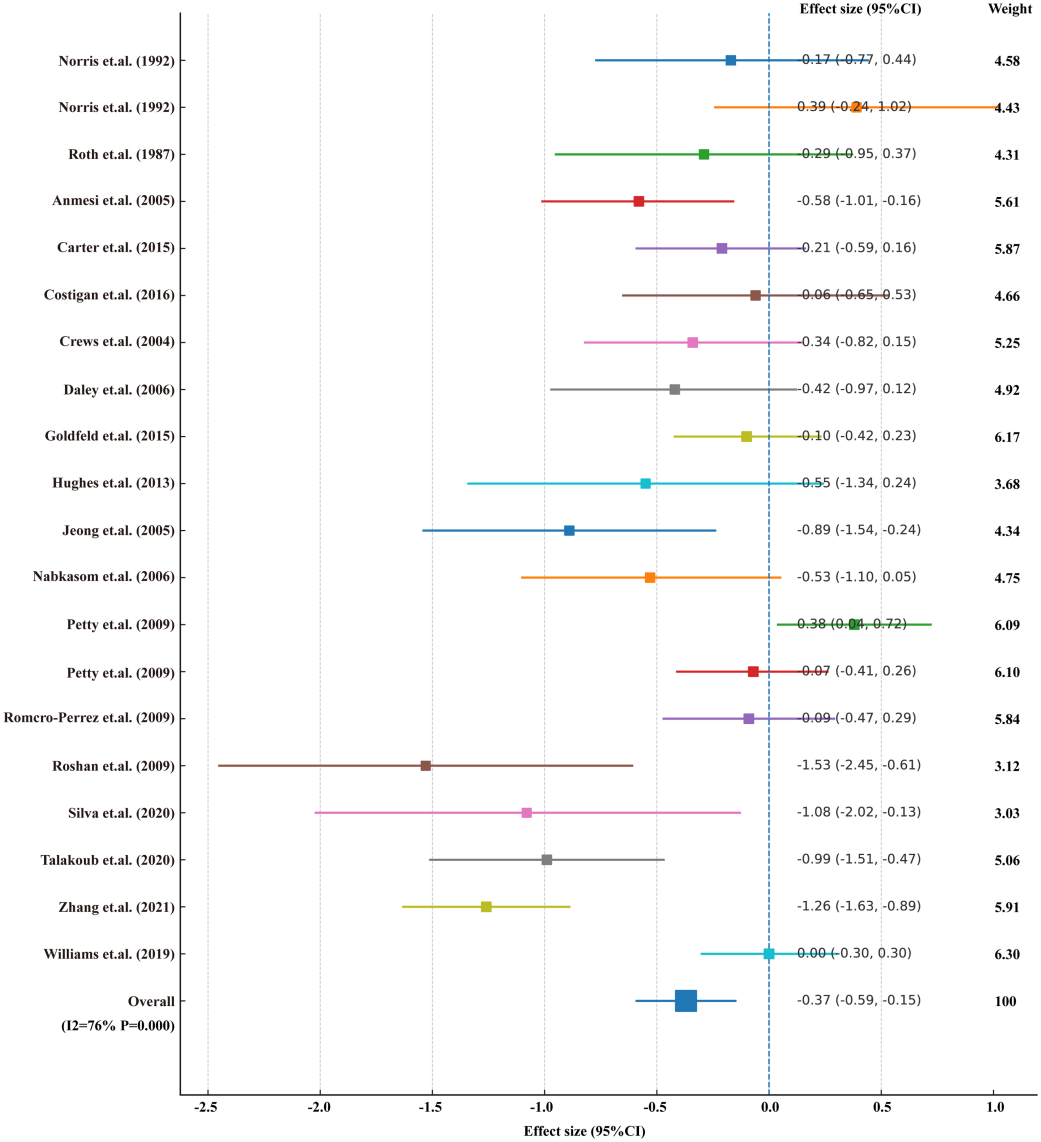
Meta-analysis of the effects of physical activity on depressive symptoms in children and adolescents.

The meta-analysis demonstrated that PA was associated with a significant reduction in depressive symptoms among children and adolescents (standardized mean difference −0.37, 95% CI −0.59 to −0.15; *I*^2^ = 76%; *p* < 0.001) (Figure 9).

## Discussion

The present analysis of GBD 2021 data indicates that MDD imposes a significant and growing burden on children aged 5–14 worldwide. We observed rising prevalence and incidence rates of pediatric MDD in most regions, mirroring broader trends seen in older youths (7). For instance, our estimates indicate a substantial absolute increase in MDD cases over the past three decades, which aligns with recent findings from an analysis reporting a persistent upward trend in DALYs attributable to mental disorders between 1990 and 2019 (17). As expected, we found higher burden in girls than boys: in nearly all regions female children had greater MDD prevalence and DALY rates, reflecting epidemiological data that rates of depressive disorders escalate sharply in adolescence and are consistently higher in females (18). These sex differences likely emerge during early puberty. Notably, in our analysis children ages 10–14 showed the largest pandemic-related increase in MDD cases (+31.7% above expected based on pre-2020 trends), underscoring that even pre-teens are a vulnerable subgroup.

Our findings highlight the critical role of psychosocial and behavioral risk factors in driving child MDD. Multinational analyses identify childhood adversities—especially bullying victimization and abuse—as major contributors to youth depression risk (19). In our focus group study, school bullying emerged as a key modifiable factor, with exposure to peer bullying substantially elevating the risk of subsequent MDD development. This underscores the need to tackle social stressors: for example, expanding anti-bullying initiatives and strengthening child protection can mitigate an important upstream cause. In parallel, lifestyle factors appear increasingly relevant (20–22). Systematic reviews find that physical inactivity and sedentary screen time are associated with poorer mental health in youth (23). Meta-analytic evidence suggests even modest amounts of daily screen use predict higher depression risk (pooled RR ≈ 1.10 for elevated screen time) (24). Conversely, regular exercise exerts a protective effect: observational and trial data show that physically active children have fewer depressive symptoms (25). Indeed, physical activity interventions—especially structured aerobic exercise three times weekly for ~40–50 min—significantly reduce depressive symptoms in children and adolescents (26). In short, insufficient physical activity remains a widespread behavioral risk factor, whereas sports participation and active play offer an accessible preventive approach.

Although the COVID-19 pandemic is not the focus of our analysis, its imprint provides important context. A Canadian study revealed that the prevalence of MDD in mental health surveys conducted during the COVID-19 pandemic was more than twice as high as that reported in the pre-pandemic Canadian Community Health Survey (27). Moreover, suicidal ideation was frequently observed among clinically stable patients with MDD during the COVID-19 pandemic (28). In our cohort, early adolescents (10–14 years) exhibited the most pronounced increase during the pandemic. These observations align with the notion that social isolation, disrupted schooling, and family stressors during COVID-19 have amplified underlying trends (29, 30). However, as some longitudinal surveys note, increases were heterogeneous and sometimes transient; nevertheless, the pandemic likely accelerated the rise in childhood depression (31). More broadly, our results confirm that even before COVID-19, child mental health was worsening, and that large-scale crises can sharply intensify this burden. These findings have several implications. First, they underscore that MDD is not uncommon even in primary school-age children and that symptoms often herald lifelong vulnerability. Early-onset depression can impair cognitive development, education, and socialization, and predispose to chronic mental illness in adulthood (32, 33). Our data reinforce the need for vigilance and screening in elementary and middle schools, not just high schools. Second, the evident inequalities demand equity-focused action. Although high-income countries currently show the heaviest burdens and largest increases, lower-SDI regions face growing challenges. Pediatric mental health services must be scaled up globally—for example, by integrating depression screening and counseling into primary care and school health programs, and by training lay counselors in resource-poor settings in evidence-based interventions. Third, the strong link between behavior and depression highlights prevention opportunities (34). Encouraging physical activity and limiting excessive sedentary screen use should be public health priorities (e.g., through daily PE, safe playgrounds, and active travel to school). Fourth, given that girls exhibit higher MDD rates even by age 10–14, interventions must be gender-sensitive. This includes addressing gender-based violence, promoting self-esteem and empowerment, and ensuring that female children have equal access to recreational activities and support services (35).

In light of these considerations, we propose several global public health and policy measures for the pediatric population:

- Comprehensive school-based programs: Governments and educators should implement WHO/UN-endorsed “whole school” mental health frameworks. Schools must not only identify and refer at-risk children, but actively promote emotional resilience. This includes anti-bullying policies and social–emotional learning curricula, strengthening teacher training in mental health first aid, and ensuring access to school counselors or psychologists. A policy review shows UN guidelines consistently recommend integrating mental health into broader school health initiatives. Effective implementation will require cross-sector investment (education, health, social services) and community engagement (36).
- Promotion of physical activity: National guidelines should mandate daily physical education and safe access to sports for all children. Public health campaigns can educate parents and schools about the mental health benefits of exercise. For example, after-school and community sports programs should be incentivized, and urban planning should include green spaces and pedestrian pathways to encourage active recreation. Empirical evidence shows that even short-term aerobic exercise programs yield significant reductions in youth depressive symptoms (37). Such lifestyle interventions are low-cost and confer broad health gains beyond mood (including obesity prevention and academic performance).
- Expansion of youth mental health services: Health systems must make evidence-based care accessible to children. This means scaling up cognitive-behavioral therapy (CBT) and other psychosocial interventions in pediatric settings. A meta-analysis found that CBT produces moderate improvements in youth depression and halved the risk of progression from subclinical symptoms to full MDD (38). Task-shifting models (training teachers, community health workers, and nurses in brief cognitive-behavioral strategies) can help close treatment gaps, especially in low- and middle-income countries. Digital therapies (online CBT, app-based mood monitoring) may also extend reach cost-effectively. Importantly, mental health services should be made youth-friendly and de-stigmatized (e.g., via confidential school clinics or telehealth).
- Addressing social determinants: Policymakers should tackle the root causes of pediatric depression. Anti-bullying laws, child abuse prevention programs, and poverty reduction measures are all investments in mental health. Since WHO explicitly recognizes violence, harsh parenting and socioeconomic stress as risk factors, interventions like parenting support programs, social welfare schemes, and community violence prevention will indirectly reduce depression onset. Gender equity must be strengthened (for instance, through school-based campaigns against gender discrimination), as evidence suggests this can buffer girls from despair (39).
- Ongoing surveillance and research: Finally, the global community should sustain rigorous monitoring of child MDD trends (via repeated GBD updates and population surveys) and evaluate interventions. As one analysis warns, mental health services utilization may surge following COVID-19, so countries must prepare (40). Research should also explore cultural factors, implementability of prevention programs, and potential novel approaches (e.g., school-based mindfulness, nutritional supplements) to expand the toolkit against pediatric depression.

In summary, MDD in children 5–14 is a pressing public health issue with substantial long-term consequences. Our findings—showing rising global burden and clear disparities by sex, age and region—underscore that prevention and treatment cannot be postponed. A concerted response involving education, healthcare, community and policy sectors is required. By promoting active lifestyles, safe psychosocial environments, and equitable access to care, stakeholders can help ensure that all children have the opportunity to thrive mentally and physically. Without such action, the burden of childhood depression—already one of the leading causes of disability among youth—is likely to grow further, undermining future generations’ health and well-being.

### Limitations

This study has several limitations. First, estimates of MDD burden rely on the quality and availability of underlying data within the GBD framework, which may be affected by underdiagnosis, misclassification, and reporting gaps, particularly in low- and middle-income countries. Second, cross-country comparability is limited by heterogeneity in diagnostic criteria, case ascertainment, and cultural perceptions of mental health. Third, the attribution of burden to specific behavioral or psychosocial risk factors is constrained by the availability of high-quality, age-specific exposure data and residual confounding. Fourth, while the study provides global and regional projections, uncertainties in long-term trends—especially given rapidly evolving social and environmental contexts—may affect forecast accuracy. Finally, the analysis focuses on ages 5–14, and caution is warranted when generalizing findings to older adolescents or other pediatric subgroups. Future research should address these gaps by strengthening surveillance systems, improving data harmonization, and expanding studies of risk and protective factors in diverse global settings.

## Conclusion

This comprehensive global analysis demonstrates that MDD in children aged 5–14 years is a growing contributor to morbidity and disability worldwide. The burden has risen sharply over the past three decades, particularly among girls and older children, and is further amplified by modifiable behavioral and psychosocial risk factors such as physical inactivity and bullying victimization. The COVID-19 pandemic has intensified these trends but is not their sole cause. Marked disparities in service access and prevention persist, especially in low-resource settings. Urgent, coordinated action is needed: school-based mental health promotion, anti-bullying strategies, universal physical activity programs, and equitable expansion of pediatric mental health services should be prioritized within national and international health agendas. Targeted, multisectoral interventions—grounded in robust surveillance and policy frameworks—are essential to curbing the childhood depression epidemic and safeguarding future generations’ mental health.

## Data Availability

The datasets presented in this study can be found in online repositories. The names of the repository/repositories and accession number(s) can be found at: https://vizhub.healthdata.org/gbd-results/.

## Footnotes

^1^ https://vizhub.healthdata.org/gbd-results/

## References

[1] Solmi M Radua J Olivola M Croce E Soardo L Salazar de Pablo G. Age at onset of mental disorders worldwide: large-scale meta-analysis of 192 epidemiological studies. Mol Psychiatry. (2022) 27:281–95. doi: 10.1038/s41380-021-01161-7, PMID: 34079068 PMC8960395

[2] World Health Organization (WHO) Mental health of adolescents. (2024). Available at: https://www.who.int/news-room/fact-sheets/detail/adolescent-mental-health#:~:text=Globally%2C%20it%20is%20estimated%20that,remain%20largely%20unrecognized%20and%20untreated (Accessed May 10, 2025).

[3] Baranne ML Falissard B. Global burden of mental disorders among children aged 5–14 years. Child Adolesc Psychiatry Ment Health. (2018) 12:19. doi: 10.1186/s13034-018-0225-4, PMID: 29682005 PMC5896103

[4] Clayborne ZM Varin M Colman I. Systematic review and meta-analysis: adolescent depression and long-term psychosocial outcomes. J Am Acad Child Adolesc Psychiatry. (2019) 58:72–9. doi: 10.1016/j.jaac.2018.07.896, PMID: 30577941

[5] Xie X Xue Q Zhou Y Zhu K Liu Q Zhang J. Mental health status among children in home confinement during the coronavirus disease 2019 outbreak in Hubei Province, China. JAMA Pediatr. (2020) 174:898–900. doi: 10.1001/jamapediatrics.2020.1619, PMID: 32329784 PMC7182958

[6] Racine N McArthur BA Cooke JE Eirich R Zhu J Madigan S. Global prevalence of depressive and anxiety symptoms in children and adolescents during COVID-19: a meta-analysis. JAMA Pediatr. (2021) 175:1142–50. doi: 10.1001/jamapediatrics.2021.2482, PMID: 34369987 PMC8353576

[7] Liu Y Ren Y Liu C Chen X Li D Peng J. Global burden of mental disorders in children and adolescents before and during the COVID-19 pandemic: evidence from the Global Burden of Disease Study 2021. Psychol Med. (2025) 55:e90. doi: 10.1017/s0033291725000649, PMID: 40098477 PMC12080646

[8] Essau CA Lewinsohn PM Seeley JR Sasagawa S. Gender differences in the developmental course of depression. J Affect Disord. (2010) 127:185–90. doi: 10.1016/j.jad.2010.05.016, PMID: 20573404 PMC3754427

[9] GBD 2021 Diseases and Injuries Collaborators. Global incidence, prevalence, years lived with disability (YLDs), disability-adjusted life-years (DALYs), and healthy life expectancy (HALE) for 371 diseases and injuries in 204 countries and territories and 811 subnational locations, 1990–2021: a systematic analysis for the Global Burden of Disease Study 2021. Lancet. (2024) 403:2133–61. doi: 10.1016/s0140-6736(24)00757-8 38642570 PMC11122111

[10] Wu Z Xia F Lin R. Global burden of cancer and associated risk factors in 204 countries and territories, 1980–2021: a systematic analysis for the GBD 2021. J Hematol Oncol. (2024) 17:119. doi: 10.1186/s13045-024-01640-8, PMID: 39614359 PMC11607901

[11] Shan S Wu J Cao J Feng Y Zhou J Luo Z. Global incidence and risk factors for glaucoma: a systematic review and meta-analysis of prospective studies. J Glob Health. (2024) 14:04252. doi: 10.7189/jogh.14.04252, PMID: 39513294 PMC11544525

[12] Fan Y Fan A Yang Z Fan D. Global burden of mental disorders in 204 countries and territories, 1990–2021: results from the Global Burden of Disease Study 2021. BMC Psychiatry. (2025) 25:486. doi: 10.1186/s12888-025-06932-y, PMID: 40375174 PMC12080068

[13] Schaffer AL Dobbins TA Pearson SA. Interrupted time series analysis using autoregressive integrated moving average (ARIMA) models: a guide for evaluating large-scale health interventions. BMC Med Res Methodol. (2021) 21:58. doi: 10.1186/s12874-021-01235-8, PMID: 33752604 PMC7986567

[14] GBD 2021 Risk Factors Collaborators. Global burden and strength of evidence for 88 risk factors in 204 countries and 811 subnational locations, 1990–2021: a systematic analysis for the Global Burden of Disease Study 2021. Lancet. (2024) 403:2162–203. doi: 10.1016/s0140-6736(24)00933-4, PMID: 38762324 PMC11120204

[15] Kim HJ Fay MP Feuer EJ Midthune DN. Permutation tests for joinpoint regression with applications to cancer rates. Stat Med. (2000) 19:335–51. doi: 10.1002/(sici)1097-0258(20000215)19:3<335::aid-sim336>3.0.co;2-z, PMID: 10649300

[16] Cao G Liu J Liu M. Global, regional, and national incidence and mortality of neonatal preterm birth, 1990–2019. JAMA Pediatr. (2022) 176:787–96. doi: 10.1001/jamapediatrics.2022.1622, PMID: 35639401 PMC9157382

[17] GBD 2019 Mental Disorders Collaborators. Global, regional, and national burden of 12 mental disorders in 204 countries and territories, 1990–2019: a systematic analysis for the Global Burden of Disease Study 2019. Lancet Psychiatry. (2022) 9:137–50. doi: 10.1016/s2215-0366(21)00395-3 35026139 PMC8776563

[18] Maitra M Mitsuhashi H Rahimian R Chawla A Yang J Fiori LM. Cell type specific transcriptomic differences in depression show similar patterns between males and females but implicate distinct cell types and genes. Nat Commun. (2023) 14:2912. doi: 10.1038/s41467-023-38530-5, PMID: 37217515 PMC10203145

[19] Zhao L Lou Y Tao Y Wang H Xu N. Global, regional and national burden of depressive disorders in adolescents and young adults, 1990–2021: systematic analysis of the Global Burden of Disease Study 2021. Front Public Health. (2025) 13:1599602. doi: 10.3389/fpubh.2025.1599602, PMID: 40567978 PMC12187749

[20] Aslamyar D Pilz LK von Gall C. Relationships between self-reported sleep quality, quantity and timing on workdays vs work-free days and lifestyle factors in healthy adults. Nat Sci Sleep. (2025) 17:1641–54. doi: 10.2147/nss.S537593 40688521 PMC12276750

[21] Kim J Kim T. Teen dating violence victimization and mental health in adulthood: the mediating roles of violence experiences and lifestyle factors. Soc Sci Med. (2025) 383:118423. doi: 10.1016/j.socscimed.2025.118423, PMID: 40684691

[22] Li Y Chen Y Zhao H Zhou W Lai W Hao J. Combined lifestyle, childhood trauma and depressive symptoms in adults with subthreshold depression: a prospective cohort study. Epidemiol Psychiatr Sci. (2025) 34:e39. doi: 10.1017/s2045796025100127, PMID: 40660782 PMC12281045

[23] Rodriguez-Ayllon M Cadenas-Sánchez C Estévez-López F Muñoz NE Mora-Gonzalez J Migueles JH. Role of physical activity and sedentary behavior in the mental health of preschoolers, children and adolescents: a systematic review and meta-analysis. Sports Med. (2019) 49:1383–410. doi: 10.1007/s40279-019-01099-5, PMID: 30993594

[24] Li L Zhang Q Zhu L Zeng G Huang H Zhuge J. Screen time and depression risk: a meta-analysis of cohort studies. Front Psychiatry. (2022) 13:1058572. doi: 10.3389/fpsyt.2022.1058572, PMID: 36620668 PMC9815119

[25] Biddle SJ Asare M. Physical activity and mental health in children and adolescents: a review of reviews. Br J Sports Med. (2011) 45:886–95. doi: 10.1136/bjsports-2011-090185, PMID: 21807669

[26] Li J Zhou X Huang Z Shao T. Effect of exercise intervention on depression in children and adolescents: a systematic review and network meta-analysis. J Affect Disord. (2023) 23:1918. doi: 10.1016/j.jad.2023.10.044 37832731

[27] Shields M Tonmyr L Gonzalez A Weeks M Park SB Robert AM. Symptoms of major depressive disorder during the COVID-19 pandemic: results from a representative sample of the Canadian population. Health Promot Chronic Dis Prev Can. (2021) 41:340–58. doi: 10.24095/hpcdp.41.11.04, PMID: 34569772 PMC8639172

[28] Zhang L Cai H Bai W Zou SY Feng KX Li YC. Prevalence of suicidality in clinically stable patients with major depressive disorder during the COVID-19 pandemic. J Affect Disord. (2022) 307:142–8. doi: 10.1016/j.jad.2022.03.042, PMID: 35337925 PMC8938301

[29] Vasileva M Alisic E Cobham VE Gash T Hoeboer CM Hoehn E. COVID-19 unmasked: trajectories, risk and protective factors for mental health outcomes in young Australian children during the first year of the COVID-19 pandemic. Aust J Psychol. (2025) 77:2519037. doi: 10.1080/00049530.2025.2519037, PMID: 40666212 PMC12218426

[30] Lee J. Mental health effects of school closures during COVID-19. Lancet Child Adolesc Health. (2020) 4:421. doi: 10.1016/s2352-4642(20)30109-7, PMID: 32302537 PMC7156240

[31] Hunter S Smith BT Schwartz N Rebellato S Patte KA Hilario C. The association between local public health unit funding and adolescent mental health during the COVID-19 pandemic: longitudinal findings from the Ontario public health information database (OPHID) and COMPASS studies. J Affect Disord. (2025) 390:119759. doi: 10.1016/j.jad.2025.119759, PMID: 40562113

[32] Wang M Wei J Dou Y Wang Y Fan H Yan Y. Differential association between childhood trauma subtypes and neurocognitive performance in adults with major depression. BMC Psychiatry. (2024) 24:773. doi: 10.1186/s12888-024-06226-9, PMID: 39506707 PMC11539613

[33] Goerigk S Elsaesser M Reinhard MA Kriston L Härter M Hautzinger M. Childhood trauma questionnaire-based child maltreatment profiles to predict efficacy of the cognitive behavioral analysis system of psychotherapy versus non-specific psychotherapy in adults with early-onset chronic depression: cluster analysis of data from a randomised controlled trial. Lancet Psychiatry. (2024) 11:709–19. doi: 10.1016/s2215-0366(24)00209-8 39147459

[34] Tsypes A Hallquist MN Ianni A Kaurin A Wright AGC Dombrovski AY. Exploration-exploitation and suicidal behavior in borderline personality disorder and depression. JAMA Psychiatry. (2024) 81:1010–9. doi: 10.1001/jamapsychiatry.2024.1796, PMID: 38985462 PMC11238070

[35] Deng WX Liu XB Guo T Shang LF Li Y Zeng K. Metabolomic changes in major depressive disorder adolescent females with or without suicide attempts. Curr Neuropharmacol. (2025) 23:787–99. doi: 10.2174/1570159x23666250122093451, PMID: 39844402 PMC12163494

[36] Margaretha M Azzopardi PS Fisher J Sawyer SM. School-based mental health promotion: a global policy review. Front Psychiatry. (2023) 14:1126767. doi: 10.3389/fpsyt.2023.1126767, PMID: 37139309 PMC10149729

[37] Imboden C Gerber M Beck J Holsboer-Trachsler E Pühse U Hatzinger M. Aerobic exercise or stretching as add-on to inpatient treatment of depression: similar antidepressant effects on depressive symptoms and larger effects on working memory for aerobic exercise alone. J Affect Disord. (2020) 276:866–76. doi: 10.1016/j.jad.2020.07.052, PMID: 32739704

[38] Oud M de Winter L Vermeulen-Smit E Bodden D Nauta M Stone L. Effectiveness of CBT for children and adolescents with depression: a systematic review and meta-regression analysis. Eur Psychiatry. (2019) 57:33–45. doi: 10.1016/j.eurpsy.2018.12.008, PMID: 30658278

[39] Stein C Flor LS Gil GF Khalil M Herbert M Aravkin AY. The health effects associated with physical, sexual and psychological gender-based violence against men and women: a Burden of Proof study. Nat Hum Behav. (2025) 9:1201–16. doi: 10.1038/s41562-025-02144-2, PMID: 40210704 PMC12185316

[40] Amro A Kotkot HA Albobali Y Chandra P Khan YS. Epidemic preparedness and innovations in digital healthcare: enhancing post-pandemic speech-language pathology services for child and adolescent mental health in Qatar. BMC Health Serv Res. (2024) 24:673. doi: 10.1186/s12913-024-10989-y, PMID: 38807136 PMC11134672

