# Supplementary material for: Major depressive disorders in children aged 5–14 years: a Global Burden of Disease analysis from the perspective of exercise psychology
Source: Front Public Health. 2025 Oct 29;13:1671222. doi: 10.3389/fpubh.2025.1671222 (PMC12605264; doi:10.3389/fpubh.2025.1671222)
Supplement: Supplementary file 1 [file Table_1.docx]

Table S1: Prevalence of major depressive disorders in children between 1990 and 2021 at the national level.

| location | 1990 | |  | 2021 | |  | 1990-2021 | |
| --- | --- | --- | --- | --- | --- | --- | --- | --- |
|  | Prevalent cases | Prevalence rate |  | Prevalent cases | Prevalence rate |  | Cases change | EAPC |
| Afghanistan | 23536.98(14032.57,34593.22) | 907.40(540.98,1333.64) |  | 101793.47(58612.43,156193.00) | 1167.47(672.22,1791.37) |  | 332.48(245.33,442.42) | 0.55(0.23,0.87) |
| Albania | 1778.28(1053.03,2682.18) | 249.24(147.59,375.93) |  | 1315.48(715.63,2073.41) | 436.92(237.69,688.66) |  | -26.03(-42.40,-6.23) | 0.83(0.48,1.18) |
| Algeria | 57925.25(34131.68,86542.51) | 828.85(488.39,1238.33) |  | 85845.38(48420.89,139105.02) | 998.55(563.23,1618.06) |  | 48.20(16.02,87.28) | 0.06(-0.20,0.31) |
| American Samoa | 33.95(19.85,51.37) | 297.78(174.12,450.52) |  | 40.90(22.14,64.88) | 390.51(211.44,619.50) |  | 20.45(-5.83,48.90) | 0.37(0.19,0.54) |
| Andorra | 62.22(37.40,93.41) | 915.78(550.44,1374.89) |  | 105.39(59.27,161.25) | 1379.22(775.64,2110.19) |  | 69.39(33.58,115.35) | 0.44(-0.07,0.94) |
| Angola | 15747.31(9445.58,24009.57) | 570.01(341.91,869.08) |  | 66426.08(37031.42,105420.95) | 691.05(385.25,1096.73) |  | 321.83(231.64,430.07) | 0.04(-0.23,0.31) |
| Antigua and Barbuda | 37.82(21.92,57.24) | 311.76(180.74,471.94) |  | 55.97(29.69,91.74) | 481.24(255.28,788.81) |  | 48.00(15.37,84.21) | 0.58(0.28,0.89) |
| Argentina | 35611.96(21593.28,53350.01) | 531.65(322.36,796.46) |  | 60039.16(34441.48,91393.75) | 835.37(479.21,1271.64) |  | 68.59(27.35,116.50) | 0.69(0.36,1.03) |
| Armenia | 2071.07(1206.40,3085.05) | 313.67(182.71,467.23) |  | 2322.48(1260.18,3694.57) | 571.82(310.27,909.64) |  | 12.14(-13.84,42.33) | 0.64(0.16,1.12) |
| Australia | 26518.36(16403.25,37468.19) | 1050.98(650.10,1484.95) |  | 39725.54(23511.07,60454.59) | 1223.84(724.31,1862.45) |  | 49.80(15.42,92.86) | 0.33(0.13,0.52) |
| Austria | 6200.00(3666.21,9131.54) | 687.88(406.76,1013.12) |  | 8145.06(4534.74,13019.80) | 941.73(524.30,1505.35) |  | 31.37(1.76,65.23) | 0.09(-0.24,0.42) |
| Azerbaijan | 4747.05(2776.83,6994.88) | 311.65(182.30,459.23) |  | 8295.36(4741.62,13298.55) | 505.96(289.20,811.12) |  | 74.75(37.41,117.93) | 0.50(0.09,0.91) |
| Bahamas | 174.86(98.91,268.09) | 317.01(179.33,486.05) |  | 329.89(180.33,549.18) | 548.65(299.91,913.36) |  | 88.67(45.81,135.90) | 0.79(0.35,1.23) |
| Bahrain | 908.98(540.95,1346.38) | 892.64(531.23,1322.18) |  | 2580.11(1427.47,4071.08) | 1270.85(703.11,2005.24) |  | 183.85(124.31,263.62) | 0.19(-0.10,0.48) |
| Bangladesh | 117777.14(70628.72,171892.86) | 392.85(235.58,573.35) |  | 164843.83(89917.36,259840.71) | 524.95(286.34,827.47) |  | 39.96(9.65,77.81) | 0.24(-0.09,0.56) |
| Barbados | 142.62(81.38,216.83) | 333.38(190.24,506.86) |  | 183.82(94.15,292.02) | 549.14(281.27,872.41) |  | 28.89(-1.68,62.96) | 0.48(0.10,0.86) |
| Belarus | 5701.48(3316.57,8663.16) | 356.76(207.53,542.08) |  | 7370.79(4130.17,11830.90) | 663.55(371.81,1065.06) |  | 29.28(-0.56,66.07) | 0.14(-0.40,0.68) |
| Belgium | 9426.25(6112.89,13747.12) | 779.27(505.35,1136.48) |  | 14604.79(8518.97,22896.15) | 1106.28(645.29,1734.33) |  | 54.94(15.90,103.84) | 0.45(0.06,0.84) |
| Belize | 163.10(94.40,250.84) | 311.75(180.44,479.45) |  | 414.24(228.53,677.15) | 487.43(268.91,796.79) |  | 153.97(99.99,224.67) | 0.75(0.44,1.06) |
| Benin | 5622.61(3346.44,8318.33) | 391.80(233.19,579.65) |  | 18266.29(9883.24,29149.36) | 488.66(264.40,779.80) |  | 224.87(158.50,313.52) | 0.37(0.23,0.52) |
| Bermuda | 28.21(15.91,43.40) | 369.97(208.59,569.08) |  | 31.65(16.81,51.08) | 537.68(285.66,867.92) |  | 12.17(-14.59,43.09) | 0.11(-0.24,0.47) |
| Bhutan | 565.15(331.90,846.51) | 338.97(199.07,507.72) |  | 461.04(256.97,742.59) | 365.51(203.72,588.72) |  | -18.42(-38.35,1.86) | 0.25(0.11,0.39) |
| Bolivia (Plurinational State of) | 4169.84(2400.86,6510.36) | 248.81(143.26,388.47) |  | 10674.42(5781.87,17521.67) | 465.66(252.23,764.36) |  | 155.99(96.53,236.95) | 0.73(0.11,1.34) |
| Bosnia and Herzegovina | 2673.66(1552.98,4079.07) | 359.37(208.74,548.27) |  | 1694.85(921.28,2729.40) | 499.35(271.44,804.17) |  | -36.61(-51.31,-17.28) | -0.41(-0.85,0.02) |
| Botswana | 1560.63(913.05,2380.15) | 413.50(241.92,630.63) |  | 3109.08(1659.44,4834.96) | 671.83(358.58,1044.77) |  | 99.22(55.78,159.00) | 0.33(0.01,0.65) |
| Brazil | 111713.67(69624.54,167289.21) | 314.84(196.22,471.47) |  | 126073.89(77263.44,190501.30) | 398.62(244.29,602.32) |  | 12.85(4.16,22.12) | -1.03(-2.12,0.07) |
| Brunei Darussalam | 185.29(108.96,273.39) | 330.32(194.25,487.39) |  | 281.38(155.84,465.23) | 441.71(244.63,730.31) |  | 51.86(17.61,90.92) | 0.53(0.33,0.74) |
| Bulgaria | 3795.16(2243.70,5812.59) | 316.42(187.07,484.62) |  | 3777.68(2042.70,6004.72) | 561.00(303.35,891.72) |  | -0.46(-23.82,28.91) | -0.17(-0.81,0.47) |
| Burkina Faso | 12041.08(7174.75,18164.28) | 423.60(252.41,639.02) |  | 29435.41(16459.16,47457.53) | 469.79(262.69,757.42) |  | 144.46(93.75,211.52) | 0.24(0.10,0.38) |
| Burundi | 7414.75(4459.53,11066.64) | 479.60(288.45,715.81) |  | 23014.85(12921.83,36527.38) | 623.07(349.83,988.89) |  | 210.39(139.38,296.73) | -0.02(-0.29,0.26) |
| Cabo Verde | 423.85(246.13,633.97) | 433.29(251.61,648.10) |  | 689.54(382.35,1111.50) | 695.51(385.66,1121.11) |  | 62.69(26.58,106.85) | 0.69(0.38,1.01) |
| Cambodia | 11049.90(6383.16,16538.78) | 389.64(225.08,583.19) |  | 19593.83(10808.03,32184.68) | 581.76(320.90,955.60) |  | 77.32(39.29,126.70) | 0.26(-0.01,0.54) |
| Cameroon | 12112.61(7128.54,18110.16) | 421.21(247.89,629.78) |  | 43465.71(23971.38,69056.49) | 505.23(278.63,802.68) |  | 258.85(182.93,347.48) | 0.22(0.06,0.37) |
| Canada | 24967.51(15499.26,36522.36) | 652.46(405.04,954.42) |  | 44353.63(24459.61,71870.48) | 1038.31(572.59,1682.47) |  | 77.65(33.99,130.47) | 0.41(0.07,0.75) |
| Central African Republic | 3932.75(2328.15,5891.67) | 546.99(323.81,819.45) |  | 9516.00(5234.50,15059.24) | 658.56(362.25,1042.18) |  | 141.97(85.26,206.09) | 0.36(0.14,0.57) |
| Chad | 8564.49(4974.52,13093.59) | 508.75(295.50,777.79) |  | 29173.99(15994.82,47249.20) | 542.33(297.34,878.34) |  | 240.64(168.00,331.44) | 0.16(0.01,0.31) |
| Chile | 23087.49(14151.76,34068.27) | 910.80(558.28,1343.99) |  | 32538.58(18747.12,50390.04) | 1269.11(731.20,1965.38) |  | 40.94(4.72,85.52) | 0.21(-0.18,0.60) |
| China | 485273.27(310049.78,691555.17) | 234.92(150.09,334.78) |  | 336592.98(210240.67,481375.93) | 184.98(115.54,264.55) |  | -30.64(-35.96,-24.39) | -0.35(-0.81,0.12) |
| Colombia | 16400.96(9606.06,25217.37) | 219.97(128.84,338.21) |  | 21693.47(11941.86,35503.52) | 302.55(166.55,495.15) |  | 32.27(2.18,72.31) | 0.25(-0.06,0.56) |
| Comoros | 545.23(323.73,821.03) | 418.52(248.50,630.23) |  | 909.62(518.95,1451.79) | 572.64(326.70,913.96) |  | 66.83(32.33,112.10) | 0.37(0.11,0.63) |
| Congo | 3838.90(2231.23,5910.39) | 583.00(338.85,897.58) |  | 9476.29(4998.47,14912.02) | 730.95(385.56,1150.24) |  | 146.85(88.61,213.19) | 0.04(-0.24,0.32) |
| Cook Islands | 14.64(8.44,22.55) | 336.22(193.83,517.77) |  | 11.81(6.33,19.19) | 443.47(237.81,720.51) |  | -19.34(-36.20,2.17) | 0.45(0.25,0.64) |
| Costa Rica | 1948.63(1116.90,2925.22) | 271.31(155.51,407.29) |  | 3057.07(1693.99,4915.63) | 431.18(238.93,693.33) |  | 56.88(24.81,99.78) | 0.60(0.29,0.91) |
| Croatia | 2259.80(1312.58,3454.05) | 329.54(191.41,503.70) |  | 1938.90(1063.96,3147.87) | 467.33(256.44,758.72) |  | -14.20(-33.54,9.67) | -0.04(-0.43,0.35) |
| Cuba | 6654.63(3770.31,10229.44) | 413.60(234.33,635.78) |  | 6339.97(3584.00,10165.31) | 514.38(290.78,824.74) |  | -4.73(-26.04,22.64) | -0.37(-0.73,-0.02) |
| Cyprus | 1091.20(657.50,1640.56) | 814.12(490.55,1223.99) |  | 1563.04(877.74,2399.65) | 1088.04(611.00,1670.40) |  | 43.24(12.83,79.64) | 0.21(-0.04,0.46) |
| Czechia | 5177.24(2976.95,7904.50) | 332.97(191.46,508.37) |  | 5497.78(2977.46,8951.83) | 476.30(257.95,775.53) |  | 6.19(-16.86,36.94) | -0.21(-0.70,0.29) |
| C么te d'Ivoire | 12349.88(7315.82,18587.76) | 364.00(215.62,547.85) |  | 29353.00(16591.87,47058.65) | 406.70(229.89,652.02) |  | 137.68(87.38,195.74) | 0.08(-0.07,0.22) |
| Democratic People's Republic of Korea | 9212.70(5541.88,13966.29) | 254.99(153.39,386.56) |  | 9101.43(5103.80,14306.46) | 279.13(156.53,438.77) |  | -1.21(-20.85,25.24) | 0.13(0.02,0.24) |
| Democratic Republic of the Congo | 56957.71(33456.75,86406.75) | 547.24(321.45,830.18) |  | 158460.17(85917.59,253044.39) | 648.41(351.57,1035.45) |  | 178.21(113.29,250.38) | 0.23(0.02,0.45) |
| Denmark | 4931.34(2946.04,7416.87) | 830.55(496.18,1249.17) |  | 6942.93(4105.35,10696.73) | 1078.99(638.00,1662.35) |  | 40.79(8.55,81.25) | 0.59(0.28,0.90) |
| Djibouti | 517.55(302.43,783.22) | 472.72(276.23,715.37) |  | 1585.03(869.23,2453.42) | 592.59(324.98,917.26) |  | 206.25(146.34,278.39) | 0.11(-0.14,0.37) |
| Dominica | 51.15(29.19,78.18) | 316.64(180.74,483.98) |  | 52.16(28.60,84.23) | 511.16(280.22,825.38) |  | 1.98(-19.99,28.12) | 0.57(0.23,0.91) |
| Dominican Republic | 6341.31(3607.52,9564.03) | 373.10(212.26,562.72) |  | 9226.06(4977.79,15022.92) | 484.23(261.26,788.47) |  | 45.49(14.45,85.33) | 0.41(0.12,0.70) |
| Ecuador | 8259.09(4706.55,12698.49) | 327.78(186.79,503.96) |  | 18842.12(10410.09,29918.27) | 552.59(305.30,877.43) |  | 128.14(73.79,195.01) | 0.76(0.29,1.24) |
| Egypt | 101950.52(60112.96,152422.00) | 746.95(440.42,1116.74) |  | 244190.95(138452.37,368936.42) | 1025.25(581.30,1549.00) |  | 139.52(85.31,201.58) | 0.31(0.03,0.59) |
| El Salvador | 4982.87(2940.52,7626.06) | 359.00(211.85,549.43) |  | 5119.01(2801.36,8205.74) | 420.26(229.98,673.67) |  | 2.73(-20.26,34.93) | -0.06(-0.38,0.26) |
| Equatorial Guinea | 640.74(380.12,982.72) | 558.84(331.53,857.11) |  | 2756.34(1550.63,4333.36) | 693.14(389.94,1089.71) |  | 330.18(237.96,441.40) | 0.29(0.06,0.53) |
| Eritrea | 4584.57(2739.76,6833.21) | 473.42(282.92,705.63) |  | 9207.33(5177.38,14991.52) | 573.10(322.26,933.13) |  | 100.83(56.13,152.83) | 0.16(0.01,0.32) |
| Estonia | 1005.79(608.83,1492.99) | 440.17(266.45,653.39) |  | 956.92(546.01,1503.15) | 651.16(371.54,1022.85) |  | -4.86(-26.49,24.47) | -0.60(-1.12,-0.07) |
| Eswatini | 966.63(556.08,1462.18) | 402.22(231.39,608.42) |  | 2286.72(1243.90,3646.62) | 840.03(456.95,1339.59) |  | 136.57(80.84,207.09) | 0.73(0.25,1.22) |
| Ethiopia | 59822.35(37652.89,84800.50) | 406.28(255.71,575.91) |  | 141173.87(88487.92,213063.77) | 497.35(311.74,750.61) |  | 135.99(108.64,166.28) | -0.01(-0.33,0.30) |
| Fiji | 581.33(344.20,889.53) | 310.54(183.87,475.18) |  | 802.71(437.42,1288.23) | 442.31(241.03,709.85) |  | 38.08(8.83,76.67) | 0.18(-0.07,0.44) |
| Finland | 8819.80(5457.81,12882.72) | 1352.18(836.74,1975.07) |  | 7957.84(4642.53,12511.43) | 1316.89(768.26,2070.43) |  | -9.77(-30.33,16.83) | -1.13(-1.65,-0.62) |
| France | 78724.57(51911.13,111931.03) | 1006.09(663.42,1430.46) |  | 104153.51(59829.55,159761.29) | 1290.03(741.04,1978.78) |  | 32.30(-2.40,70.31) | -0.17(-0.61,0.28) |
| Gabon | 1442.28(855.02,2137.39) | 573.90(340.22,850.49) |  | 3296.44(1835.05,5230.31) | 774.54(431.17,1228.92) |  | 128.56(83.38,187.34) | 0.34(0.10,0.58) |
| Gambia | 1600.88(943.61,2392.54) | 580.91(342.41,868.18) |  | 4726.09(2637.37,7423.88) | 743.26(414.77,1167.53) |  | 195.22(127.70,273.83) | 0.32(0.10,0.54) |
| Georgia | 3173.53(1859.20,4747.65) | 352.56(206.54,527.43) |  | 2777.72(1538.69,4497.87) | 563.84(312.33,913.01) |  | -12.47(-31.72,12.95) | 0.36(-0.01,0.73) |
| Germany | 62024.95(40232.29,89003.87) | 732.58(475.19,1051.23) |  | 92035.57(52193.69,148356.00) | 1161.89(658.91,1872.91) |  | 48.38(7.96,99.17) | 0.64(-0.03,1.31) |
| Ghana | 17674.73(10388.06,26466.18) | 432.93(254.45,648.27) |  | 44550.56(25094.58,73226.88) | 540.47(304.44,888.36) |  | 152.06(96.73,216.36) | 0.31(0.12,0.50) |
| Greece | 18602.61(11054.55,27974.32) | 1266.69(752.73,1904.83) |  | 19003.04(10970.18,29984.25) | 1954.77(1128.46,3084.36) |  | 2.15(-19.59,28.79) | 0.18(-0.21,0.57) |
| Greenland | 110.41(69.58,162.53) | 1266.69(798.30,1864.59) |  | 151.31(86.84,238.15) | 1962.38(1126.27,3088.70) |  | 37.04(3.95,78.03) | 0.44(0.10,0.78) |
| Grenada | 67.33(38.40,102.53) | 314.62(179.43,479.15) |  | 75.59(41.10,122.33) | 505.25(274.68,817.62) |  | 12.28(-11.88,42.03) | 0.58(0.28,0.87) |
| Guam | 86.08(50.86,131.90) | 337.17(199.20,516.62) |  | 122.73(68.97,194.79) | 515.72(289.79,818.51) |  | 42.58(12.54,78.18) | 0.68(0.41,0.96) |
| Guatemala | 7709.28(4438.18,11612.14) | 305.05(175.62,459.49) |  | 16157.24(9100.42,26228.45) | 478.77(269.66,777.19) |  | 109.58(64.00,165.78) | 0.70(0.39,1.02) |
| Guinea | 6351.73(3701.47,9470.28) | 399.15(232.61,595.13) |  | 19419.98(10665.26,30239.77) | 511.69(281.01,796.77) |  | 205.74(136.22,297.95) | 0.36(0.16,0.56) |
| Guinea-Bissau | 1215.98(709.14,1833.78) | 413.15(240.94,623.05) |  | 2868.70(1646.99,4548.75) | 507.29(291.25,804.39) |  | 135.92(85.57,196.05) | 0.21(0.00,0.41) |
| Guyana | 777.30(462.19,1194.94) | 429.13(255.16,659.71) |  | 999.43(546.73,1626.09) | 719.66(393.69,1170.90) |  | 28.58(-0.08,65.37) | 1.11(0.77,1.45) |
| Haiti | 5470.61(3166.13,8221.39) | 331.07(191.61,497.55) |  | 12783.81(7056.77,20165.57) | 459.37(253.57,724.62) |  | 133.68(86.22,192.24) | 0.23(-0.03,0.49) |
| Honduras | 3423.86(1966.62,5132.29) | 246.96(141.85,370.18) |  | 9422.08(4988.87,15187.32) | 431.88(228.67,696.14) |  | 175.19(114.43,250.20) | 0.76(0.35,1.18) |
| Hungary | 4898.62(2874.54,7527.46) | 324.01(190.13,497.88) |  | 3895.69(2155.15,6227.15) | 417.05(230.72,666.65) |  | -20.47(-39.09,-0.15) | -0.17(-0.54,0.20) |
| Iceland | 315.33(186.64,470.50) | 745.31(441.12,1112.06) |  | 408.89(232.59,640.70) | 897.38(510.46,1406.14) |  | 29.67(1.22,67.24) | 0.10(-0.15,0.35) |
| India | 451419.21(282638.67,663730.65) | 214.68(134.41,315.65) |  | 1114619.55(698356.02,1655573.37) | 437.00(273.80,649.08) |  | 146.91(131.66,163.22) | 1.90(1.54,2.27) |
| Indonesia | 125487.16(79876.69,177727.12) | 276.30(175.88,391.33) |  | 198328.75(124795.07,286245.28) | 437.02(274.99,630.75) |  | 58.05(47.91,68.98) | 0.41(0.03,0.79) |
| Iran (Islamic Republic of) | 108221.42(68037.78,151937.89) | 651.45(409.56,914.60) |  | 139193.36(87926.20,199110.64) | 992.52(626.96,1419.76) |  | 28.62(20.72,36.80) | 0.48(0.15,0.82) |
| Iraq | 35302.33(20518.53,52312.48) | 693.06(402.82,1027.00) |  | 78624.89(45116.21,123185.05) | 857.56(492.08,1343.57) |  | 122.72(75.90,182.83) | 0.46(-0.02,0.93) |
| Ireland | 6520.63(3843.26,9530.14) | 940.21(554.16,1374.15) |  | 9847.12(5602.02,15818.49) | 1409.15(801.67,2263.67) |  | 51.01(18.28,92.76) | 0.05(-0.37,0.48) |
| Israel | 10459.29(6247.95,15476.56) | 1028.75(614.53,1522.24) |  | 21657.27(12593.41,33805.53) | 1266.90(736.69,1977.55) |  | 107.06(61.55,163.63) | 0.03(-0.28,0.34) |
| Italy | 52087.04(33732.54,71549.16) | 803.31(520.24,1103.47) |  | 63622.77(41309.33,90292.93) | 1171.74(760.80,1662.93) |  | 22.15(13.46,31.04) | 0.29(-0.20,0.78) |
| Jamaica | 1764.98(1023.54,2680.38) | 317.33(184.03,481.91) |  | 2119.27(1124.71,3378.34) | 513.45(272.49,818.50) |  | 20.07(-6.87,56.14) | 0.64(0.32,0.95) |
| Japan | 50047.68(31592.23,72160.10) | 304.60(192.28,439.18) |  | 46066.96(29002.65,66176.67) | 424.23(267.09,609.42) |  | -7.95(-13.38,-2.34) | 0.36(0.03,0.69) |
| Jordan | 8674.17(5102.04,12831.02) | 839.56(493.82,1241.90) |  | 28080.62(15829.32,44659.17) | 1107.40(624.25,1761.20) |  | 223.73(158.62,303.66) | 0.40(0.18,0.63) |
| Kazakhstan | 12498.49(7667.29,18131.76) | 377.22(231.41,547.24) |  | 17182.53(9464.33,26655.43) | 494.03(272.12,766.39) |  | 37.48(4.48,74.57) | 0.03(-0.33,0.40) |
| Kenya | 26201.39(16944.01,37832.55) | 380.99(246.38,550.12) |  | 62384.99(40082.85,90402.83) | 490.64(315.24,710.99) |  | 138.10(127.94,147.60) | 0.26(-0.02,0.54) |
| Kiribati | 54.54(32.08,81.97) | 307.14(180.68,461.64) |  | 106.26(59.70,173.00) | 383.28(215.34,624.02) |  | 94.84(49.09,148.47) | 0.08(-0.10,0.26) |
| Kuwait | 2892.58(1711.42,4360.50) | 826.70(489.12,1246.23) |  | 5375.32(2956.40,8514.93) | 926.02(509.31,1466.90) |  | 85.83(43.81,135.77) | 0.27(0.06,0.48) |
| Kyrgyzstan | 3926.04(2416.56,5870.50) | 379.39(233.52,567.29) |  | 8045.85(4594.13,12564.43) | 543.46(310.31,848.67) |  | 104.94(58.10,156.49) | 0.22(-0.14,0.59) |
| Lao People's Democratic Republic | 4006.32(2327.39,6105.82) | 353.87(205.57,539.31) |  | 7252.63(3946.52,11555.15) | 494.44(269.05,787.76) |  | 81.03(41.04,129.51) | 0.55(0.33,0.77) |
| Latvia | 1359.60(818.57,2014.50) | 371.15(223.46,549.93) |  | 1318.38(716.93,2131.99) | 648.35(352.57,1048.47) |  | -3.03(-25.87,26.30) | -0.10(-0.65,0.45) |
| Lebanon | 5406.51(3251.30,7896.45) | 823.83(495.43,1203.24) |  | 14103.36(7931.31,22166.79) | 1617.07(909.39,2541.60) |  | 160.86(99.16,238.61) | 0.52(0.10,0.94) |
| Lesotho | 2792.46(1619.19,4261.82) | 640.05(371.13,976.84) |  | 4871.55(2729.53,7745.31) | 1141.48(639.57,1814.85) |  | 74.45(33.15,128.06) | 0.60(0.17,1.04) |
| Liberia | 2939.74(1752.47,4355.77) | 441.57(263.23,654.26) |  | 7684.72(4195.64,12531.06) | 541.35(295.56,882.75) |  | 161.41(106.12,234.23) | 0.66(0.42,0.91) |
| Libya | 10407.92(6300.65,15282.39) | 886.52(536.68,1301.72) |  | 12487.58(7068.94,19526.65) | 1168.65(661.55,1827.41) |  | 19.98(-5.24,48.73) | 0.42(0.15,0.69) |
| Lithuania | 2094.68(1254.23,3133.07) | 386.74(231.57,578.46) |  | 1885.25(1054.73,3026.39) | 682.58(381.88,1095.74) |  | -10.00(-31.12,16.89) | 0.19(-0.30,0.68) |
| Luxembourg | 352.88(212.12,525.77) | 817.33(491.30,1217.77) |  | 718.33(417.24,1169.61) | 1053.81(612.10,1715.85) |  | 103.56(57.92,160.23) | 0.14(-0.20,0.49) |
| Madagascar | 15598.43(9268.22,23340.82) | 472.63(280.82,707.22) |  | 47889.55(25676.97,75495.36) | 626.38(335.85,987.45) |  | 207.02(134.84,285.71) | 0.30(0.03,0.56) |
| Malawi | 10841.41(6488.57,16102.47) | 408.97(244.77,607.44) |  | 31978.46(17897.77,50895.76) | 592.21(331.45,942.55) |  | 194.97(127.85,275.35) | 0.30(-0.02,0.63) |
| Malaysia | 18888.85(11067.51,28463.77) | 450.74(264.10,679.22) |  | 35905.84(19575.15,57890.77) | 696.49(379.71,1122.95) |  | 90.09(46.25,140.60) | 3.46(2.41,4.53) |
| Maldives | 266.71(157.63,409.75) | 421.61(249.18,647.72) |  | 382.94(208.79,626.11) | 559.76(305.21,915.22) |  | 43.58(11.86,83.51) | -0.11(-0.42,0.20) |
| Mali | 7978.57(4731.74,11759.23) | 332.35(197.10,489.84) |  | 27443.12(15259.78,43746.72) | 392.30(218.14,625.36) |  | 243.96(170.86,333.10) | 0.23(0.02,0.45) |
| Malta | 497.37(292.57,746.28) | 839.05(493.56,1258.97) |  | 441.99(250.43,692.39) | 1052.08(596.11,1648.10) |  | -11.13(-30.21,12.76) | 0.24(-0.00,0.48) |
| Marshall Islands | 43.83(25.81,65.79) | 302.14(177.96,453.52) |  | 46.02(24.96,75.58) | 390.51(211.77,641.38) |  | 5.00(-19.22,34.18) | 0.12(-0.07,0.31) |
| Mauritania | 1970.63(1152.73,2951.17) | 356.09(208.29,533.27) |  | 5216.58(2851.10,8396.84) | 436.16(238.38,702.07) |  | 164.72(106.72,244.13) | 0.24(-0.00,0.48) |
| Mauritius | 1685.79(983.44,2590.54) | 750.63(437.89,1153.47) |  | 1482.90(814.33,2358.01) | 1036.21(569.03,1647.71) |  | -12.04(-32.94,12.27) | 0.21(-0.17,0.58) |
| Mexico | 51032.47(32419.64,74604.10) | 236.06(149.96,345.10) |  | 94918.33(59941.36,140320.97) | 427.76(270.13,632.37) |  | 86.00(73.54,98.18) | 0.83(0.41,1.26) |
| Micronesia (Federated States of) | 95.61(55.76,143.85) | 315.87(184.24,475.28) |  | 84.38(48.21,136.73) | 399.31(228.17,647.06) |  | -11.75(-31.56,12.89) | 0.23(0.07,0.39) |
| Monaco | 21.66(12.68,32.83) | 920.11(538.79,1394.71) |  | 45.92(26.65,71.92) | 1366.28(792.97,2139.73) |  | 112.02(68.43,164.84) | 0.53(0.26,0.80) |
| Mongolia | 2396.14(1476.62,3548.72) | 427.52(263.46,633.16) |  | 2921.07(1717.00,4484.71) | 419.75(246.73,644.44) |  | 21.91(-3.32,53.82) | -0.40(-0.69,-0.10) |
| Montenegro | 310.35(179.01,473.63) | 284.37(164.02,433.97) |  | 367.66(201.27,592.65) | 488.75(267.56,787.86) |  | 18.46(-7.53,53.45) | 0.36(-0.08,0.80) |
| Morocco | 55858.13(32666.61,83647.17) | 897.27(524.73,1343.65) |  | 83208.01(46432.44,130677.43) | 1272.09(709.86,1997.80) |  | 48.96(12.87,86.33) | 0.44(0.09,0.79) |
| Mozambique | 17821.02(10666.10,27018.13) | 469.57(281.05,711.91) |  | 58559.52(31542.40,91078.18) | 644.45(347.12,1002.31) |  | 228.60(149.42,313.83) | 0.21(-0.09,0.50) |
| Myanmar | 26241.59(15368.87,39397.59) | 269.58(157.89,404.74) |  | 48553.15(26709.91,78231.40) | 467.38(257.11,753.07) |  | 85.02(44.79,135.71) | 0.44(-0.01,0.89) |
| Namibia | 1359.79(790.25,2077.14) | 363.22(211.09,554.83) |  | 3672.55(1978.64,5802.03) | 671.43(361.74,1060.75) |  | 170.08(109.34,237.96) | 0.45(0.01,0.90) |
| Nauru | 7.99(4.60,12.33) | 308.87(177.98,476.61) |  | 10.55(5.80,16.98) | 408.78(224.80,658.15) |  | 32.03(2.31,69.92) | 0.30(0.15,0.46) |
| Nepal | 19200.56(11195.83,28966.86) | 374.15(218.17,564.46) |  | 38388.90(21552.34,61004.82) | 627.18(352.11,996.68) |  | 99.94(52.13,151.18) | 0.84(0.49,1.20) |
| Netherlands | 11341.00(7079.81,16707.05) | 633.95(395.75,933.90) |  | 22809.65(13043.82,35626.66) | 1252.73(716.38,1956.66) |  | 101.13(54.29,162.86) | 1.77(1.40,2.15) |
| New Zealand | 2952.88(1865.72,4264.09) | 567.33(358.46,819.26) |  | 6103.94(3705.62,9300.91) | 912.24(553.81,1390.02) |  | 106.71(66.86,152.71) | 2.06(1.61,2.52) |
| Nicaragua | 3551.73(2073.86,5432.12) | 306.80(179.14,469.23) |  | 5883.50(3218.56,9397.99) | 442.41(242.02,706.68) |  | 65.65(27.31,111.01) | 0.34(0.02,0.65) |
| Niger | 9410.05(5518.86,14018.55) | 396.05(232.28,590.02) |  | 31629.58(17254.36,49347.13) | 412.51(225.03,643.58) |  | 236.13(168.56,315.37) | 0.05(-0.11,0.22) |
| Nigeria | 75708.04(47378.21,108022.72) | 325.92(203.96,465.03) |  | 225336.36(140041.21,323603.92) | 349.51(217.22,501.94) |  | 197.64(181.75,214.30) | 0.10(-0.23,0.43) |
| Niue | 1.72(0.99,2.64) | 311.86(178.76,477.76) |  | 1.15(0.62,1.89) | 426.20(229.39,700.26) |  | -33.19(-48.10,-14.00) | 0.30(0.12,0.47) |
| North Macedonia | 950.74(552.20,1438.59) | 266.74(154.93,403.61) |  | 1150.98(630.80,1830.65) | 506.88(277.80,806.21) |  | 21.06(-6.27,54.17) | 0.58(0.07,1.09) |
| Northern Mariana Islands | 23.36(13.76,35.90) | 315.34(185.71,484.62) |  | 40.41(22.36,63.99) | 501.49(277.54,794.12) |  | 72.99(36.35,116.06) | 0.96(0.60,1.33) |
| Norway | 3883.99(2515.74,5425.13) | 743.36(481.49,1038.31) |  | 5980.03(3849.27,8596.51) | 930.48(598.94,1337.61) |  | 53.97(37.82,69.26) | 0.64(0.32,0.96) |
| Oman | 4077.52(2437.21,6022.75) | 795.31(475.37,1174.72) |  | 8640.48(4755.35,13369.15) | 1081.43(595.17,1673.26) |  | 111.91(65.39,166.12) | 0.21(-0.09,0.50) |
| Pakistan | 84349.80(52961.61,119294.63) | 274.04(172.06,387.56) |  | 187670.87(117185.79,288734.02) | 336.83(210.33,518.22) |  | 122.49(95.29,155.70) | 0.26(-0.00,0.53) |
| Palau | 10.56(6.02,16.39) | 342.88(195.65,532.30) |  | 10.23(5.63,16.50) | 442.44(243.27,713.40) |  | -3.04(-24.23,24.91) | 0.32(0.14,0.50) |
| Palestine | 7005.77(4157.02,10318.75) | 1211.46(718.84,1784.34) |  | 21589.94(12550.94,32713.37) | 1721.12(1000.54,2607.86) |  | 208.17(140.02,289.04) | 0.51(0.25,0.77) |
| Panama | 1455.86(849.29,2228.60) | 265.50(154.88,406.42) |  | 3116.30(1773.34,4952.08) | 398.44(226.73,633.16) |  | 114.05(67.88,164.28) | 0.58(0.18,0.97) |
| Papua New Guinea | 3373.45(1999.21,5093.09) | 320.56(189.97,483.96) |  | 8180.73(4414.57,13087.12) | 341.47(184.27,546.27) |  | 142.50(87.57,203.78) | -0.06(-0.14,0.03) |
| Paraguay | 3140.95(1754.41,4853.41) | 298.41(166.68,461.10) |  | 7223.51(3868.84,11804.66) | 532.06(284.97,869.49) |  | 129.98(79.97,196.28) | 0.76(0.45,1.07) |
| Peru | 10741.34(6189.04,16484.40) | 199.84(115.15,306.69) |  | 22404.49(12045.93,36971.76) | 359.22(193.14,592.79) |  | 108.58(66.21,164.03) | 0.56(-0.03,1.16) |
| Philippines | 54311.74(34576.35,77769.67) | 340.23(216.60,487.18) |  | 114883.34(71428.83,163877.21) | 504.20(313.49,719.23) |  | 111.53(102.82,121.22) | 0.36(-0.07,0.80) |
| Poland | 11301.19(7049.15,16226.06) | 169.74(105.87,243.71) |  | 11336.15(7067.81,16666.77) | 283.15(176.53,416.29) |  | 0.31(-6.33,7.27) | 0.08(-0.39,0.56) |
| Portugal | 19006.04(11470.48,27964.27) | 1236.18(746.06,1818.84) |  | 16619.90(9561.13,25905.86) | 1773.87(1020.48,2764.98) |  | -12.55(-33.33,11.64) | 0.01(-0.47,0.50) |
| Puerto Rico | 2040.80(1180.97,3125.27) | 301.64(174.55,461.93) |  | 1421.00(796.38,2318.22) | 418.84(234.73,683.30) |  | -30.37(-45.57,-10.82) | -0.09(-0.48,0.30) |
| Qatar | 615.88(364.92,911.90) | 829.60(491.56,1228.35) |  | 3054.67(1659.00,4798.26) | 986.65(535.85,1549.82) |  | 395.98(280.06,519.77) | 0.10(-0.11,0.32) |
| Republic of Korea | 27818.06(17007.70,40642.71) | 345.50(211.24,504.79) |  | 23954.16(13586.53,38001.06) | 529.42(300.28,839.88) |  | -13.89(-33.96,11.12) | 1.65(0.98,2.32) |
| Republic of Moldova | 2788.95(1668.70,4165.05) | 346.43(207.27,517.36) |  | 1984.99(1133.50,3123.41) | 539.27(307.94,848.55) |  | -28.83(-43.60,-8.73) | 0.07(-0.37,0.51) |
| Romania | 10796.68(6249.17,15922.10) | 284.23(164.52,419.17) |  | 10103.09(5649.24,16227.62) | 487.56(272.63,783.13) |  | -6.42(-26.17,19.41) | 0.41(-0.10,0.92) |
| Russian Federation | 59936.53(37577.06,85989.16) | 259.75(162.85,372.66) |  | 77625.41(48193.50,111593.71) | 420.35(260.97,604.30) |  | 29.51(24.15,35.16) | -0.12(-0.60,0.37) |
| Rwanda | 10605.42(6359.47,16208.00) | 518.59(310.97,792.55) |  | 22969.68(13184.51,35965.10) | 712.91(409.21,1116.25) |  | 116.58(70.14,173.40) | -0.05(-0.35,0.25) |
| Saint Kitts and Nevis | 32.32(18.82,49.20) | 342.54(199.47,521.44) |  | 33.72(17.93,54.88) | 496.28(263.92,807.65) |  | 4.34(-19.70,32.25) | 0.58(0.36,0.81) |
| Saint Lucia | 112.08(64.50,169.99) | 330.75(190.34,501.66) |  | 119.74(63.63,188.84) | 574.09(305.08,905.37) |  | 6.84(-16.96,36.98) | 0.71(0.38,1.04) |
| Saint Vincent and the Grenadines | 93.37(54.12,141.09) | 329.80(191.17,498.40) |  | 91.55(49.40,151.71) | 515.63(278.26,854.49) |  | -1.95(-24.33,22.66) | 0.48(0.19,0.78) |
| Samoa | 146.75(84.47,225.28) | 320.65(184.56,492.25) |  | 188.33(103.95,303.73) | 371.46(205.03,599.08) |  | 28.33(-0.09,62.20) | -0.00(-0.18,0.17) |
| San Marino | 29.39(17.25,44.84) | 1009.73(592.68,1540.41) |  | 47.19(27.09,76.10) | 1479.94(849.72,2386.76) |  | 60.54(26.84,102.27) | 0.42(-0.02,0.87) |
| Sao Tome and Principe | 152.75(90.78,225.34) | 422.13(250.88,622.73) |  | 267.77(144.83,421.27) | 506.31(273.85,796.53) |  | 75.30(35.38,124.25) | 0.18(-0.06,0.42) |
| Saudi Arabia | 32723.68(19563.66,48614.34) | 791.25(473.05,1175.48) |  | 52688.59(30367.04,81459.38) | 1026.64(591.70,1587.24) |  | 61.01(23.61,109.12) | 0.68(0.45,0.91) |
| Senegal | 8422.40(4872.72,12516.32) | 385.25(222.88,572.51) |  | 22294.97(12802.94,35899.71) | 545.23(313.10,877.93) |  | 164.71(104.46,230.52) | 0.35(0.05,0.66) |
| Serbia | 4278.65(2446.78,6530.58) | 287.52(164.42,438.85) |  | 4152.39(2376.85,6878.33) | 432.92(247.80,717.11) |  | -2.95(-26.67,27.85) | 0.12(-0.32,0.56) |
| Seychelles | 60.55(35.08,93.63) | 386.42(223.88,597.53) |  | 94.47(53.03,152.83) | 608.23(341.40,983.94) |  | 56.02(21.49,98.50) | 0.28(-0.08,0.63) |
| Sierra Leone | 4251.83(2511.49,6327.45) | 409.29(241.76,609.09) |  | 9989.79(5492.11,16277.13) | 447.32(245.92,728.85) |  | 134.95(85.40,196.72) | 0.39(0.25,0.54) |
| Singapore | 2147.94(1327.93,3043.14) | 482.33(298.19,683.35) |  | 2315.21(1276.30,3689.60) | 440.02(242.57,701.23) |  | 7.79(-17.97,38.34) | -0.75(-0.97,-0.53) |
| Slovakia | 2528.20(1458.13,3810.90) | 275.85(159.10,415.81) |  | 2650.10(1469.09,4270.03) | 464.58(257.54,748.57) |  | 4.82(-18.03,33.25) | 0.06(-0.41,0.52) |
| Slovenia | 1031.78(607.54,1548.42) | 355.13(209.11,532.95) |  | 954.74(531.07,1508.76) | 445.38(247.74,703.82) |  | -7.47(-29.34,20.52) | -0.59(-1.02,-0.15) |
| Solomon Islands | 298.87(172.30,447.64) | 311.16(179.39,466.05) |  | 620.48(340.23,1000.59) | 376.72(206.57,607.50) |  | 107.61(60.03,160.68) | 0.04(-0.15,0.24) |
| Somalia | 10969.65(6457.92,16528.61) | 466.67(274.73,703.16) |  | 41872.10(23239.95,65789.35) | 675.32(374.82,1061.06) |  | 281.71(197.49,388.52) | 0.22(-0.11,0.55) |
| South Africa | 30359.90(19485.25,43351.27) | 347.56(223.06,496.28) |  | 58681.35(37182.52,83049.36) | 572.84(362.97,810.72) |  | 93.29(74.79,113.89) | 0.56(0.15,0.97) |
| South Sudan | 7614.65(4553.48,11373.15) | 474.03(283.47,708.01) |  | 15510.91(8875.02,24622.24) | 567.62(324.78,901.05) |  | 103.70(61.52,160.04) | 0.23(-0.01,0.47) |
| Spain | 88827.95(73599.33,104726.54) | 1543.95(1279.26,1820.29) |  | 92184.30(54818.45,144622.21) | 1986.94(1181.56,3117.18) |  | 3.78(-34.48,56.46) | 1.33(0.70,1.97) |
| Sri Lanka | 15886.25(9350.27,24248.83) | 421.92(248.33,644.02) |  | 22080.06(12314.32,34848.27) | 624.01(348.02,984.86) |  | 38.99(7.71,80.87) | -0.06(-0.46,0.34) |
| Sudan | 44986.96(26642.31,67648.59) | 831.31(492.32,1250.08) |  | 119826.13(65365.51,185481.52) | 1094.28(596.93,1693.86) |  | 166.36(106.98,240.66) | 0.33(0.05,0.61) |
| Suriname | 394.84(234.45,595.11) | 456.66(271.16,688.28) |  | 813.89(475.01,1247.34) | 824.35(481.11,1263.36) |  | 106.13(59.14,163.78) | 0.57(0.21,0.94) |
| Sweden | 7202.06(4628.25,10108.17) | 733.87(471.60,1029.99) |  | 12069.09(7324.05,17776.63) | 975.25(591.82,1436.45) |  | 67.58(37.57,106.88) | 0.22(-0.17,0.60) |
| Switzerland | 6695.43(3963.39,10064.53) | 884.09(523.34,1328.96) |  | 9361.05(5193.80,14451.28) | 1051.21(583.24,1622.83) |  | 39.81(9.23,74.06) | -0.08(-0.37,0.21) |
| Syrian Arab Republic | 31166.15(18709.41,46210.89) | 827.75(496.91,1227.33) |  | 34811.42(20256.89,55530.70) | 1309.56(762.04,2089.00) |  | 11.70(-12.52,42.60) | 0.74(0.37,1.11) |
| Taiwan (Province of China) | 9364.38(5503.86,14122.37) | 240.01(141.06,361.96) |  | 5212.41(2969.66,8430.46) | 253.71(144.54,410.34) |  | -44.34(-56.95,-31.07) | -0.29(-0.42,-0.16) |
| Tajikistan | 4345.05(2625.98,6412.72) | 315.66(190.77,465.87) |  | 10554.19(5893.41,16589.14) | 469.94(262.41,738.65) |  | 142.90(84.09,204.63) | 0.38(0.07,0.69) |
| Thailand | 48927.24(29081.73,73898.91) | 419.68(249.45,633.87) |  | 35496.16(19965.89,57842.37) | 511.53(287.72,833.55) |  | -27.45(-44.33,-7.09) | -0.06(-0.28,0.15) |
| Timor-Leste | 709.17(413.89,1077.58) | 368.25(214.92,559.55) |  | 1832.46(1025.04,2897.25) | 545.71(305.26,862.81) |  | 158.40(104.32,226.28) | 0.44(0.18,0.71) |
| Togo | 4444.75(2627.46,6648.98) | 412.99(244.13,617.80) |  | 10285.36(5652.32,16449.62) | 481.40(264.56,769.92) |  | 131.40(81.91,190.16) | 0.22(0.06,0.38) |
| Tokelau | 1.19(0.67,1.82) | 294.26(166.22,448.65) |  | 1.22(0.63,2.02) | 417.88(215.52,691.95) |  | 1.92(-20.21,28.13) | 0.40(0.11,0.70) |
| Tonga | 82.68(48.48,125.27) | 311.62(182.71,472.17) |  | 91.60(50.37,146.50) | 372.16(204.64,595.19) |  | 10.80(-13.62,39.43) | 0.08(-0.07,0.23) |
| Trinidad and Tobago | 1038.74(610.12,1551.75) | 380.49(223.49,568.40) |  | 1325.71(733.46,2141.27) | 690.81(382.19,1115.79) |  | 27.63(-2.82,67.82) | 0.16(-0.27,0.59) |
| Tunisia | 21687.52(12995.77,32714.03) | 1063.99(637.57,1604.95) |  | 38753.98(22951.27,58773.02) | 2068.32(1224.92,3136.75) |  | 78.69(37.50,129.40) | 1.11(0.74,1.47) |
| Turkey | 119985.83(76031.46,174487.70) | 886.57(561.80,1289.29) |  | 196679.45(119659.13,295547.60) | 1516.59(922.69,2278.96) |  | 63.92(13.83,135.72) | 1.55(1.17,1.93) |
| Turkmenistan | 3203.29(1930.02,4813.34) | 349.98(210.87,525.89) |  | 4738.45(2597.34,7615.38) | 481.41(263.88,773.69) |  | 47.92(17.41,88.53) | 0.30(0.02,0.58) |
| Tuvalu | 6.03(3.51,9.14) | 306.63(178.51,464.60) |  | 9.93(5.43,16.24) | 406.27(222.21,664.03) |  | 64.70(27.89,103.86) | 0.48(0.35,0.60) |
| Uganda | 33419.08(20170.99,49506.29) | 692.17(417.78,1025.37) |  | 116362.65(64647.85,179333.72) | 929.56(516.44,1432.61) |  | 248.19(172.22,344.36) | -0.99(-1.65,-0.33) |
| Ukraine | 25995.21(16496.57,37262.33) | 341.77(216.89,489.91) |  | 25782.51(14819.96,41031.30) | 542.45(311.80,863.27) |  | -0.82(-19.69,26.67) | -0.17(-0.66,0.32) |
| United Arab Emirates | 2498.19(1462.08,3703.62) | 691.17(404.51,1024.68) |  | 9211.67(5360.63,14395.92) | 1016.60(591.60,1588.74) |  | 268.73(192.87,365.82) | 0.38(0.11,0.65) |
| United Kingdom | 63411.42(40714.42,87324.43) | 895.77(575.14,1233.57) |  | 95490.75(61644.82,134055.58) | 1174.78(758.39,1649.22) |  | 50.59(44.15,56.55) | -0.25(-0.72,0.22) |
| United Republic of Tanzania | 34996.94(20994.37,51971.58) | 481.55(288.88,715.12) |  | 92882.27(51794.15,146131.23) | 597.05(332.94,939.34) |  | 165.40(105.71,240.08) | 0.17(-0.06,0.40) |
| United States of America | 71.66(41.14,110.60) | 342.20(196.45,528.17) |  | 48.13(26.90,74.66) | 508.17(284.06,788.29) |  | -32.83(-48.90,-15.06) | 0.45(0.15,0.74) |
| United States Virgin Islands | 295912.43(195585.13,404540.66) | 818.35(540.89,1118.76) |  | 783378.24(568928.72,1035109.51) | 1918.15(1393.06,2534.54) |  | 164.73(134.76,222.37) | 2.52(2.01,3.04) |
| Uruguay | 3075.92(1861.33,4700.20) | 563.62(341.07,861.25) |  | 4141.94(2341.99,6675.02) | 889.75(503.09,1433.89) |  | 34.66(6.08,71.08) | 0.58(0.28,0.89) |
| Uzbekistan | 17696.57(10577.32,26393.02) | 341.46(204.09,509.26) |  | 27681.13(15792.77,43690.51) | 442.49(252.45,698.40) |  | 56.42(22.37,94.41) | 0.27(0.02,0.53) |
| Vanuatu | 126.36(75.05,189.74) | 307.84(182.84,462.24) |  | 282.81(162.12,447.23) | 380.17(217.93,601.17) |  | 123.81(76.72,180.62) | 0.08(-0.08,0.25) |
| Venezuela (Bolivarian Republic of) | 12631.19(7198.00,19195.48) | 276.81(157.74,420.66) |  | 15464.26(8438.49,25216.30) | 348.37(190.10,568.06) |  | 22.43(-6.34,54.22) | 0.34(0.15,0.53) |
| Viet Nam | 50687.71(29484.99,75904.74) | 296.37(172.40,443.81) |  | 69115.50(37268.65,112538.18) | 415.84(224.23,677.09) |  | 36.36(6.00,74.72) | 0.08(-0.23,0.38) |
| Yemen | 36470.91(21216.10,53943.35) | 844.67(491.37,1249.33) |  | 85642.64(47381.49,134648.02) | 942.04(521.18,1481.07) |  | 134.82(84.49,202.09) | 0.10(-0.06,0.25) |
| Zambia | 9385.02(5617.05,13923.54) | 417.97(250.16,620.10) |  | 30682.88(17128.68,49134.80) | 573.89(320.37,919.01) |  | 226.93(157.76,300.39) | 0.15(-0.11,0.42) |
| Zimbabwe | 8861.52(5111.72,13217.53) | 290.28(167.44,432.97) |  | 17010.69(9107.55,26671.23) | 416.51(223.00,653.05) |  | 91.96(51.83,138.78) | 0.22(-0.07,0.50) |
