# Supplementary material for: Major depressive disorders in children aged 5–14 years: a Global Burden of Disease analysis from the perspective of exercise psychology
Source: Front Public Health. 2025 Oct 29;13:1671222. doi: 10.3389/fpubh.2025.1671222 (PMC12605264; doi:10.3389/fpubh.2025.1671222)
Supplement: Supplementary file 2 [file Table_2.docx]

Table S2: Incidence of major depressive disorders in children between 1990 and 2021 at the national level.

| location | 1990 | |  | 2021 | |  | 1990-2021 | |
| --- | --- | --- | --- | --- | --- | --- | --- | --- |
|  | Incident cases | Incidence rate |  | Incident cases | Incidence rate |  | Cases change | EAPC |
| Afghanistan | 42705.03(25272.56,63629.61) | 1646.37(974.31,2453.05) |  | 185097.38(105624.37,285558.78) | 2122.87(1211.40,3275.06) |  | 333.43(244.88,443.90) | 0.54(0.22,0.86) |
| Albania | 3254.15(1918.31,4949.15) | 456.09(268.87,693.66) |  | 2408.57(1306.23,3927.66) | 799.98(433.85,1304.52) |  | -25.98(-42.69,-6.66) | 0.82(0.47,1.17) |
| Algeria | 104995.53(62755.18,154475.90) | 1502.37(897.96,2210.38) |  | 155591.06(86574.33,247093.02) | 1809.82(1007.03,2874.17) |  | 48.19(16.02,86.57) | 0.06(-0.19,0.32) |
| American Samoa | 62.80(37.14,93.70) | 550.82(325.73,821.76) |  | 75.43(40.68,122.78) | 720.26(388.44,1172.42) |  | 20.10(-5.60,49.01) | 0.35(0.17,0.53) |
| Andorra | 110.28(66.91,162.47) | 1623.21(984.84,2391.32) |  | 186.49(104.74,286.02) | 2440.49(1370.61,3742.99) |  | 69.10(33.24,114.59) | 0.43(-0.07,0.94) |
| Angola | 29396.31(17339.75,44591.62) | 1064.07(627.65,1614.10) |  | 123801.84(67705.40,197652.26) | 1287.96(704.36,2056.25) |  | 321.15(230.46,431.29) | 0.04(-0.23,0.31) |
| Antigua and Barbuda | 70.34(41.17,109.22) | 579.87(339.42,900.43) |  | 104.01(53.45,172.27) | 894.36(459.62,1481.33) |  | 47.88(16.33,83.70) | 0.58(0.27,0.89) |
| Argentina | 65814.32(38945.89,98906.83) | 982.53(581.42,1476.57) |  | 111389.72(63392.71,168035.86) | 1549.86(882.03,2338.02) |  | 69.25(28.84,121.07) | 0.70(0.37,1.03) |
| Armenia | 3802.69(2218.31,5666.91) | 575.92(335.97,858.26) |  | 4270.85(2288.05,6923.01) | 1051.52(563.34,1704.51) |  | 12.31(-14.29,43.24) | 0.65(0.18,1.13) |
| Australia | 48692.29(30444.26,69605.68) | 1929.78(1206.57,2758.63) |  | 72335.53(42221.15,109748.63) | 2228.47(1300.72,3381.07) |  | 48.56(15.07,91.68) | 0.33(0.13,0.54) |
| Austria | 11073.99(6616.98,16586.24) | 1228.64(734.14,1840.21) |  | 14448.92(7922.28,22990.79) | 1670.58(915.97,2658.19) |  | 30.48(0.30,64.57) | 0.06(-0.28,0.39) |
| Azerbaijan | 8707.58(5118.05,13089.55) | 571.67(336.01,859.36) |  | 15196.74(8630.34,24183.60) | 926.89(526.39,1475.02) |  | 74.52(37.36,119.43) | 0.49(0.09,0.90) |
| Bahamas | 325.24(184.96,509.21) | 589.66(335.34,923.19) |  | 612.91(332.37,1042.57) | 1019.35(552.76,1733.92) |  | 88.45(46.30,134.98) | 0.79(0.35,1.23) |
| Bahrain | 1660.90(995.03,2476.82) | 1631.05(977.14,2432.31) |  | 4687.83(2598.87,7487.41) | 2309.03(1280.09,3687.98) |  | 182.25(121.54,259.77) | 0.17(-0.12,0.46) |
| Bangladesh | 219566.49(131688.90,322906.71) | 732.36(439.25,1077.05) |  | 305078.75(166662.45,479536.61) | 971.53(530.74,1527.09) |  | 38.95(8.69,77.57) | 0.21(-0.11,0.54) |
| Barbados | 265.31(152.99,411.10) | 620.19(357.63,960.97) |  | 342.00(175.46,548.10) | 1021.72(524.19,1637.44) |  | 28.91(-3.02,61.60) | 0.48(0.11,0.86) |
| Belarus | 10503.66(6119.45,16301.44) | 657.25(382.91,1020.03) |  | 13564.27(7517.78,21932.50) | 1221.11(676.78,1974.45) |  | 29.14(-0.49,65.58) | 0.14(-0.40,0.68) |
| Belgium | 16726.87(10893.53,23516.38) | 1382.82(900.57,1944.11) |  | 25959.10(14946.44,40099.81) | 1966.34(1132.16,3037.47) |  | 55.19(15.37,102.69) | 0.46(0.07,0.85) |
| Belize | 304.06(177.93,470.75) | 581.18(340.09,899.78) |  | 772.08(421.27,1262.13) | 908.50(495.71,1485.13) |  | 153.92(100.23,221.31) | 0.75(0.44,1.05) |
| Benin | 10419.31(6238.10,15680.95) | 726.05(434.69,1092.70) |  | 33796.01(18603.44,54184.61) | 904.11(497.68,1449.55) |  | 224.36(158.43,314.37) | 0.37(0.23,0.52) |
| Bermuda | 52.79(29.59,81.15) | 692.31(388.09,1064.19) |  | 58.94(31.13,95.74) | 1001.37(528.97,1626.68) |  | 11.64(-14.28,42.46) | 0.10(-0.26,0.45) |
| Bhutan | 1042.39(608.31,1584.96) | 625.21(364.85,950.63) |  | 849.30(477.49,1372.42) | 673.32(378.55,1088.05) |  | -18.52(-38.61,1.11) | 0.25(0.11,0.39) |
| Bolivia (Plurinational State of) | 7754.38(4431.33,12180.60) | 462.70(264.42,726.81) |  | 19809.38(10718.79,32358.17) | 864.16(467.59,1411.58) |  | 155.46(96.28,235.44) | 0.72(0.10,1.34) |
| Bosnia and Herzegovina | 4947.42(2865.82,7532.74) | 664.98(385.19,1012.47) |  | 3106.61(1707.66,4841.36) | 915.30(503.13,1426.41) |  | -37.21(-51.51,-18.67) | -0.46(-0.89,-0.02) |
| Botswana | 2883.64(1691.52,4347.14) | 764.03(448.18,1151.80) |  | 5743.66(3144.51,9137.84) | 1241.12(679.48,1974.56) |  | 99.18(55.77,157.61) | 0.33(0.01,0.65) |
| Brazil | 211222.33(132513.47,311782.29) | 595.29(373.46,878.70) |  | 236212.64(144728.43,353697.08) | 746.85(457.60,1118.31) |  | 11.83(3.12,21.46) | -1.06(-2.14,0.02) |
| Brunei Darussalam | 338.92(198.56,504.71) | 604.21(353.98,899.78) |  | 514.08(278.03,843.07) | 807.00(436.45,1323.45) |  | 51.68(18.13,92.07) | 0.53(0.32,0.73) |
| Bulgaria | 6980.87(3991.63,10718.06) | 582.03(332.80,893.61) |  | 6915.45(3604.76,10934.08) | 1026.97(535.32,1623.75) |  | -0.94(-24.18,27.02) | -0.19(-0.83,0.45) |
| Burkina Faso | 22275.16(13315.43,33863.52) | 783.64(468.44,1191.32) |  | 54386.04(30278.00,86891.35) | 868.00(483.23,1386.78) |  | 144.16(93.34,210.03) | 0.24(0.10,0.38) |
| Burundi | 13765.10(8203.46,20724.39) | 890.35(530.62,1340.49) |  | 42533.70(22916.91,69249.26) | 1151.50(620.42,1874.76) |  | 209.00(134.50,292.91) | -0.03(-0.31,0.25) |
| Cabo Verde | 785.43(456.66,1193.73) | 802.94(466.83,1220.34) |  | 1276.27(697.46,2061.26) | 1287.31(703.49,2079.09) |  | 62.49(27.27,106.35) | 0.69(0.37,1.01) |
| Cambodia | 20638.67(11900.69,31066.79) | 727.76(419.64,1095.48) |  | 36474.31(19560.37,60077.89) | 1082.96(580.77,1783.78) |  | 76.73(38.75,123.99) | 0.25(-0.02,0.53) |
| Cameroon | 22419.72(13102.93,34019.87) | 779.64(455.65,1183.04) |  | 80422.80(43502.41,128757.12) | 934.80(505.65,1496.62) |  | 258.71(183.87,347.68) | 0.21(0.06,0.37) |
| Canada | 46552.27(28804.99,67571.79) | 1216.53(752.75,1765.82) |  | 83205.66(45269.23,134089.03) | 1947.82(1059.74,3138.99) |  | 78.74(33.71,130.05) | 0.41(0.07,0.76) |
| Central African Republic | 7342.81(4334.22,11078.92) | 1021.28(602.83,1540.92) |  | 17737.73(9740.88,28738.06) | 1227.54(674.12,1988.82) |  | 141.57(85.57,202.96) | 0.35(0.13,0.57) |
| Chad | 15879.54(9336.98,24454.81) | 943.28(554.64,1452.66) |  | 54083.36(29131.11,87538.76) | 1005.38(541.53,1627.30) |  | 240.59(170.20,329.59) | 0.15(0.01,0.30) |
| Chile | 43233.89(26823.31,63567.35) | 1705.57(1058.18,2507.72) |  | 60700.21(34369.90,94140.87) | 2367.51(1340.54,3671.81) |  | 40.40(4.87,85.61) | 0.19(-0.21,0.58) |
| China | 901706.34(575143.51,1297997.43) | 436.51(278.42,628.36) |  | 617688.94(384696.99,878320.11) | 339.46(211.41,482.69) |  | -31.50(-36.69,-25.09) | -0.38(-0.84,0.08) |
| Colombia | 30382.44(17754.24,46758.09) | 407.49(238.12,627.11) |  | 40067.65(21818.79,65452.16) | 558.80(304.30,912.83) |  | 31.88(2.26,71.81) | 0.22(-0.10,0.54) |
| Comoros | 1006.05(598.66,1524.95) | 772.25(459.54,1170.56) |  | 1676.53(929.75,2749.97) | 1055.44(585.31,1731.21) |  | 66.64(32.95,111.83) | 0.36(0.10,0.62) |
| Congo | 7159.21(4198.89,10915.25) | 1087.24(637.67,1657.65) |  | 17640.70(9060.30,28099.68) | 1360.71(698.86,2167.47) |  | 146.41(88.46,213.43) | 0.04(-0.24,0.32) |
| Cook Islands | 27.13(15.45,42.07) | 622.95(354.82,966.19) |  | 21.85(11.69,35.62) | 820.68(438.80,1337.68) |  | -19.43(-35.80,2.37) | 0.44(0.25,0.64) |
| Costa Rica | 3623.47(2130.25,5614.74) | 504.51(296.60,781.76) |  | 5686.48(3155.66,9208.71) | 802.05(445.09,1298.84) |  | 56.93(24.55,98.80) | 0.60(0.29,0.91) |
| Croatia | 4155.86(2454.90,6359.74) | 606.04(357.99,927.43) |  | 3552.56(1894.86,5650.31) | 856.26(456.71,1361.87) |  | -14.52(-34.00,8.80) | -0.06(-0.45,0.33) |
| Cuba | 12492.65(7090.01,19073.43) | 776.44(440.66,1185.44) |  | 11820.81(6683.77,18900.55) | 959.05(542.27,1533.45) |  | -5.38(-26.88,22.53) | -0.41(-0.76,-0.05) |
| Cyprus | 1934.65(1175.68,2908.81) | 1443.40(877.15,2170.20) |  | 2773.17(1607.02,4294.20) | 1930.41(1118.65,2989.20) |  | 43.34(12.68,79.52) | 0.22(-0.03,0.47) |
| Czechia | 9511.96(5378.16,14820.84) | 611.75(345.89,953.19) |  | 10089.63(5460.99,16313.38) | 874.11(473.11,1413.30) |  | 6.07(-17.33,35.30) | -0.21(-0.71,0.28) |
| C么te d'Ivoire | 22817.42(13542.60,34307.91) | 672.51(399.15,1011.18) |  | 54164.74(29932.40,86525.91) | 750.48(414.73,1198.85) |  | 137.38(85.92,197.66) | 0.07(-0.07,0.22) |
| Democratic People's Republic of Korea | 16864.56(10077.73,25790.99) | 466.78(278.93,713.85) |  | 16570.44(8977.98,25744.26) | 508.20(275.35,789.56) |  | -1.74(-20.65,24.61) | 0.11(-0.00,0.22) |
| Democratic Republic of the Congo | 106241.47(61567.44,160478.27) | 1020.75(591.53,1541.85) |  | 295120.75(158658.16,470845.70) | 1207.62(649.22,1926.68) |  | 177.78(114.05,251.00) | 0.23(0.01,0.45) |
| Denmark | 8762.60(5135.28,13207.43) | 1475.82(864.90,2224.43) |  | 12311.22(7205.68,19061.00) | 1913.26(1119.82,2962.23) |  | 40.50(8.50,79.37) | 0.52(0.21,0.83) |
| Djibouti | 954.62(564.07,1440.36) | 871.92(515.20,1315.58) |  | 2924.38(1584.57,4598.53) | 1093.33(592.42,1719.24) |  | 206.34(145.60,278.83) | 0.12(-0.14,0.38) |
| Dominica | 95.06(54.45,148.12) | 588.49(337.07,917.00) |  | 96.92(52.11,154.84) | 949.75(510.67,1517.37) |  | 1.95(-19.02,28.85) | 0.57(0.23,0.91) |
| Dominican Republic | 11842.85(6850.25,18151.23) | 696.80(403.05,1067.97) |  | 17227.26(9437.76,27935.74) | 904.16(495.34,1466.19) |  | 45.47(13.87,82.67) | 0.41(0.12,0.70) |
| Ecuador | 15407.41(8688.11,23747.73) | 611.47(344.80,942.47) |  | 35184.07(18976.67,55962.79) | 1031.86(556.54,1641.25) |  | 128.36(73.33,197.05) | 0.77(0.29,1.25) |
| Egypt | 184700.99(110609.21,277575.07) | 1353.23(810.39,2033.68) |  | 443565.21(251732.08,690778.89) | 1862.33(1056.91,2900.27) |  | 140.15(87.13,202.09) | 0.32(0.04,0.61) |
| El Salvador | 9352.68(5467.48,14204.12) | 673.82(393.91,1023.35) |  | 9566.15(5024.11,15459.62) | 785.36(412.47,1269.19) |  | 2.28(-21.57,34.37) | -0.08(-0.40,0.24) |
| Equatorial Guinea | 1196.62(707.92,1827.32) | 1043.66(617.43,1593.75) |  | 5130.23(2843.58,8161.93) | 1290.10(715.07,2052.48) |  | 328.73(238.81,439.01) | 0.28(0.04,0.52) |
| Eritrea | 8483.71(5141.00,12768.11) | 876.06(530.88,1318.49) |  | 17011.98(9601.72,27669.08) | 1058.89(597.65,1722.22) |  | 100.53(55.06,152.88) | 0.16(-0.00,0.31) |
| Estonia | 1863.03(1108.29,2777.17) | 815.33(485.03,1215.40) |  | 1755.93(980.01,2738.38) | 1194.86(666.86,1863.39) |  | -5.75(-27.23,21.57) | -0.63(-1.15,-0.10) |
| Eswatini | 1787.44(1035.09,2735.23) | 743.76(430.71,1138.14) |  | 4233.88(2293.37,6844.88) | 1555.32(842.47,2514.48) |  | 136.87(80.24,209.05) | 0.74(0.25,1.23) |
| Ethiopia | 111903.66(71214.34,158316.74) | 759.98(483.64,1075.19) |  | 262921.49(163262.33,394210.31) | 926.26(575.16,1388.78) |  | 134.95(107.45,164.44) | -0.03(-0.35,0.29) |
| Fiji | 1076.26(633.61,1627.51) | 574.93(338.47,869.40) |  | 1486.73(807.99,2349.23) | 819.22(445.22,1294.48) |  | 38.14(9.60,75.78) | 0.18(-0.07,0.44) |
| Finland | 15185.71(9246.29,22123.95) | 2328.14(1417.56,3391.86) |  | 14179.03(8159.54,21972.87) | 2346.39(1350.26,3636.13) |  | -6.63(-27.74,19.93) | -0.98(-1.46,-0.51) |
| France | 141530.62(93294.35,197228.49) | 1808.74(1192.29,2520.55) |  | 184500.36(104372.25,278168.20) | 2285.20(1292.74,3445.36) |  | 30.36(-5.43,70.62) | -0.22(-0.67,0.23) |
| Gabon | 2689.19(1575.09,4047.42) | 1070.06(626.75,1610.51) |  | 6139.89(3367.28,9840.16) | 1442.64(791.18,2312.06) |  | 128.32(82.70,187.62) | 0.34(0.10,0.58) |
| Gambia | 2976.00(1752.09,4515.91) | 1079.90(635.78,1638.69) |  | 8769.49(4805.73,13634.90) | 1379.16(755.78,2144.33) |  | 194.67(127.36,267.94) | 0.31(0.09,0.53) |
| Georgia | 5830.77(3451.12,8855.68) | 647.76(383.40,983.80) |  | 5120.72(2805.18,8300.50) | 1039.44(569.42,1684.90) |  | -12.18(-31.69,13.71) | 0.38(0.00,0.75) |
| Germany | 109956.07(71637.56,153079.04) | 1298.70(846.12,1808.03) |  | 163721.84(91510.17,261339.62) | 2066.89(1155.26,3299.26) |  | 48.90(8.22,100.28) | 0.66(0.06,1.27) |
| Ghana | 32718.93(19093.15,48880.02) | 801.43(467.67,1197.29) |  | 82382.39(46549.26,137485.17) | 999.43(564.72,1667.91) |  | 151.79(96.68,212.81) | 0.30(0.11,0.49) |
| Greece | 33079.72(19803.04,49111.64) | 2252.46(1348.43,3344.11) |  | 33741.97(19495.76,53449.91) | 3470.90(2005.45,5498.18) |  | 2.00(-19.53,27.30) | 0.18(-0.21,0.57) |
| Greenland | 211.09(129.64,314.01) | 2421.73(1487.23,3602.47) |  | 288.44(163.86,460.50) | 3740.94(2125.21,5972.39) |  | 36.64(4.33,76.10) | 0.42(0.09,0.76) |
| Grenada | 125.41(73.06,195.40) | 586.04(341.41,913.12) |  | 140.80(75.74,228.68) | 941.08(506.20,1528.39) |  | 12.28(-12.05,41.32) | 0.57(0.28,0.87) |
| Guam | 159.96(93.23,244.59) | 626.51(365.17,957.98) |  | 227.81(124.92,357.86) | 957.26(524.91,1503.69) |  | 42.42(12.98,77.36) | 0.68(0.40,0.95) |
| Guatemala | 14352.77(8385.86,22047.08) | 567.93(331.82,872.39) |  | 30116.38(16520.76,48684.03) | 892.40(489.54,1442.59) |  | 109.83(64.16,168.13) | 0.70(0.39,1.02) |
| Guinea | 11758.39(6882.53,17519.76) | 738.92(432.51,1100.97) |  | 35929.59(19502.15,55590.50) | 946.69(513.85,1464.73) |  | 205.57(136.88,299.58) | 0.36(0.15,0.56) |
| Guinea-Bissau | 2250.02(1313.16,3390.54) | 764.48(446.16,1151.98) |  | 5304.33(3039.79,8445.28) | 938.00(537.55,1493.44) |  | 135.75(84.24,196.24) | 0.21(0.00,0.41) |
| Guyana | 1460.16(850.05,2243.21) | 806.13(469.30,1238.44) |  | 1882.05(1032.21,3124.36) | 1355.21(743.27,2249.77) |  | 28.89(0.90,65.99) | 1.12(0.78,1.46) |
| Haiti | 10224.35(5941.13,15274.39) | 618.76(359.55,924.39) |  | 23875.45(12862.41,37756.31) | 857.93(462.19,1356.71) |  | 133.52(87.32,193.52) | 0.23(-0.04,0.49) |
| Honduras | 6357.00(3623.05,9745.05) | 458.52(261.32,702.89) |  | 17481.78(9238.80,27783.72) | 801.31(423.48,1273.52) |  | 175.00(114.70,250.63) | 0.76(0.35,1.18) |
| Hungary | 8991.01(5122.99,14127.67) | 594.69(338.85,934.44) |  | 7134.15(3958.03,11269.18) | 763.74(423.73,1206.42) |  | -20.65(-39.34,0.54) | -0.19(-0.55,0.18) |
| Iceland | 560.36(334.40,826.21) | 1324.45(790.38,1952.78) |  | 723.68(408.32,1155.90) | 1588.25(896.13,2536.82) |  | 29.14(0.78,67.37) | 0.09(-0.16,0.35) |
| India | 840879.60(531594.49,1233382.09) | 399.89(252.81,586.55) |  | 2075418.03(1313839.38,3052719.71) | 813.69(515.10,1196.85) |  | 146.82(132.26,162.40) | 1.91(1.54,2.28) |
| Indonesia | 233078.52(147898.50,333502.00) | 513.20(325.65,734.32) |  | 368478.15(229962.17,540953.04) | 811.95(506.73,1192.00) |  | 58.09(48.27,69.33) | 0.41(0.03,0.79) |
| Iran (Islamic Republic of) | 201510.55(127375.88,288917.69) | 1213.01(766.75,1739.17) |  | 259056.06(162031.22,373121.76) | 1847.20(1155.36,2660.54) |  | 28.56(20.75,36.87) | 0.50(0.17,0.83) |
| Iraq | 64001.80(38540.46,93542.17) | 1256.48(756.63,1836.42) |  | 142184.04(80149.57,216788.62) | 1550.79(874.19,2364.50) |  | 122.16(74.31,183.04) | 0.46(0.03,0.89) |
| Ireland | 11602.75(7020.53,17065.78) | 1673.00(1012.29,2460.71) |  | 17552.90(9947.31,27895.53) | 2511.87(1423.49,3991.93) |  | 51.28(19.13,92.57) | 0.07(-0.35,0.49) |
| Israel | 18678.81(11204.82,28002.92) | 1837.20(1102.08,2754.30) |  | 38591.44(22523.53,59972.86) | 2257.51(1317.58,3508.28) |  | 106.61(62.83,160.36) | 0.02(-0.29,0.33) |
| Italy | 94749.84(61563.73,132684.71) | 1461.28(949.47,2046.33) |  | 115646.18(75321.08,165673.15) | 2129.86(1387.19,3051.21) |  | 22.05(13.67,31.33) | 0.28(-0.21,0.78) |
| Jamaica | 3281.33(1895.14,5072.88) | 589.96(340.73,912.06) |  | 3938.41(2039.15,6397.12) | 954.19(494.04,1549.88) |  | 20.02(-7.00,55.71) | 0.63(0.32,0.95) |
| Japan | 92553.72(58998.50,133048.64) | 563.30(359.07,809.76) |  | 85369.58(53499.56,121084.72) | 786.17(492.68,1115.07) |  | -7.76(-13.32,-1.87) | 0.37(0.04,0.70) |
| Jordan | 15729.54(9265.07,23732.62) | 1522.44(896.76,2297.05) |  | 50690.92(28816.27,81678.73) | 1999.07(1136.41,3221.12) |  | 222.27(158.73,301.45) | 0.40(0.17,0.62) |
| Kazakhstan | 23126.75(14155.81,33856.15) | 697.99(427.24,1021.82) |  | 31806.41(17374.36,50980.35) | 914.49(499.54,1465.78) |  | 37.53(4.05,75.76) | 0.04(-0.32,0.40) |
| Kenya | 48859.95(31459.99,70134.53) | 710.47(457.46,1019.82) |  | 116128.90(74245.62,168992.26) | 913.32(583.92,1329.07) |  | 137.68(127.02,147.31) | 0.25(-0.03,0.53) |
| Kiribati | 101.41(60.72,151.75) | 571.10(341.97,854.59) |  | 196.71(108.75,315.21) | 709.54(392.26,1136.95) |  | 93.98(48.33,147.48) | 0.07(-0.11,0.25) |
| Kuwait | 5242.37(3182.32,7799.28) | 1498.27(909.51,2229.03) |  | 9724.11(5484.87,15534.89) | 1675.20(944.90,2676.25) |  | 85.49(42.80,135.47) | 0.26(0.05,0.47) |
| Kyrgyzstan | 7270.57(4428.00,10901.36) | 702.58(427.89,1053.44) |  | 14875.05(8445.69,23190.21) | 1004.74(570.47,1566.39) |  | 104.59(57.28,155.85) | 0.22(-0.14,0.59) |
| Lao People's Democratic Republic | 7464.51(4297.25,11338.80) | 659.32(379.56,1001.52) |  | 13488.72(7386.89,21384.68) | 919.58(503.59,1457.88) |  | 80.70(40.38,127.71) | 0.55(0.32,0.77) |
| Latvia | 2510.75(1482.78,3708.10) | 685.39(404.77,1012.25) |  | 2416.93(1312.12,3823.67) | 1188.59(645.27,1880.40) |  | -3.74(-26.96,24.30) | -0.12(-0.67,0.43) |
| Lebanon | 9789.16(5929.53,14236.59) | 1491.65(903.53,2169.34) |  | 25612.60(14645.30,40411.16) | 2936.69(1679.20,4633.47) |  | 161.64(97.91,238.37) | 0.52(0.10,0.94) |
| Lesotho | 5182.68(3053.49,8117.95) | 1187.90(699.88,1860.69) |  | 9028.00(5157.83,14485.80) | 2115.41(1208.56,3394.26) |  | 74.20(32.48,127.69) | 0.60(0.17,1.04) |
| Liberia | 5455.47(3207.57,8292.30) | 819.44(481.80,1245.55) |  | 14214.59(7558.89,22878.22) | 1001.35(532.49,1611.66) |  | 160.56(105.49,234.97) | 0.68(0.42,0.94) |
| Libya | 18861.51(11402.64,27524.17) | 1606.58(971.25,2344.45) |  | 22570.37(12551.93,35249.42) | 2112.25(1174.67,3298.82) |  | 19.66(-5.51,49.71) | 0.41(0.15,0.68) |
| Lithuania | 3868.39(2249.06,5809.36) | 714.23(415.25,1072.59) |  | 3471.67(1916.96,5608.12) | 1256.96(694.06,2030.49) |  | -10.26(-31.23,15.40) | 0.17(-0.32,0.67) |
| Luxembourg | 631.17(382.29,941.05) | 1461.88(885.43,2179.62) |  | 1273.81(741.05,2050.82) | 1868.71(1087.14,3008.60) |  | 101.82(57.11,156.62) | 0.11(-0.24,0.46) |
| Madagascar | 28855.13(17298.74,43914.25) | 874.30(524.15,1330.59) |  | 88455.20(46436.39,140860.87) | 1156.96(607.37,1842.41) |  | 206.55(134.26,284.14) | 0.29(0.02,0.56) |
| Malawi | 20017.86(11938.63,30003.72) | 755.14(450.36,1131.84) |  | 58878.98(32819.45,94786.89) | 1090.39(607.79,1755.38) |  | 194.13(125.19,273.72) | 0.29(-0.04,0.62) |
| Malaysia | 35148.72(20127.36,53985.92) | 838.74(480.29,1288.24) |  | 66702.89(35795.09,107856.43) | 1293.89(694.35,2092.18) |  | 89.77(46.13,141.08) | 3.54(2.45,4.63) |
| Maldives | 499.61(294.88,773.39) | 789.78(466.14,1222.56) |  | 713.46(383.17,1157.99) | 1042.91(560.10,1692.69) |  | 42.80(10.07,81.38) | -0.13(-0.44,0.18) |
| Mali | 14727.58(8749.39,21742.80) | 613.49(364.46,905.71) |  | 50614.54(27984.55,79356.89) | 723.54(400.04,1134.41) |  | 243.67(167.50,330.02) | 0.23(0.01,0.45) |
| Malta | 880.72(520.17,1312.84) | 1485.76(877.51,2214.74) |  | 784.61(441.46,1213.36) | 1867.61(1050.81,2888.17) |  | -10.91(-29.96,13.19) | 0.24(0.00,0.48) |
| Marshall Islands | 81.29(48.61,122.93) | 560.41(335.12,847.49) |  | 85.11(45.02,138.89) | 722.27(382.03,1178.58) |  | 4.70(-19.34,34.05) | 0.11(-0.08,0.31) |
| Mauritania | 3638.99(2138.99,5490.47) | 657.55(386.51,992.11) |  | 9614.70(5375.51,15647.65) | 803.90(449.45,1308.32) |  | 164.21(105.80,243.13) | 0.24(-0.01,0.48) |
| Mauritius | 3160.31(1808.87,4834.59) | 1407.18(805.43,2152.67) |  | 2771.66(1460.66,4357.88) | 1936.75(1020.66,3045.16) |  | -12.30(-32.78,11.61) | 0.19(-0.18,0.56) |
| Mexico | 95302.25(59966.64,140066.73) | 440.84(277.39,647.91) |  | 178296.01(111951.62,263586.89) | 803.50(504.52,1187.87) |  | 87.08(75.23,99.43) | 0.85(0.42,1.27) |
| Micronesia (Federated States of) | 177.23(104.25,268.48) | 585.56(344.43,887.02) |  | 156.01(87.84,248.28) | 738.31(415.72,1174.98) |  | -11.97(-31.68,12.89) | 0.22(0.06,0.38) |
| Monaco | 38.65(22.78,58.18) | 1641.79(967.88,2471.51) |  | 82.12(47.45,130.19) | 2443.29(1411.69,3873.46) |  | 112.48(69.27,165.82) | 0.53(0.26,0.80) |
| Mongolia | 4450.66(2729.01,6701.06) | 794.08(486.91,1195.59) |  | 5425.26(3142.74,8473.78) | 779.59(451.60,1217.66) |  | 21.90(-3.34,53.64) | -0.40(-0.69,-0.11) |
| Montenegro | 570.51(328.38,873.92) | 522.75(300.89,800.76) |  | 674.63(361.15,1097.07) | 896.84(480.11,1458.41) |  | 18.25(-8.56,53.18) | 0.35(-0.09,0.79) |
| Morocco | 101562.58(60845.16,149914.59) | 1631.43(977.37,2408.12) |  | 151243.43(84113.49,238615.42) | 2312.21(1285.93,3647.96) |  | 48.92(13.41,84.73) | 0.44(0.09,0.79) |
| Mozambique | 32986.75(19550.97,50753.44) | 869.18(515.16,1337.32) |  | 108329.06(59486.85,169107.78) | 1192.16(654.65,1861.03) |  | 228.40(153.55,319.57) | 0.21(-0.08,0.50) |
| Myanmar | 48346.95(28612.25,73691.48) | 496.68(293.94,757.04) |  | 89462.22(49672.34,141679.68) | 861.18(478.15,1363.83) |  | 85.04(43.96,133.63) | 0.44(-0.02,0.90) |
| Namibia | 2510.06(1485.30,3741.83) | 670.47(396.74,999.49) |  | 6771.61(3590.57,10631.42) | 1238.01(656.44,1943.68) |  | 169.78(111.25,239.57) | 0.45(0.00,0.89) |
| Nauru | 14.83(8.59,23.13) | 573.19(331.96,894.36) |  | 19.55(10.86,32.03) | 757.71(420.74,1241.23) |  | 31.88(1.00,69.42) | 0.30(0.14,0.46) |
| Nepal | 35526.18(21057.77,53927.39) | 692.27(410.34,1050.85) |  | 71007.94(40186.85,111722.25) | 1160.10(656.56,1825.28) |  | 99.87(53.56,152.15) | 0.84(0.49,1.19) |
| Netherlands | 19764.08(12506.00,28193.28) | 1104.79(699.07,1575.97) |  | 40363.41(22947.36,62074.61) | 2216.81(1260.30,3409.21) |  | 104.23(54.56,168.22) | 1.83(1.46,2.21) |
| New Zealand | 5482.63(3426.58,8051.39) | 1053.38(658.35,1546.91) |  | 11443.12(6876.69,17414.79) | 1710.18(1027.72,2602.64) |  | 108.72(67.43,155.23) | 2.11(1.65,2.57) |
| Nicaragua | 6643.06(3881.28,10047.43) | 573.83(335.27,867.90) |  | 10995.52(5992.46,17635.56) | 826.80(450.60,1326.10) |  | 65.52(27.28,111.23) | 0.33(0.01,0.65) |
| Niger | 17425.86(10316.19,26305.38) | 733.42(434.19,1107.15) |  | 58507.15(31591.61,90874.47) | 763.04(412.01,1185.17) |  | 235.75(165.60,317.83) | 0.05(-0.11,0.22) |
| Nigeria | 141044.07(89747.83,200544.42) | 607.18(386.36,863.32) |  | 419175.23(263094.33,613158.68) | 650.17(408.08,951.06) |  | 197.19(180.65,213.85) | 0.09(-0.24,0.43) |
| Niue | 3.20(1.83,4.94) | 578.64(331.59,893.94) |  | 2.13(1.14,3.50) | 789.17(420.61,1295.46) |  | -33.32(-47.87,-14.02) | 0.29(0.12,0.47) |
| North Macedonia | 1742.39(1032.04,2646.03) | 488.84(289.55,742.36) |  | 2108.93(1149.14,3360.22) | 928.76(506.07,1479.82) |  | 21.04(-5.82,54.12) | 0.58(0.06,1.09) |
| Northern Mariana Islands | 43.15(25.60,67.68) | 582.55(345.65,913.69) |  | 74.64(41.20,119.78) | 926.37(511.33,1486.57) |  | 72.98(35.35,115.01) | 0.95(0.59,1.32) |
| Norway | 7106.62(4570.78,10044.48) | 1360.13(874.80,1922.41) |  | 10926.02(6942.34,15822.91) | 1700.08(1080.22,2462.03) |  | 53.74(37.38,69.08) | 0.61(0.31,0.92) |
| Oman | 7400.26(4455.12,10915.59) | 1443.40(868.96,2129.05) |  | 15693.59(8846.67,24641.32) | 1964.18(1107.23,3084.06) |  | 112.07(63.64,169.90) | 0.22(-0.07,0.51) |
| Pakistan | 157193.75(100074.76,223988.54) | 510.69(325.12,727.69) |  | 349370.51(213522.33,531179.47) | 627.06(383.23,953.37) |  | 122.25(95.43,157.32) | 0.26(-0.01,0.52) |
| Palau | 19.55(11.22,30.78) | 634.94(364.33,999.78) |  | 18.95(10.07,30.79) | 819.03(435.19,1331.21) |  | -3.08(-24.35,23.91) | 0.31(0.13,0.50) |
| Palestine | 12770.33(7678.65,19080.89) | 2208.28(1327.81,3299.51) |  | 39269.16(23033.16,59240.23) | 3130.48(1836.17,4722.55) |  | 207.50(141.01,289.27) | 0.50(0.24,0.76) |
| Panama | 2702.26(1556.60,4169.65) | 492.80(283.87,760.40) |  | 5791.02(3276.08,9254.14) | 740.42(418.87,1183.20) |  | 114.30(67.38,165.02) | 0.58(0.19,0.97) |
| Papua New Guinea | 6261.60(3735.54,9451.93) | 595.00(354.96,898.16) |  | 15163.45(8046.45,24570.96) | 632.94(335.87,1025.61) |  | 142.17(86.36,201.71) | -0.06(-0.15,0.02) |
| Paraguay | 5894.25(3270.47,9043.08) | 559.99(310.71,859.14) |  | 13583.56(7279.88,22016.16) | 1000.52(536.21,1621.63) |  | 130.45(80.30,195.06) | 0.76(0.45,1.08) |
| Peru | 19844.28(11276.99,30600.68) | 369.20(209.81,569.32) |  | 41286.18(22272.22,68392.28) | 661.97(357.10,1096.57) |  | 108.05(66.29,161.63) | 0.55(-0.04,1.15) |
| Philippines | 101661.55(64182.47,145884.24) | 636.85(402.07,913.88) |  | 214486.03(134631.53,312889.72) | 941.34(590.87,1373.22) |  | 110.98(102.16,120.42) | 0.35(-0.09,0.79) |
| Poland | 20880.20(13048.47,30015.48) | 313.61(195.98,450.82) |  | 20935.83(13159.41,30677.54) | 522.92(328.69,766.24) |  | 0.27(-6.43,7.12) | 0.08(-0.40,0.56) |
| Portugal | 33982.97(20312.87,50148.70) | 2210.31(1321.18,3261.75) |  | 29498.82(16917.09,45966.47) | 3148.46(1805.59,4906.08) |  | -13.20(-33.87,10.56) | -0.02(-0.50,0.46) |
| Puerto Rico | 3786.54(2223.22,5940.11) | 559.66(328.60,877.97) |  | 2632.45(1444.61,4259.27) | 775.92(425.80,1255.43) |  | -30.48(-46.11,-10.99) | -0.11(-0.51,0.28) |
| Qatar | 1117.46(667.48,1671.11) | 1505.24(899.11,2251.01) |  | 5537.72(3084.71,8616.25) | 1788.67(996.35,2783.02) |  | 395.56(278.28,518.28) | 0.11(-0.11,0.32) |
| Republic of Korea | 50848.63(31149.25,75696.35) | 631.54(386.88,940.16) |  | 44206.09(24734.35,70673.76) | 977.02(546.66,1561.99) |  | -13.06(-33.44,11.68) | 1.70(1.04,2.37) |
| Republic of Moldova | 5134.75(3025.38,7716.09) | 637.81(375.79,958.44) |  | 3632.75(2030.23,5854.00) | 986.93(551.56,1590.38) |  | -29.25(-44.03,-9.79) | 0.04(-0.40,0.48) |
| Romania | 19731.08(11560.26,29736.36) | 519.44(304.34,782.84) |  | 18501.32(10088.40,30091.55) | 892.85(486.86,1452.18) |  | -6.23(-26.55,19.21) | 0.41(-0.10,0.93) |
| Russian Federation | 111619.58(70263.28,159996.59) | 483.74(304.51,693.40) |  | 144377.66(90286.79,208982.01) | 781.83(488.92,1131.67) |  | 29.35(24.30,34.90) | -0.12(-0.60,0.37) |
| Rwanda | 19672.21(11934.13,30232.56) | 961.95(583.56,1478.33) |  | 42451.27(24459.18,67639.71) | 1317.56(759.14,2099.33) |  | 115.79(69.75,172.25) | -0.07(-0.37,0.23) |
| Saint Kitts and Nevis | 60.30(34.32,93.79) | 639.00(363.72,993.92) |  | 62.90(33.55,103.20) | 925.69(493.70,1518.75) |  | 4.33(-19.13,32.54) | 0.58(0.36,0.80) |
| Saint Lucia | 208.87(120.33,322.42) | 616.41(355.12,951.52) |  | 223.23(117.61,360.03) | 1070.27(563.85,1726.13) |  | 6.88(-16.91,37.45) | 0.71(0.38,1.04) |
| Saint Vincent and the Grenadines | 173.91(100.53,268.26) | 614.31(355.10,947.60) |  | 170.77(89.98,274.13) | 961.85(506.82,1544.04) |  | -1.80(-24.18,23.12) | 0.49(0.19,0.78) |
| Samoa | 271.88(156.40,420.30) | 594.06(341.73,918.38) |  | 348.67(191.25,558.55) | 687.73(377.22,1101.71) |  | 28.25(-0.56,63.09) | -0.01(-0.19,0.17) |
| San Marino | 52.23(31.31,79.75) | 1794.26(1075.60,2739.87) |  | 83.98(47.77,132.21) | 2633.95(1498.28,4146.75) |  | 60.79(26.59,101.19) | 0.43(-0.02,0.87) |
| Sao Tome and Principe | 282.20(166.77,424.44) | 779.85(460.88,1172.95) |  | 493.95(264.18,784.07) | 933.96(499.51,1482.53) |  | 75.04(34.71,124.23) | 0.18(-0.06,0.42) |
| Saudi Arabia | 59388.76(35664.71,88473.62) | 1436.01(862.37,2139.27) |  | 95603.67(54430.17,146963.01) | 1862.84(1060.57,2863.58) |  | 60.98(24.08,110.39) | 0.68(0.45,0.91) |
| Senegal | 15559.63(9159.01,23305.82) | 711.71(418.94,1066.03) |  | 41079.73(23187.43,66096.17) | 1004.61(567.05,1616.39) |  | 164.01(106.10,231.27) | 0.35(0.04,0.65) |
| Serbia | 7857.18(4428.25,12101.23) | 528.00(297.57,813.19) |  | 7572.44(4295.80,12534.76) | 789.48(447.87,1306.84) |  | -3.62(-26.88,26.13) | 0.10(-0.34,0.54) |
| Seychelles | 112.39(65.35,175.61) | 717.24(417.06,1120.74) |  | 175.36(94.37,285.77) | 1129.00(607.61,1839.88) |  | 56.03(21.35,97.89) | 0.28(-0.08,0.64) |
| Sierra Leone | 7872.19(4696.03,11981.53) | 757.79(452.05,1153.37) |  | 18471.28(10271.46,30017.03) | 827.10(459.93,1344.09) |  | 134.64(85.01,196.47) | 0.39(0.25,0.53) |
| Singapore | 3969.61(2461.15,5702.80) | 891.39(552.66,1280.58) |  | 4266.88(2363.66,6834.74) | 810.94(449.23,1298.98) |  | 7.49(-18.78,38.55) | -0.77(-0.99,-0.55) |
| Slovakia | 4637.54(2713.11,7148.84) | 506.00(296.03,780.01) |  | 4855.35(2648.12,7912.17) | 851.18(464.24,1387.07) |  | 4.70(-17.59,33.03) | 0.05(-0.41,0.51) |
| Slovenia | 1904.62(1086.84,2900.49) | 655.55(374.08,998.32) |  | 1752.71(956.31,2774.20) | 817.62(446.11,1294.13) |  | -7.98(-29.66,20.82) | -0.61(-1.05,-0.17) |
| Solomon Islands | 554.32(321.73,839.50) | 577.12(334.97,874.03) |  | 1148.71(623.18,1831.92) | 697.44(378.36,1112.24) |  | 107.23(59.64,160.72) | 0.03(-0.16,0.23) |
| Somalia | 20273.40(11949.98,31250.55) | 862.47(508.38,1329.46) |  | 77486.06(42623.61,124508.77) | 1249.70(687.44,2008.09) |  | 282.21(197.83,389.21) | 0.22(-0.10,0.55) |
| South Africa | 56704.65(35882.91,80803.89) | 649.15(410.78,925.03) |  | 109731.85(70562.37,156853.78) | 1071.20(688.83,1531.20) |  | 93.51(74.91,113.74) | 0.57(0.15,0.98) |
| South Sudan | 14061.35(8286.33,21328.40) | 875.36(515.85,1327.76) |  | 28618.89(15726.28,46114.66) | 1047.31(575.50,1687.56) |  | 103.53(61.01,159.81) | 0.23(-0.01,0.47) |
| Spain | 146773.69(118979.72,175816.22) | 2551.13(2068.03,3055.93) |  | 158062.15(92843.10,245418.51) | 3406.87(2001.14,5289.74) |  | 7.69(-30.89,59.89) | 1.25(0.66,1.84) |
| Sri Lanka | 29694.81(17144.77,44423.48) | 788.66(455.35,1179.84) |  | 41143.64(22569.21,66899.63) | 1162.78(637.84,1890.68) |  | 38.55(7.21,80.52) | -0.07(-0.47,0.33) |
| Sudan | 81820.50(48568.47,123280.85) | 1511.96(897.50,2278.11) |  | 217230.10(118679.48,340454.40) | 1983.80(1083.81,3109.11) |  | 165.50(106.47,239.79) | 0.32(0.04,0.60) |
| Suriname | 744.45(433.23,1128.09) | 861.00(501.06,1304.71) |  | 1534.34(890.36,2365.50) | 1554.05(901.80,2395.89) |  | 106.10(58.77,164.56) | 0.58(0.21,0.94) |
| Sweden | 13201.81(8422.66,18702.58) | 1345.22(858.24,1905.73) |  | 22103.44(13244.66,32306.99) | 1786.08(1070.24,2610.58) |  | 67.43(36.27,105.17) | 0.22(-0.16,0.60) |
| Switzerland | 11973.98(7208.72,17926.35) | 1581.09(951.87,2367.06) |  | 16669.89(9477.82,25530.48) | 1871.97(1064.32,2866.98) |  | 39.22(8.17,73.36) | -0.12(-0.42,0.18) |
| Syrian Arab Republic | 56476.80(33763.39,84629.54) | 1499.98(896.73,2247.70) |  | 62621.56(35243.97,99573.30) | 2355.75(1325.84,3745.83) |  | 10.88(-13.68,40.29) | 0.72(0.35,1.09) |
| Taiwan (Province of China) | 17012.49(10149.35,26481.26) | 436.03(260.13,678.72) |  | 9492.23(5274.77,15472.58) | 462.02(256.74,753.11) |  | -44.20(-56.76,-30.98) | -0.30(-0.44,-0.17) |
| Tajikistan | 7999.03(4753.39,12020.80) | 581.11(345.32,873.28) |  | 19382.46(10660.26,30158.15) | 863.02(474.66,1342.82) |  | 142.31(84.68,207.26) | 0.37(0.06,0.68) |
| Thailand | 91020.46(52803.35,139186.00) | 780.74(452.93,1193.88) |  | 65743.42(36065.48,106276.03) | 947.41(519.73,1531.52) |  | -27.77(-44.43,-8.11) | -0.08(-0.30,0.13) |
| Timor-Leste | 1324.64(778.81,2010.49) | 687.84(404.41,1043.97) |  | 3403.66(1934.97,5367.09) | 1013.62(576.24,1598.34) |  | 156.95(103.04,224.61) | 0.42(0.15,0.69) |
| Togo | 8227.66(4876.78,12432.78) | 764.48(453.13,1155.21) |  | 19022.34(10613.21,30585.43) | 890.34(496.75,1431.55) |  | 131.20(80.64,187.96) | 0.22(0.05,0.38) |
| Tokelau | 2.22(1.27,3.47) | 546.88(313.65,855.59) |  | 2.26(1.16,3.80) | 774.31(397.01,1305.71) |  | 1.62(-20.22,28.14) | 0.40(0.11,0.69) |
| Tonga | 152.90(89.65,231.22) | 576.28(337.88,871.48) |  | 169.52(89.72,271.64) | 688.70(364.52,1103.60) |  | 10.87(-13.62,40.09) | 0.08(-0.07,0.23) |
| Trinidad and Tobago | 1950.53(1144.43,2962.98) | 714.47(419.20,1085.33) |  | 2484.87(1330.16,3999.77) | 1294.83(693.13,2084.22) |  | 27.39(-4.16,66.97) | 0.16(-0.27,0.59) |
| Tunisia | 39301.23(23266.80,58965.54) | 1928.11(1141.47,2892.84) |  | 68759.91(40072.12,105674.07) | 3669.75(2138.67,5639.88) |  | 74.96(35.06,122.26) | 1.00(0.64,1.36) |
| Turkey | 218183.83(140180.36,309476.58) | 1612.16(1035.79,2286.72) |  | 350082.83(209522.46,527333.92) | 2699.48(1615.62,4066.25) |  | 60.45(12.68,129.74) | 1.40(1.05,1.76) |
| Turkmenistan | 5914.89(3577.72,8833.74) | 646.24(390.89,965.15) |  | 8727.48(4779.57,13874.53) | 886.68(485.59,1409.60) |  | 47.55(15.67,88.06) | 0.29(0.01,0.57) |
| Tuvalu | 11.20(6.42,17.41) | 569.14(326.62,885.22) |  | 18.41(10.08,30.32) | 753.07(412.08,1239.90) |  | 64.48(28.58,105.36) | 0.47(0.34,0.60) |
| Uganda | 62056.71(37572.52,92905.23) | 1285.31(778.20,1924.24) |  | 215530.78(117083.40,337409.60) | 1721.77(935.32,2695.40) |  | 247.31(171.12,339.61) | -1.04(-1.72,-0.36) |
| Ukraine | 48533.35(30383.47,70616.77) | 638.10(399.47,928.44) |  | 47961.76(27280.07,76533.38) | 1009.08(573.95,1610.21) |  | -1.18(-19.95,26.00) | -0.18(-0.67,0.31) |
| United Arab Emirates | 4553.04(2717.53,6766.09) | 1259.69(751.86,1871.97) |  | 16706.65(9691.06,25926.20) | 1843.75(1069.51,2861.22) |  | 266.93(189.83,361.33) | 0.38(0.11,0.64) |
| United Kingdom | 116285.55(74955.67,163220.69) | 1642.68(1058.84,2305.70) |  | 173749.05(112624.46,244491.43) | 2137.55(1385.56,3007.86) |  | 49.42(43.09,55.23) | -0.24(-0.71,0.24) |
| United Republic of Tanzania | 64704.14(38069.99,96900.32) | 890.32(523.84,1333.34) |  | 171495.95(94674.81,271825.72) | 1102.39(608.58,1747.32) |  | 165.05(105.32,237.83) | 0.16(-0.07,0.40) |
| United States of America | 133.47(77.84,205.88) | 637.38(371.72,983.15) |  | 89.70(50.06,142.63) | 947.07(528.51,1505.98) |  | -32.79(-48.78,-15.03) | 0.45(0.15,0.74) |
| United States Virgin Islands | 551069.40(366910.77,758118.99) | 1523.99(1014.70,2096.59) |  | 1501041.86(1076959.77,1995573.81) | 3675.40(2637.01,4886.30) |  | 172.39(140.29,234.26) | 2.62(2.08,3.16) |
| Uruguay | 5684.09(3431.12,8566.18) | 1041.54(628.71,1569.65) |  | 7674.15(4229.30,12433.26) | 1648.51(908.51,2670.84) |  | 35.01(5.96,71.10) | 0.59(0.28,0.90) |
| Uzbekistan | 32688.25(19778.13,48860.38) | 630.73(381.62,942.77) |  | 51050.37(28780.87,79151.08) | 816.05(460.07,1265.24) |  | 56.17(22.95,94.28) | 0.27(0.01,0.52) |
| Vanuatu | 234.44(138.48,355.17) | 571.13(337.35,865.24) |  | 524.44(296.39,840.60) | 704.97(398.41,1129.96) |  | 123.70(76.88,180.98) | 0.08(-0.09,0.25) |
| Venezuela (Bolivarian Republic of) | 23486.20(13598.78,36190.79) | 514.69(298.01,793.11) |  | 28759.74(15896.69,46364.49) | 647.89(358.11,1044.48) |  | 22.45(-6.74,52.10) | 0.34(0.15,0.53) |
| Viet Nam | 93949.07(55604.85,141393.91) | 549.31(325.12,826.72) |  | 127934.28(68519.33,205804.32) | 769.72(412.25,1238.23) |  | 36.17(5.56,74.38) | 0.07(-0.24,0.38) |
| Yemen | 66536.93(39426.29,99719.79) | 1541.00(913.12,2309.52) |  | 155551.91(86738.24,246963.85) | 1711.01(954.09,2716.50) |  | 133.78(81.83,202.82) | 0.09(-0.06,0.24) |
| Zambia | 17291.13(10350.16,26113.76) | 770.08(460.96,1163.00) |  | 56509.79(31533.38,90378.66) | 1056.95(589.79,1690.42) |  | 226.81(158.08,302.59) | 0.15(-0.11,0.42) |
| Zimbabwe | 16275.10(9692.78,24668.88) | 533.12(317.51,808.08) |  | 31267.16(17142.38,49084.61) | 765.58(419.73,1201.84) |  | 92.12(52.24,142.21) | 0.22(-0.06,0.51) |
