# Supplementary material for: Major depressive disorders in children aged 5–14 years: a Global Burden of Disease analysis from the perspective of exercise psychology
Source: Front Public Health. 2025 Oct 29;13:1671222. doi: 10.3389/fpubh.2025.1671222 (PMC12605264; doi:10.3389/fpubh.2025.1671222)
Supplement: Supplementary file 3 [file Table_3.docx]

Table S3: DALYs of major depressive disorders in children between 1990 and 2021 at the national level.

| location | 1990 | |  | 2021 | |  | 1990-2021 | |
| --- | --- | --- | --- | --- | --- | --- | --- | --- |
|  | DALY cases | DALY rate |  | DALY cases | DALY rate |  | Cases change | EAPC |
| Afghanistan | 4970.58(2708.32,8272.95) | 191.63(104.41,318.94) |  | 21607.51(11129.64,35721.91) | 247.82(127.65,409.69) |  | 334.71(229.12,453.73) | 0.57(0.25,0.89) |
| Albania | 381.28(199.83,625.60) | 53.44(28.01,87.68) |  | 280.40(136.30,483.04) | 93.13(45.27,160.44) |  | -26.46(-47.65,2.50) | 0.83(0.49,1.18) |
| Algeria | 12365.00(6526.07,20157.27) | 176.93(93.38,288.43) |  | 18365.35(9088.99,30705.09) | 213.62(105.72,357.16) |  | 48.53(11.21,94.91) | 0.06(-0.20,0.32) |
| American Samoa | 7.26(3.66,11.97) | 63.67(32.09,105.02) |  | 8.70(4.20,15.70) | 83.11(40.07,149.94) |  | 19.89(-15.15,60.64) | 0.37(0.20,0.54) |
| Andorra | 13.26(7.14,22.15) | 195.14(105.11,325.97) |  | 22.46(11.39,38.37) | 293.91(149.04,502.13) |  | 69.41(27.73,128.68) | 0.44(-0.05,0.94) |
| Angola | 3342.74(1712.03,5565.32) | 121.00(61.97,201.45) |  | 14151.51(7073.39,24408.66) | 147.22(73.59,253.93) |  | 323.35(216.74,458.36) | 0.05(-0.22,0.32) |
| Antigua and Barbuda | 8.06(4.06,13.54) | 66.42(33.49,111.64) |  | 11.92(5.75,20.44) | 102.52(49.48,175.76) |  | 47.99(5.84,101.55) | 0.60(0.29,0.91) |
| Argentina | 7621.80(3865.23,12609.11) | 113.78(57.70,188.24) |  | 12844.10(6420.06,21303.10) | 178.71(89.33,296.41) |  | 68.52(22.98,125.64) | 0.70(0.36,1.03) |
| Armenia | 444.93(229.82,730.58) | 67.38(34.81,110.65) |  | 496.21(245.28,874.01) | 122.17(60.39,215.19) |  | 11.53(-22.77,52.93) | 0.65(0.18,1.12) |
| Australia | 5667.17(2998.72,8794.35) | 224.60(118.85,348.54) |  | 8464.14(4566.88,14017.69) | 260.76(140.69,431.85) |  | 49.35(14.41,91.85) | 0.33(0.13,0.53) |
| Austria | 1321.37(684.60,2158.59) | 146.60(75.96,239.49) |  | 1735.23(867.16,3056.08) | 200.63(100.26,353.34) |  | 31.32(-4.90,72.24) | 0.08(-0.25,0.41) |
| Azerbaijan | 1013.15(505.48,1631.18) | 66.52(33.19,107.09) |  | 1768.60(893.15,3017.22) | 107.87(54.48,184.03) |  | 74.56(24.77,140.26) | 0.51(0.10,0.91) |
| Bahamas | 37.24(18.85,62.49) | 67.51(34.17,113.30) |  | 70.51(32.79,124.03) | 117.27(54.54,206.27) |  | 89.37(39.41,151.59) | 0.79(0.35,1.23) |
| Bahrain | 193.92(99.90,323.00) | 190.43(98.10,317.19) |  | 551.07(274.18,952.88) | 271.43(135.05,469.35) |  | 184.18(117.25,275.50) | 0.20(-0.09,0.49) |
| Bangladesh | 24953.65(12982.11,40110.59) | 83.23(43.30,133.79) |  | 35055.86(17078.82,61022.84) | 111.64(54.39,194.33) |  | 40.48(4.03,86.09) | 0.26(-0.06,0.59) |
| Barbados | 30.25(15.46,49.71) | 70.70(36.14,116.20) |  | 38.94(18.87,67.73) | 116.33(56.39,202.33) |  | 28.75(-5.52,76.16) | 0.47(0.10,0.85) |
| Belarus | 1218.68(627.60,1975.88) | 76.26(39.27,123.64) |  | 1577.85(786.84,2654.18) | 142.04(70.83,238.94) |  | 29.47(-8.08,79.22) | 0.15(-0.39,0.68) |
| Belgium | 2010.65(1088.24,3162.84) | 166.22(89.97,261.47) |  | 3119.10(1520.02,5214.47) | 236.26(115.14,394.98) |  | 55.13(12.55,109.57) | 0.44(0.05,0.83) |
| Belize | 34.83(17.62,58.57) | 66.57(33.67,111.95) |  | 88.05(43.92,154.78) | 103.60(51.68,182.13) |  | 152.79(80.91,248.14) | 0.75(0.44,1.06) |
| Benin | 1181.32(618.26,1939.45) | 82.32(43.08,135.15) |  | 3863.34(1873.26,6919.96) | 103.35(50.11,185.12) |  | 227.04(136.28,339.80) | 0.38(0.24,0.53) |
| Bermuda | 6.03(3.06,10.05) | 79.03(40.07,131.82) |  | 6.71(3.20,11.58) | 114.03(54.37,196.79) |  | 11.36(-18.51,49.14) | 0.12(-0.23,0.48) |
| Bhutan | 120.31(63.53,196.00) | 72.16(38.10,117.56) |  | 98.89(48.40,176.98) | 78.40(38.37,140.31) |  | -17.81(-41.23,10.76) | 0.28(0.14,0.42) |
| Bolivia (Plurinational State of) | 888.68(429.86,1492.89) | 53.03(25.65,89.08) |  | 2283.15(1120.56,3849.72) | 99.60(48.88,167.94) |  | 156.92(82.47,252.38) | 0.72(0.11,1.34) |
| Bosnia and Herzegovina | 570.04(284.79,931.07) | 76.62(38.28,125.14) |  | 362.03(172.07,615.59) | 106.67(50.70,181.37) |  | -36.49(-55.38,-10.78) | -0.39(-0.82,0.05) |
| Botswana | 331.16(163.83,544.19) | 87.74(43.41,144.19) |  | 662.16(330.23,1129.23) | 143.08(71.36,244.01) |  | 99.95(49.61,174.75) | 0.33(0.02,0.65) |
| Brazil | 23597.99(12970.09,36677.08) | 66.51(36.55,103.37) |  | 26723.02(14677.37,42727.66) | 84.49(46.41,135.10) |  | 13.24(1.91,26.05) | -1.02(-2.10,0.08) |
| Brunei Darussalam | 39.66(20.70,62.84) | 70.70(36.91,112.03) |  | 60.38(28.62,108.22) | 94.79(44.93,169.88) |  | 52.25(10.72,105.76) | 0.54(0.34,0.74) |
| Bulgaria | 818.03(435.26,1356.66) | 68.20(36.29,113.11) |  | 805.00(381.82,1335.33) | 119.55(56.70,198.30) |  | -1.59(-29.60,34.33) | -0.19(-0.83,0.46) |
| Burkina Faso | 2538.89(1325.46,4066.77) | 89.32(46.63,143.07) |  | 6236.78(3126.22,10425.54) | 99.54(49.89,166.39) |  | 145.65(80.14,226.93) | 0.28(0.14,0.42) |
| Burundi | 1571.69(819.70,2584.39) | 101.66(53.02,167.16) |  | 4905.14(2358.47,8482.37) | 132.79(63.85,229.64) |  | 212.09(124.93,311.80) | 0.01(-0.27,0.28) |
| Cabo Verde | 90.23(45.49,150.44) | 92.25(46.50,153.79) |  | 146.84(71.17,250.58) | 148.11(71.79,252.75) |  | 62.73(22.47,117.87) | 0.70(0.38,1.02) |
| Cambodia | 2347.05(1147.12,3909.41) | 82.76(40.45,137.85) |  | 4174.30(2017.32,7226.28) | 123.94(59.90,214.56) |  | 77.85(28.58,140.49) | 0.29(0.01,0.56) |
| Cameroon | 2556.40(1288.41,4190.82) | 88.90(44.80,145.74) |  | 9212.09(4488.79,15875.96) | 107.08(52.18,184.54) |  | 260.35(164.24,381.25) | 0.24(0.09,0.40) |
| Canada | 5340.37(2881.00,8534.92) | 139.56(75.29,223.04) |  | 9486.30(4689.67,16354.30) | 222.07(109.78,382.85) |  | 77.63(27.44,134.98) | 0.40(0.06,0.75) |
| Central African Republic | 827.58(407.78,1374.15) | 115.10(56.72,191.13) |  | 2007.61(1000.63,3409.27) | 138.94(69.25,235.94) |  | 142.59(79.85,220.06) | 0.38(0.16,0.60) |
| Chad | 1810.49(925.62,3028.34) | 107.55(54.98,179.89) |  | 6157.51(3120.99,10984.36) | 114.47(58.02,204.19) |  | 240.10(151.13,362.52) | 0.16(0.01,0.30) |
| Chile | 4928.30(2687.56,7884.64) | 194.42(106.02,311.05) |  | 6920.64(3536.06,11721.23) | 269.93(137.92,457.17) |  | 40.43(-0.22,88.27) | 0.21(-0.18,0.60) |
| China | 104277.09(59458.85,161878.46) | 50.48(28.78,78.36) |  | 72403.13(39826.00,113182.57) | 39.79(21.89,62.20) |  | -30.57(-38.23,-22.32) | -0.33(-0.80,0.13) |
| Colombia | 3509.58(1756.43,5844.22) | 47.07(23.56,78.38) |  | 4669.62(2236.75,8252.86) | 65.12(31.19,115.10) |  | 33.05(-7.23,88.30) | 0.26(-0.05,0.58) |
| Comoros | 115.99(62.08,193.06) | 89.03(47.66,148.19) |  | 194.04(97.03,338.13) | 122.16(61.08,212.87) |  | 67.29(23.18,124.42) | 0.39(0.13,0.64) |
| Congo | 812.18(409.20,1389.31) | 123.34(62.14,210.99) |  | 2011.28(969.44,3401.36) | 155.14(74.78,262.36) |  | 147.64(77.80,228.27) | 0.05(-0.23,0.33) |
| Cook Islands | 3.15(1.56,5.36) | 72.28(35.92,122.98) |  | 2.53(1.22,4.52) | 95.19(45.94,169.83) |  | -19.47(-42.16,12.84) | 0.44(0.24,0.64) |
| Costa Rica | 415.54(202.74,697.09) | 57.86(28.23,97.06) |  | 649.43(307.84,1098.91) | 91.60(43.42,155.00) |  | 56.29(11.39,114.88) | 0.60(0.29,0.91) |
| Croatia | 484.95(252.45,801.44) | 70.72(36.81,116.87) |  | 416.02(205.65,704.48) | 100.27(49.57,169.80) |  | -14.21(-39.27,18.52) | -0.04(-0.42,0.35) |
| Cuba | 1415.04(712.27,2383.99) | 87.95(44.27,148.17) |  | 1345.28(689.40,2328.88) | 109.15(55.93,188.95) |  | -4.93(-30.47,31.77) | -0.35(-0.70,0.00) |
| Cyprus | 234.02(120.29,392.07) | 174.60(89.74,292.51) |  | 334.12(163.64,561.70) | 232.58(113.91,391.00) |  | 42.77(7.54,83.66) | 0.21(-0.04,0.46) |
| Czechia | 1108.91(567.95,1861.54) | 71.32(36.53,119.72) |  | 1177.31(566.35,1999.89) | 102.00(49.07,173.26) |  | 6.17(-24.58,42.22) | -0.21(-0.70,0.28) |
| C么te d'Ivoire | 2597.93(1340.22,4220.61) | 76.57(39.50,124.40) |  | 6246.06(3043.91,10547.96) | 86.54(42.17,146.15) |  | 140.42(73.14,214.18) | 0.09(-0.06,0.23) |
| Democratic People's Republic of Korea | 1979.65(965.48,3210.93) | 54.79(26.72,88.87) |  | 1960.87(919.97,3348.97) | 60.14(28.21,102.71) |  | -0.95(-31.14,41.09) | 0.13(0.02,0.25) |
| Democratic Republic of the Congo | 11830.98(5862.16,19654.67) | 113.67(56.32,188.84) |  | 33339.66(16370.76,56489.53) | 136.42(66.99,231.15) |  | 181.80(104.19,278.22) | 0.26(0.05,0.48) |
| Denmark | 1054.73(554.05,1715.59) | 177.64(93.31,288.94) |  | 1475.07(745.77,2569.63) | 229.24(115.90,399.34) |  | 39.85(4.18,85.50) | 0.58(0.27,0.89) |
| Djibouti | 110.09(57.47,177.36) | 100.56(52.49,162.00) |  | 338.63(160.15,570.00) | 126.60(59.88,213.10) |  | 207.58(133.83,303.46) | 0.13(-0.13,0.39) |
| Dominica | 10.93(5.36,18.08) | 67.67(33.19,111.96) |  | 11.11(5.36,18.93) | 108.84(52.55,185.48) |  | 1.61(-26.06,38.49) | 0.55(0.21,0.90) |
| Dominican Republic | 1349.83(693.98,2186.28) | 79.42(40.83,128.63) |  | 1968.41(957.75,3263.80) | 103.31(50.27,171.30) |  | 45.83(7.47,94.20) | 0.41(0.13,0.70) |
| Ecuador | 1751.06(885.32,2912.31) | 69.49(35.14,115.58) |  | 4026.87(2040.68,6889.62) | 118.10(59.85,202.06) |  | 129.97(59.41,215.50) | 0.81(0.33,1.28) |
| Egypt | 21763.76(10833.89,35489.50) | 159.45(79.38,260.02) |  | 51885.89(26462.01,89059.63) | 217.85(111.10,373.92) |  | 138.40(75.74,214.77) | 0.33(0.05,0.60) |
| El Salvador | 1051.37(562.52,1701.05) | 75.75(40.53,122.55) |  | 1095.46(490.16,1914.25) | 89.93(40.24,157.15) |  | 4.19(-26.90,44.79) | -0.01(-0.33,0.31) |
| Equatorial Guinea | 133.84(67.65,215.87) | 116.73(59.00,188.28) |  | 585.29(304.42,1013.64) | 147.18(76.55,254.90) |  | 337.31(219.75,484.95) | 0.37(0.14,0.60) |
| Eritrea | 967.99(493.46,1571.94) | 99.96(50.96,162.33) |  | 1964.16(955.97,3262.96) | 122.26(59.50,203.10) |  | 102.91(48.03,171.93) | 0.19(0.03,0.35) |
| Estonia | 215.73(111.49,346.51) | 94.41(48.79,151.65) |  | 205.98(102.10,351.85) | 140.16(69.48,239.42) |  | -4.52(-33.56,31.66) | -0.58(-1.10,-0.05) |
| Eswatini | 205.27(107.13,335.23) | 85.42(44.58,139.49) |  | 487.99(236.16,909.08) | 179.27(86.75,333.95) |  | 137.73(76.26,219.99) | 0.73(0.24,1.22) |
| Ethiopia | 12668.34(7114.27,20094.74) | 86.04(48.32,136.47) |  | 30019.16(16872.07,47565.26) | 105.76(59.44,167.57) |  | 136.96(105.10,172.95) | 0.02(-0.30,0.33) |
| Fiji | 124.09(64.37,206.61) | 66.29(34.39,110.37) |  | 172.46(83.33,306.43) | 95.03(45.92,168.85) |  | 38.98(-1.47,91.09) | 0.20(-0.05,0.46) |
| Finland | 1881.68(1004.98,3055.99) | 288.48(154.08,468.52) |  | 1704.78(860.01,2869.32) | 282.11(142.32,474.82) |  | -9.40(-31.00,23.03) | -1.13(-1.65,-0.62) |
| France | 16771.37(9415.77,26965.38) | 214.34(120.33,344.61) |  | 22170.87(11607.73,36825.50) | 274.61(143.77,456.12) |  | 32.19(-5.86,75.27) | -0.17(-0.62,0.28) |
| Gabon | 302.90(150.73,489.82) | 120.53(59.98,194.91) |  | 695.12(352.35,1213.64) | 163.33(82.79,285.16) |  | 129.49(74.75,206.43) | 0.37(0.14,0.60) |
| Gambia | 337.84(183.67,559.16) | 122.59(66.65,202.90) |  | 1002.45(500.26,1656.11) | 157.65(78.67,260.45) |  | 196.72(122.25,299.26) | 0.32(0.10,0.54) |
| Georgia | 677.17(346.11,1106.79) | 75.23(38.45,122.96) |  | 595.02(302.83,1045.83) | 120.78(61.47,212.29) |  | -12.13(-34.01,19.15) | 0.37(0.00,0.74) |
| Germany | 13243.31(7368.33,20977.58) | 156.42(87.03,247.77) |  | 19531.21(9923.11,33484.59) | 246.57(125.27,422.72) |  | 47.48(1.18,103.95) | 0.63(-0.05,1.31) |
| Ghana | 3758.70(1944.03,6112.60) | 92.07(47.62,149.72) |  | 9475.10(4546.11,16256.57) | 114.95(55.15,197.22) |  | 152.08(86.37,228.85) | 0.32(0.13,0.51) |
| Greece | 3972.65(2061.07,6509.44) | 270.51(140.34,443.24) |  | 4050.58(2034.61,6834.07) | 416.67(209.29,702.99) |  | 1.96(-21.47,29.44) | 0.17(-0.22,0.56) |
| Greenland | 23.60(13.54,38.06) | 270.77(155.31,436.69) |  | 32.36(16.86,55.65) | 419.69(218.64,721.73) |  | 37.11(3.58,80.30) | 0.44(0.11,0.78) |
| Grenada | 14.32(6.98,24.23) | 66.94(32.63,113.25) |  | 16.04(7.42,28.10) | 107.24(49.62,187.82) |  | 12.02(-18.85,53.69) | 0.58(0.29,0.88) |
| Guam | 18.43(9.35,31.31) | 72.18(36.63,122.64) |  | 26.34(13.43,45.59) | 110.68(56.44,191.58) |  | 42.93(5.24,91.33) | 0.68(0.41,0.95) |
| Guatemala | 1627.77(829.45,2681.41) | 64.41(32.82,106.10) |  | 3426.63(1648.44,5789.62) | 101.54(48.85,171.56) |  | 110.51(53.24,195.26) | 0.73(0.42,1.04) |
| Guinea | 1348.57(686.49,2215.75) | 84.75(43.14,139.24) |  | 4103.82(2011.24,7299.15) | 108.13(52.99,192.32) |  | 204.31(120.17,322.49) | 0.36(0.16,0.56) |
| Guinea-Bissau | 256.71(132.11,431.32) | 87.22(44.89,146.55) |  | 610.88(308.61,1062.32) | 108.03(54.57,187.86) |  | 137.97(73.26,225.61) | 0.22(0.02,0.43) |
| Guyana | 163.99(85.95,270.98) | 90.54(47.45,149.60) |  | 212.04(102.74,365.19) | 152.68(73.98,262.96) |  | 29.30(-5.15,71.35) | 1.12(0.78,1.46) |
| Haiti | 1153.88(616.00,1899.26) | 69.83(37.28,114.94) |  | 2661.05(1350.20,4456.02) | 95.62(48.52,160.12) |  | 130.62(70.84,213.38) | 0.23(-0.03,0.48) |
| Honduras | 732.02(357.33,1210.75) | 52.80(25.77,87.33) |  | 2016.25(921.30,3407.25) | 92.42(42.23,156.18) |  | 175.44(102.56,278.33) | 0.77(0.36,1.18) |
| Hungary | 1044.73(533.25,1738.32) | 69.10(35.27,114.98) |  | 833.26(412.87,1434.94) | 89.20(44.20,153.62) |  | -20.24(-43.89,11.14) | -0.18(-0.54,0.19) |
| Iceland | 67.22(35.25,107.51) | 158.88(83.32,254.11) |  | 87.15(45.62,149.43) | 191.26(100.12,327.95) |  | 29.65(-2.58,70.93) | 0.11(-0.14,0.36) |
| India | 95141.91(52856.57,152174.11) | 45.25(25.14,72.37) |  | 236202.04(131292.45,378597.02) | 92.61(51.47,148.43) |  | 148.26(130.56,170.42) | 1.93(1.56,2.30) |
| Indonesia | 26798.13(14989.75,42296.48) | 59.01(33.01,93.13) |  | 42512.01(23802.08,65456.41) | 93.68(52.45,144.23) |  | 58.64(45.72,73.69) | 0.42(0.05,0.80) |
| Iran (Islamic Republic of) | 23002.07(13074.66,36188.07) | 138.46(78.70,217.84) |  | 29589.34(16735.98,46780.41) | 210.99(119.34,333.57) |  | 28.64(20.36,37.70) | 0.49(0.16,0.82) |
| Iraq | 7527.99(3854.39,12513.12) | 147.79(75.67,245.66) |  | 16712.51(8650.80,29396.13) | 182.28(94.35,320.62) |  | 122.00(65.99,192.07) | 0.46(-0.02,0.93) |
| Ireland | 1385.25(739.90,2259.76) | 199.74(106.69,325.83) |  | 2097.79(1093.02,3577.10) | 300.20(156.41,511.89) |  | 51.44(16.33,97.56) | 0.05(-0.37,0.47) |
| Israel | 2238.59(1183.24,3705.64) | 220.18(116.38,364.48) |  | 4632.46(2365.31,7669.84) | 270.99(138.37,448.67) |  | 106.94(60.59,174.63) | 0.04(-0.27,0.34) |
| Italy | 11096.28(6411.76,17459.18) | 171.13(98.89,269.26) |  | 13559.49(7834.46,21295.05) | 249.73(144.29,392.19) |  | 22.20(11.51,33.09) | 0.29(-0.20,0.78) |
| Jamaica | 377.79(192.18,628.42) | 67.92(34.55,112.98) |  | 449.91(211.51,773.41) | 109.00(51.24,187.38) |  | 19.09(-14.75,61.41) | 0.64(0.33,0.96) |
| Japan | 10700.99(6200.27,16610.18) | 65.13(37.74,101.09) |  | 9865.14(5501.87,15012.17) | 90.85(50.67,138.25) |  | -7.81(-14.74,-0.19) | 0.37(0.04,0.70) |
| Jordan | 1839.82(947.68,3036.05) | 178.07(91.72,293.86) |  | 5987.30(3164.48,10269.70) | 236.12(124.80,405.00) |  | 225.43(148.48,321.67) | 0.41(0.19,0.64) |
| Kazakhstan | 2677.09(1403.45,4266.35) | 80.80(42.36,128.76) |  | 3688.20(1859.77,6278.23) | 106.04(53.47,180.51) |  | 37.77(-2.62,81.19) | 0.05(-0.31,0.42) |
| Kenya | 5585.97(3210.51,8549.92) | 81.22(46.68,124.32) |  | 13303.81(7639.49,20565.66) | 104.63(60.08,161.74) |  | 138.16(125.13,153.89) | 0.26(-0.01,0.54) |
| Kiribati | 11.61(5.77,18.97) | 65.38(32.50,106.83) |  | 22.66(10.92,40.18) | 81.72(39.40,144.93) |  | 95.18(38.33,163.48) | 0.11(-0.07,0.28) |
| Kuwait | 619.58(329.84,1025.12) | 177.08(94.27,292.98) |  | 1149.78(561.42,1927.28) | 198.08(96.72,332.02) |  | 85.58(37.85,144.40) | 0.27(0.06,0.47) |
| Kyrgyzstan | 837.63(462.79,1385.50) | 80.94(44.72,133.89) |  | 1716.40(821.54,2919.57) | 115.93(55.49,197.20) |  | 104.91(49.76,181.47) | 0.24(-0.12,0.60) |
| Lao People's Democratic Republic | 849.13(430.86,1380.02) | 75.00(38.06,121.89) |  | 1547.71(745.27,2606.15) | 105.51(50.81,177.67) |  | 82.27(32.16,147.90) | 0.57(0.35,0.79) |
| Latvia | 290.11(150.11,475.42) | 79.20(40.98,129.78) |  | 281.61(141.63,488.13) | 138.49(69.65,240.05) |  | -2.93(-29.51,31.85) | -0.07(-0.61,0.47) |
| Lebanon | 1147.45(606.69,1917.57) | 174.85(92.45,292.19) |  | 2989.19(1515.77,5109.47) | 342.74(173.80,585.84) |  | 160.51(91.23,246.93) | 0.52(0.10,0.94) |
| Lesotho | 593.79(310.19,965.51) | 136.10(71.10,221.30) |  | 1033.81(509.48,1735.54) | 242.24(119.38,406.67) |  | 74.10(27.17,126.75) | 0.61(0.18,1.04) |
| Liberia | 614.53(328.78,1006.38) | 92.31(49.38,151.16) |  | 1609.97(786.12,2872.79) | 113.41(55.38,202.37) |  | 161.98(95.95,264.74) | 0.68(0.44,0.92) |
| Libya | 2217.58(1151.53,3642.24) | 188.89(98.09,310.24) |  | 2653.87(1355.90,4519.76) | 248.36(126.89,422.98) |  | 19.67(-6.68,50.87) | 0.42(0.16,0.69) |
| Lithuania | 449.83(232.77,722.60) | 83.05(42.98,133.42) |  | 405.13(202.11,724.52) | 146.68(73.17,262.32) |  | -9.94(-36.26,22.84) | 0.20(-0.30,0.69) |
| Luxembourg | 75.38(37.98,123.77) | 174.60(87.96,286.68) |  | 153.22(78.42,256.53) | 224.78(115.04,376.34) |  | 103.25(55.24,170.86) | 0.15(-0.20,0.49) |
| Madagascar | 3293.54(1723.45,5411.96) | 99.79(52.22,163.98) |  | 10147.77(5027.38,17734.76) | 132.73(65.76,231.96) |  | 208.11(119.95,316.19) | 0.32(0.06,0.59) |
| Malawi | 2272.53(1182.53,3694.54) | 85.73(44.61,139.37) |  | 6751.29(3334.60,11295.89) | 125.03(61.75,209.19) |  | 197.08(118.58,299.50) | 0.36(0.04,0.68) |
| Malaysia | 4029.87(2134.24,6557.42) | 96.16(50.93,156.48) |  | 7690.71(3786.86,13916.58) | 149.18(73.46,269.95) |  | 90.84(38.32,151.35) | 3.47(2.42,4.53) |
| Maldives | 56.77(28.46,91.90) | 89.74(44.99,145.27) |  | 81.75(40.75,136.49) | 119.50(59.56,199.52) |  | 44.01(4.46,96.40) | -0.11(-0.42,0.20) |
| Mali | 1680.74(849.11,2706.53) | 70.01(35.37,112.74) |  | 5766.91(2782.16,10128.11) | 82.44(39.77,144.78) |  | 243.12(148.77,370.16) | 0.25(0.03,0.46) |
| Malta | 106.17(54.92,175.66) | 179.11(92.64,296.33) |  | 94.47(45.67,162.82) | 224.87(108.71,387.56) |  | -11.02(-32.15,18.27) | 0.23(-0.00,0.47) |
| Marshall Islands | 9.41(4.94,15.07) | 64.88(34.03,103.92) |  | 9.82(4.72,16.73) | 83.29(40.05,142.01) |  | 4.29(-23.09,44.80) | 0.12(-0.08,0.31) |
| Mauritania | 417.79(215.40,700.15) | 75.49(38.92,126.51) |  | 1112.67(534.21,1956.37) | 93.03(44.67,163.57) |  | 166.32(86.85,266.22) | 0.26(0.02,0.51) |
| Mauritius | 360.58(186.58,596.86) | 160.56(83.08,265.76) |  | 314.67(155.51,544.66) | 219.88(108.67,380.59) |  | -12.73(-34.79,16.46) | 0.21(-0.16,0.58) |
| Mexico | 10910.00(6202.91,16805.89) | 50.47(28.69,77.74) |  | 20306.80(11257.67,31829.70) | 91.51(50.73,143.44) |  | 86.13(68.83,103.08) | 0.84(0.41,1.26) |
| Micronesia (Federated States of) | 20.50(10.37,34.46) | 67.72(34.28,113.86) |  | 18.04(8.85,31.53) | 85.37(41.87,149.20) |  | -11.99(-36.81,24.24) | 0.24(0.08,0.40) |
| Monaco | 4.60(2.43,7.63) | 195.38(103.16,324.24) |  | 9.77(4.72,16.98) | 290.76(140.34,505.12) |  | 112.49(61.72,175.22) | 0.53(0.26,0.80) |
| Mongolia | 511.58(276.23,818.70) | 91.28(49.29,146.07) |  | 623.51(315.05,1062.77) | 89.60(45.27,152.72) |  | 21.88(-11.02,62.32) | -0.38(-0.67,-0.09) |
| Montenegro | 66.61(34.19,108.92) | 61.04(31.33,99.80) |  | 78.54(37.71,136.58) | 104.41(50.14,181.57) |  | 17.90(-14.62,61.67) | 0.37(-0.06,0.81) |
| Morocco | 11873.89(6241.39,19586.75) | 190.73(100.26,314.63) |  | 17725.00(9310.62,31273.70) | 270.98(142.34,478.11) |  | 49.28(10.38,88.96) | 0.45(0.10,0.80) |
| Mozambique | 3730.42(1949.21,6089.08) | 98.29(51.36,160.44) |  | 12330.34(5866.95,20975.48) | 135.70(64.57,230.83) |  | 230.54(138.45,349.05) | 0.23(-0.06,0.52) |
| Myanmar | 5616.31(2705.81,9570.99) | 57.70(27.80,98.32) |  | 10357.50(5080.25,17422.64) | 99.70(48.90,167.71) |  | 84.42(33.73,153.57) | 0.46(0.00,0.91) |
| Namibia | 288.95(148.29,472.61) | 77.18(39.61,126.24) |  | 781.41(368.56,1347.15) | 142.86(67.38,246.29) |  | 170.43(93.81,256.12) | 0.46(0.02,0.91) |
| Nauru | 1.71(0.82,2.90) | 66.01(31.60,112.04) |  | 2.25(1.10,4.08) | 87.37(42.67,158.22) |  | 32.04(-3.12,79.86) | 0.31(0.15,0.46) |
| Nepal | 4060.49(2057.01,6937.41) | 79.12(40.08,135.18) |  | 8143.80(4108.11,14130.70) | 133.05(67.12,230.86) |  | 100.56(47.44,169.29) | 0.88(0.53,1.22) |
| Netherlands | 2419.48(1320.52,3961.36) | 135.25(73.82,221.44) |  | 4878.18(2488.17,8301.54) | 267.92(136.65,455.93) |  | 101.62(46.12,171.37) | 1.77(1.40,2.14) |
| New Zealand | 627.55(340.40,998.76) | 120.57(65.40,191.89) |  | 1300.25(709.86,2151.15) | 194.32(106.09,321.49) |  | 107.19(64.37,170.08) | 2.07(1.62,2.52) |
| Nicaragua | 757.78(374.77,1263.38) | 65.46(32.37,109.13) |  | 1252.75(607.95,2192.22) | 94.20(45.71,164.84) |  | 65.32(16.94,128.89) | 0.36(0.05,0.67) |
| Niger | 2000.81(1019.26,3296.35) | 84.21(42.90,138.74) |  | 6725.13(3440.25,11771.27) | 87.71(44.87,153.52) |  | 236.12(149.08,337.95) | 0.07(-0.10,0.23) |
| Nigeria | 15887.24(8823.19,25128.57) | 68.39(37.98,108.18) |  | 47559.21(26369.80,73861.09) | 73.77(40.90,114.56) |  | 199.35(177.40,222.52) | 0.12(-0.20,0.45) |
| Niue | 0.37(0.19,0.63) | 67.00(34.41,113.80) |  | 0.25(0.11,0.43) | 91.09(42.22,160.68) |  | -33.53(-51.03,-8.88) | 0.29(0.12,0.46) |
| North Macedonia | 203.14(102.26,338.90) | 56.99(28.69,95.08) |  | 246.84(117.22,449.03) | 108.71(51.62,197.75) |  | 21.52(-12.26,71.27) | 0.60(0.10,1.11) |
| Northern Mariana Islands | 4.98(2.53,8.31) | 67.30(34.22,112.22) |  | 8.62(4.01,14.95) | 106.97(49.79,185.54) |  | 72.91(22.64,132.42) | 0.94(0.59,1.30) |
| Norway | 828.08(483.57,1292.40) | 158.49(92.55,247.35) |  | 1273.80(722.86,2000.88) | 198.20(112.48,311.33) |  | 53.83(37.30,70.57) | 0.64(0.32,0.96) |
| Oman | 874.02(457.70,1400.97) | 170.47(89.27,273.26) |  | 1844.48(886.42,3146.69) | 230.85(110.94,393.83) |  | 111.03(56.57,174.83) | 0.20(-0.09,0.49) |
| Pakistan | 17900.88(10061.56,28889.73) | 58.16(32.69,93.86) |  | 39684.57(21524.71,63758.84) | 71.23(38.63,114.44) |  | 121.69(83.95,171.30) | 0.26(0.00,0.52) |
| Palau | 2.26(1.16,3.92) | 73.35(37.58,127.29) |  | 2.19(1.07,3.84) | 94.67(46.47,166.09) |  | -3.03(-32.13,36.74) | 0.32(0.14,0.50) |
| Palestine | 1492.04(797.46,2473.06) | 258.01(137.90,427.65) |  | 4616.01(2380.90,7736.27) | 367.98(189.80,616.72) |  | 209.38(136.83,301.67) | 0.52(0.26,0.78) |
| Panama | 309.76(156.62,529.01) | 56.49(28.56,96.47) |  | 666.31(318.21,1132.00) | 85.19(40.69,144.73) |  | 115.10(51.81,191.78) | 0.59(0.20,0.98) |
| Papua New Guinea | 713.22(361.33,1174.84) | 67.77(34.33,111.64) |  | 1748.85(846.13,3031.97) | 73.00(35.32,126.56) |  | 145.20(72.70,232.57) | -0.02(-0.11,0.06) |
| Paraguay | 671.75(338.06,1137.67) | 63.82(32.12,108.09) |  | 1535.07(734.73,2669.82) | 113.07(54.12,196.65) |  | 128.52(69.32,220.74) | 0.76(0.45,1.07) |
| Peru | 2282.82(1162.66,4022.35) | 42.47(21.63,74.84) |  | 4755.88(2428.49,8018.53) | 76.25(38.94,128.57) |  | 108.33(49.21,190.28) | 0.58(-0.01,1.17) |
| Philippines | 11557.41(6354.10,18167.18) | 72.40(39.80,113.81) |  | 24535.04(13516.25,38082.44) | 107.68(59.32,167.14) |  | 112.29(100.10,125.45) | 0.37(-0.06,0.81) |
| Poland | 2426.13(1306.30,3868.97) | 36.44(19.62,58.11) |  | 2428.32(1313.37,3853.43) | 60.65(32.80,96.25) |  | 0.09(-10.19,12.81) | 0.08(-0.40,0.55) |
| Portugal | 4053.41(2147.33,6550.80) | 263.64(139.67,426.07) |  | 3543.18(1786.01,6010.40) | 378.17(190.62,641.50) |  | -12.59(-35.70,13.73) | 0.02(-0.47,0.50) |
| Puerto Rico | 434.34(227.86,727.13) | 64.20(33.68,107.47) |  | 301.16(148.56,524.95) | 88.77(43.79,154.73) |  | -30.66(-51.41,-5.46) | -0.09(-0.48,0.30) |
| Qatar | 131.69(69.36,217.79) | 177.38(93.43,293.36) |  | 655.63(312.55,1131.35) | 211.77(100.95,365.42) |  | 397.87(275.65,545.48) | 0.11(-0.10,0.33) |
| Republic of Korea | 5951.94(3110.48,9701.60) | 73.92(38.63,120.49) |  | 5136.72(2592.49,8927.58) | 113.53(57.30,197.31) |  | -13.70(-37.65,18.74) | 1.66(0.99,2.34) |
| Republic of Moldova | 597.31(297.50,995.15) | 74.19(36.95,123.61) |  | 426.68(217.50,739.31) | 115.92(59.09,200.85) |  | -28.57(-46.15,-2.48) | 0.08(-0.36,0.52) |
| Romania | 2321.49(1180.28,3916.28) | 61.12(31.07,103.10) |  | 2145.46(1031.99,3497.98) | 103.54(49.80,168.81) |  | -7.58(-34.68,23.90) | 0.41(-0.09,0.92) |
| Russian Federation | 12851.66(7077.99,20409.57) | 55.70(30.67,88.45) |  | 16617.20(9118.99,26612.55) | 89.98(49.38,144.11) |  | 29.30(20.01,38.63) | -0.11(-0.59,0.38) |
| Rwanda | 2233.17(1207.23,3731.42) | 109.20(59.03,182.46) |  | 4907.24(2449.24,8601.42) | 152.31(76.02,266.96) |  | 119.74(63.11,188.12) | 0.00(-0.29,0.29) |
| Saint Kitts and Nevis | 6.85(3.40,11.38) | 72.56(36.06,120.57) |  | 7.14(3.50,12.52) | 105.07(51.56,184.19) |  | 4.28(-25.79,40.55) | 0.63(0.42,0.84) |
| Saint Lucia | 23.73(12.30,39.54) | 70.03(36.29,116.70) |  | 25.41(12.29,43.73) | 121.83(58.94,209.65) |  | 7.08(-23.47,45.20) | 0.71(0.38,1.04) |
| Saint Vincent and the Grenadines | 19.90(10.33,32.87) | 70.31(36.47,116.10) |  | 19.50(8.88,33.56) | 109.82(50.00,189.05) |  | -2.04(-30.68,29.80) | 0.48(0.19,0.78) |
| Samoa | 31.35(15.41,51.70) | 68.50(33.66,112.96) |  | 40.22(19.23,68.33) | 79.32(37.94,134.78) |  | 28.29(-6.39,73.25) | -0.02(-0.19,0.16) |
| San Marino | 6.27(3.24,10.31) | 215.24(111.38,354.20) |  | 10.05(4.96,16.95) | 315.36(155.43,531.52) |  | 60.48(24.04,108.36) | 0.42(-0.03,0.87) |
| Sao Tome and Principe | 32.33(16.16,53.49) | 89.33(44.65,147.81) |  | 57.15(27.50,98.14) | 108.06(52.00,185.57) |  | 76.79(31.52,145.14) | 0.22(-0.02,0.46) |
| Saudi Arabia | 6987.93(3649.12,11567.14) | 168.97(88.24,279.69) |  | 11257.62(5776.67,18359.45) | 219.35(112.56,357.73) |  | 61.10(20.93,117.92) | 0.69(0.46,0.91) |
| Senegal | 1770.14(909.45,2894.29) | 80.97(41.60,132.39) |  | 4715.56(2375.04,7998.66) | 115.32(58.08,195.61) |  | 166.39(97.16,248.96) | 0.38(0.08,0.69) |
| Serbia | 917.36(450.72,1509.34) | 61.65(30.29,101.43) |  | 891.54(428.51,1602.70) | 92.95(44.67,167.09) |  | -2.82(-30.82,35.67) | 0.12(-0.31,0.57) |
| Seychelles | 12.99(6.85,21.82) | 82.93(43.71,139.27) |  | 20.24(9.86,34.64) | 130.33(63.50,223.04) |  | 55.77(14.86,109.53) | 0.27(-0.08,0.63) |
| Sierra Leone | 896.48(461.43,1485.03) | 86.30(44.42,142.95) |  | 2124.55(1049.14,3693.47) | 95.13(46.98,165.38) |  | 136.99(71.03,217.10) | 0.43(0.29,0.57) |
| Singapore | 460.86(252.02,738.40) | 103.49(56.59,165.81) |  | 496.36(245.04,847.59) | 94.34(46.57,161.09) |  | 7.70(-25.14,45.81) | -0.75(-0.97,-0.53) |
| Slovakia | 544.14(275.39,892.96) | 59.37(30.05,97.43) |  | 563.85(265.06,1007.51) | 98.85(46.47,176.62) |  | 3.62(-28.34,45.35) | 0.06(-0.39,0.52) |
| Slovenia | 221.27(110.40,372.81) | 76.16(38.00,128.32) |  | 203.21(97.40,340.69) | 94.80(45.44,158.93) |  | -8.16(-34.90,24.53) | -0.58(-1.02,-0.15) |
| Solomon Islands | 63.43(31.36,103.23) | 66.04(32.65,107.47) |  | 132.05(63.67,230.15) | 80.17(38.66,139.73) |  | 108.20(44.51,188.36) | 0.05(-0.14,0.24) |
| Somalia | 2320.81(1217.34,3824.84) | 98.73(51.79,162.72) |  | 8880.95(4556.41,14842.50) | 143.23(73.49,239.38) |  | 282.67(179.69,411.98) | 0.24(-0.08,0.57) |
| South Africa | 6429.25(3632.97,10242.94) | 73.60(41.59,117.26) |  | 12465.43(7010.77,19402.09) | 121.69(68.44,189.40) |  | 93.89(72.20,119.70) | 0.57(0.16,0.99) |
| South Sudan | 1599.21(806.88,2691.69) | 99.56(50.23,167.57) |  | 3269.14(1606.42,5604.12) | 119.63(58.79,205.08) |  | 104.42(51.77,177.09) | 0.25(0.01,0.49) |
| Spain | 18964.13(12587.25,27050.20) | 329.62(218.78,470.17) |  | 19753.33(9663.99,36400.80) | 425.76(208.30,784.58) |  | 4.16(-32.57,60.66) | 1.34(0.71,1.97) |
| Sri Lanka | 3394.79(1825.13,5609.70) | 90.16(48.47,148.99) |  | 4714.08(2380.00,8520.58) | 133.23(67.26,240.80) |  | 38.86(-0.64,92.38) | -0.06(-0.46,0.34) |
| Sudan | 9491.41(4947.87,15667.57) | 175.39(91.43,289.52) |  | 25440.95(12558.39,43874.63) | 232.33(114.69,400.67) |  | 168.04(102.87,246.74) | 0.34(0.07,0.62) |
| Suriname | 83.72(45.08,136.13) | 96.83(52.14,157.44) |  | 172.63(91.07,293.24) | 174.84(92.24,297.01) |  | 106.19(51.39,172.74) | 0.58(0.22,0.94) |
| Sweden | 1535.68(870.32,2391.26) | 156.48(88.68,243.66) |  | 2573.68(1376.19,4206.32) | 207.97(111.20,339.89) |  | 67.59(31.86,117.33) | 0.22(-0.16,0.60) |
| Switzerland | 1429.12(764.76,2308.98) | 188.71(100.98,304.89) |  | 1997.87(1040.25,3370.97) | 224.35(116.82,378.55) |  | 39.80(8.06,78.93) | -0.08(-0.37,0.21) |
| Syrian Arab Republic | 6620.21(3394.73,11043.41) | 175.83(90.16,293.31) |  | 7376.67(3615.81,12697.71) | 277.50(136.02,477.67) |  | 11.43(-15.07,42.54) | 0.73(0.37,1.10) |
| Taiwan (Province of China) | 2007.51(1032.44,3370.47) | 51.45(26.46,86.39) |  | 1130.44(510.12,2033.19) | 55.02(24.83,98.96) |  | -43.69(-61.37,-23.57) | -0.29(-0.42,-0.16) |
| Tajikistan | 927.95(464.10,1539.09) | 67.41(33.72,111.81) |  | 2248.99(1087.32,3815.11) | 100.14(48.41,169.87) |  | 142.36(79.02,218.02) | 0.39(0.09,0.70) |
| Thailand | 10449.46(5535.12,16998.04) | 89.63(47.48,145.80) |  | 7618.33(3607.80,12929.06) | 109.79(51.99,186.32) |  | -27.09(-45.90,-0.27) | -0.07(-0.29,0.15) |
| Timor-Leste | 152.01(79.73,246.99) | 78.94(41.40,128.25) |  | 393.71(185.92,672.90) | 117.25(55.37,200.39) |  | 159.00(92.81,244.99) | 0.46(0.19,0.73) |
| Togo | 932.63(487.89,1525.76) | 86.66(45.33,141.77) |  | 2165.02(1114.47,3714.37) | 101.33(52.16,173.85) |  | 132.14(72.29,214.16) | 0.22(0.06,0.39) |
| Tokelau | 0.26(0.13,0.43) | 62.92(32.07,105.12) |  | 0.26(0.12,0.47) | 89.66(41.65,159.90) |  | 2.28(-25.05,33.54) | 0.41(0.12,0.70) |
| Tonga | 17.70(8.87,28.54) | 66.72(33.43,107.56) |  | 19.58(9.36,33.22) | 79.55(38.01,134.97) |  | 10.62(-20.80,52.19) | 0.07(-0.08,0.22) |
| Trinidad and Tobago | 220.94(111.81,371.80) | 80.93(40.96,136.19) |  | 281.86(138.54,478.05) | 146.87(72.19,249.11) |  | 27.57(-8.14,72.22) | 0.17(-0.26,0.60) |
| Tunisia | 4632.97(2473.11,7418.33) | 227.29(121.33,363.94) |  | 8251.05(4226.89,13933.79) | 440.36(225.59,743.65) |  | 78.09(35.42,131.01) | 1.11(0.76,1.47) |
| Turkey | 25603.45(14082.73,40126.08) | 189.18(104.06,296.49) |  | 42000.93(22273.11,70489.82) | 323.87(171.75,543.54) |  | 64.04(17.21,139.10) | 1.55(1.17,1.93) |
| Turkmenistan | 685.81(371.80,1111.19) | 74.93(40.62,121.41) |  | 1011.55(504.15,1712.95) | 102.77(51.22,174.03) |  | 47.50(6.24,102.50) | 0.32(0.05,0.59) |
| Tuvalu | 1.29(0.63,2.15) | 65.43(31.97,109.46) |  | 2.13(1.02,3.78) | 87.16(41.89,154.49) |  | 65.60(18.59,126.77) | 0.47(0.34,0.60) |
| Uganda | 7028.98(3672.41,11471.55) | 145.58(76.06,237.60) |  | 24754.09(11993.96,40714.98) | 197.75(95.81,325.25) |  | 252.17(161.09,365.06) | -0.95(-1.60,-0.29) |
| Ukraine | 5561.51(2905.58,8672.81) | 73.12(38.20,114.03) |  | 5542.11(2847.43,9446.20) | 116.60(59.91,198.74) |  | -0.35(-27.05,28.34) | -0.16(-0.64,0.33) |
| United Arab Emirates | 533.27(272.81,883.68) | 147.54(75.48,244.49) |  | 1963.98(996.74,3283.68) | 216.74(110.00,362.39) |  | 268.29(175.05,388.16) | 0.39(0.12,0.65) |
| United Kingdom | 13505.32(7736.68,20971.63) | 190.78(109.29,296.25) |  | 20351.98(11610.24,32270.02) | 250.38(142.84,397.00) |  | 50.70(44.10,58.05) | -0.25(-0.72,0.22) |
| United Republic of Tanzania | 7282.36(3771.72,11687.98) | 100.20(51.90,160.83) |  | 19578.13(10011.52,33544.91) | 125.85(64.35,215.63) |  | 168.84(97.55,270.61) | 0.21(-0.01,0.44) |
| United States of America | 15.29(7.82,26.10) | 73.03(37.34,124.63) |  | 10.22(5.09,17.28) | 107.92(53.76,182.44) |  | -33.17(-51.50,-7.87) | 0.44(0.14,0.74) |
| United States Virgin Islands | 63200.68(37479.32,94463.40) | 174.78(103.65,261.24) |  | 166685.76(105090.64,246893.38) | 408.14(257.32,604.54) |  | 163.74(133.05,219.55) | 2.51(2.00,3.03) |
| Uruguay | 659.68(347.71,1085.90) | 120.88(63.71,198.98) |  | 884.80(429.16,1513.55) | 190.07(92.19,325.13) |  | 34.13(0.37,77.17) | 0.57(0.27,0.88) |
| Uzbekistan | 3753.17(1974.02,6274.54) | 72.42(38.09,121.07) |  | 5896.58(3003.30,9867.38) | 94.26(48.01,157.73) |  | 57.11(13.78,112.33) | 0.29(0.04,0.55) |
| Vanuatu | 27.07(14.15,44.50) | 65.94(34.48,108.42) |  | 60.36(29.51,102.08) | 81.14(39.67,137.22) |  | 122.99(62.47,205.58) | 0.08(-0.09,0.25) |
| Venezuela (Bolivarian Republic of) | 2684.73(1379.56,4355.50) | 58.83(30.23,95.45) |  | 3297.64(1508.43,5660.84) | 74.29(33.98,127.53) |  | 22.83(-12.70,68.33) | 0.34(0.15,0.53) |
| Viet Nam | 10819.47(5648.01,18055.88) | 63.26(33.02,105.57) |  | 14825.45(6823.27,25826.48) | 89.20(41.05,155.39) |  | 37.03(-3.02,89.22) | 0.11(-0.19,0.41) |
| Yemen | 7689.02(3819.69,12704.18) | 178.08(88.46,294.23) |  | 18064.93(8968.53,31975.04) | 198.71(98.65,351.71) |  | 134.94(81.98,211.52) | 0.12(-0.04,0.27) |
| Zambia | 1968.72(1032.90,3267.61) | 87.68(46.00,145.53) |  | 6454.70(3238.22,11286.35) | 120.73(60.57,211.10) |  | 227.86(145.26,340.45) | 0.18(-0.08,0.44) |
| Zimbabwe | 1896.92(982.88,3076.58) | 62.14(32.20,100.78) |  | 3634.70(1851.86,6183.56) | 89.00(45.34,151.40) |  | 91.61(35.46,159.68) | 0.22(-0.07,0.50) |
